# PLK2 Plays an Essential Role in High D-Glucose-Induced Apoptosis, ROS Generation and Inflammation in Podocytes

**DOI:** 10.1038/s41598-017-00686-8

**Published:** 2017-06-27

**Authors:** Hong-hong Zou, Ping-ping Yang, Tian-lun Huang, Xiao-xu Zheng, Gao-si Xu

**Affiliations:** 1 Department of Nephrology, the Second Affiliated Hospital of Nanchang University, No. 1 Minde Road, Nanchang, 330006 P.R. China; 2 Department of Medicine, the George Washington University, Washington, DC20052 USA

## Abstract

Diabetic kidney disease (DKD) is a serious complication of hyperglycemia. Currently, there is no effective therapeutic intervention for DKD. In this study, we sought to provide a set of gene profile in diabetic kidneys. We identified 338 genes altered in diabetes-induced DKD glomeruli, and *PLK2* exhibited the most dramatic change. Gene set enrichment analysis (GSEA) indicated multiple signaling pathways are involved DKD pathogenesis. Here, we investigated whether PLK2 contributes to podocyte dysfunction, a characteristic change in the development of DKD. High D-glucose (HDG) significantly increased PLK2 expression in mouse podocytes. Suppressing *PLK2* attenuated HDG-induced apoptosis and inflammatory responses both *in vitro* and *in vivo*. NAC, an antioxidant reagent, rescued HDG and *PLK2* overexpression-induced kidney injuries. In summary, we demonstrated that silencing *PLK2* attenuates HDG-induced podocyte apoptosis and inflammation, which may serve as a future therapeutic target in DKD.

## Introduction

Currently more than 350 million people are suffering from diabetes mellitus. Diabetic kidney disease (DKD) is among the most serious complications of both type 1 and type 2 diabetes. DKD is the leading cause of kidney failure/end-stage renal disease^1^. In the early onset of DKD, it is primarily a glomerular disease and podocyte injury occurs even before albuminuria^2,3^. Hyperlycemia induces morphological changes in podocytes, including reduces slit diaphragms and shortens foot processes^4,5^. Decrease in podocyte number is the earliest cellular alteration in DKD, podocytes may detach from the glomerular basement membrane^6,7^. However, the molecular mechanisms underlying podocyte loss in DKD remain unknown.

Podocyte apoptosis contributes to podocyte loss in nondiabetic glomerulopathy mice, but it has not been verified in humans or other DKD animal models^8^. The correlation between podocytes apoptosis and albuminuria is not reported. Albuminuria is a major symptom in DKD animal models^9^. Recent studies have implicated that kidney inflammatory is involved in DKD progression^10,11^. Proinflammatory cytokines, including TNF-α, IL-6 and IL-1β, stimulate resident renal cells to produce other chemokines^12^. High glucose in diabetic patients increases chemokines, including CXCL1, CXCL8 and CXCL10.

RNA-sequencing (RNA-seq) is for a new methodology to analyze gene expression, which provides quantitative analysis for transcriptomes^13,14^. RNA-seq has been widely utilized for molecular classification and identification of biomarkers. Using RNA-seq, many genes have been implicated in DKD development and progression, such as miRNA and growth factors^15–17^. Bone morphogenetic protein-7 (BMP-7) is decreased in diabetic rats^18^, which is protective for podacytes through inhibiting TGF-β signaling^19^.

Upregulation of reactive oxygen species (ROS) due to mitochondrial defects can cause cell damage. Polo-like kinase 2 (*PLK2*) is essential cell survival during oxidative stress^20^. The PLK2 antioxidant activity is mediated by GSK3 phosphorylation to prevent p53-/ROS-induced necrosis^21^. However, whether PLK2 is involved in DKD pathogenesis is unclear. We sought to explore the hypothesis that PLK2 plays a critical role in podocyte survival in DKD progression.

In the current study, we presented gene profiles in glomeruli from DKD patients and diabetic rats. We found that PLK2 is up-regulated in DKD and we examined its biological function *in vitro* and *in vivo*. We demonstrated that PLK2 regulates glomerulosclerosis, cytokine release, and podocyte injury by aggravating inflammation responses and promoting oxidant stress and apoptosis. Our results provided that PLK2 may serve as a DKD biomarker and can be a future target to study DKD pathogenesis.

## Materials and Methods

### Animals

The male Sprague Dawley rats (weight, 150–180 g; 6-week-old, six per group) were purchased from the Shanghai BK Experimental Animal Center (Shanghai, China) and received either 60 mg/kg streptozotocin (STZ) or vehicle intraperitoneally. Animal tissues were collected after establishing the diabetic model (8 weeks after the first STZ administration, 4 weeks after letivirus injection). Diabetic was defined when the blood glucose exceeded 16.7 mmol/l at 48 h after STZ administration. At each end point as indicated, rats were euthanized, and glomeruli were collected as previous report^22^ for following RNA-seq and bioinformatics analysis. Animal use and welfare following a protocol reviewed and approved by the Second Affiliated Hospital, Nanchang University.

shPLK2 or negative control (shNC) lentivirus (100 ng/kg) was injected into rats (three per group). 4 weeks later, the inflammatory cytokines in peripheral blood were measured by ELISA. Rats were euthanized, and the glomeruli tissues were collected to perform TUNEL and immunohistochemistry staining. All methods were performed in accordance with the relevant guidelines and regulations of the Second Affiliated Hospital, Nanchang University.

### RNA-seq and bioinformatics analysis

Animal tissues were collected after establishing the diabetic model as before. RNA-seq was performed as previously described^23^. Data were normalized by log_2_. Glomeruli samples from diabetic rats have been deposited in NCBI (http://www.ncbi.nlm.nih.gov/sra, AC: SRP066646). DKD glomeruli and glomeruli in control group were collected from diabetic rats or human patients in a public database, National Center for Biotechnology Information Gene Expression Omnibus (GSE30122), including glomerulus of control kidney (n = 1222), flomerulus of DKD kidney (n = 1036), tubuli of control kidney (n = 1066) and tubuli of DKD kidney (n = 1299). Changes in gene expression above 1.5-fold with *P*-value less than 0.05 were considered as statistical significance. Gene set enrichment analysis (GSEA) was performed for identify signaling enriched between PLK2 expression. FDR ≤ 0.25, a well-established cut-off, was chosen to identify relevant genes in control and treated groups.

### Cell culture

Mouse podocytes were purchased from the Institute of Biochemistry and Cell Biology (Shanghai, China). Cells were cultured in RPMI-1640 (Hyclone, Logan, Utah, USA) with 10% fetal bovine serum (FBS, Gibco, Rockville, MD, USA), 1% penicillin-streptomycin solution (Solarbio, Bejing, China) and 10 U/ml IFN-γ (ProSpec-Tany Technogene Ltd, East Brunswick, NJ, USA). Cells were incubated at 33 °C with 5% CO_2_. When cells reached 70–80% confluence, culture media was switched to RPMI-1640 complete medium without 10 U/ml IFN-γ, and cells were incubated at 37 °C with 5% CO_2_ for another 10 to 14 days. Podocytes were cultured in the presence of D-glucose (DHG, 10, 20, 30 and 50 mM). D-glucose (DG, 5 mM, physiological concentration) was used as the control group. To compare the D-glucose and L-glucose effects, podocytes incubated with DG (5 mM) + LHG (25 mM) or DG (5 mM) + D-mannitol (DM, 25 mM) to keep the same osmolarity.

### Immunofluorescence

To identify mouse podocytes in our cultured condition, Nephrin expression in cultured mouse podocytes was measured by immunofluorescence. Mouse podocytes were incubated at 37 °C with 5% CO_2_ for another 10 to 14 days and incubated with antibody Nephrin (1:1000; Abcam, Cambridge, MA, USA) overnight at 4 °C, washed six times with PBS, incubation with the corresponding fluorescein isothiocyanate-conjugated secondary antibody. The nuclei were then stained with 4′,6-diamidino-2-phenylindole. The fluorescence signal was examined with an Olympus fluorescent microscope (BX 51, Olympus America, New York, USA) at magnification × 200.

### Plasmids

pLV-IRES-eGFP, pLKO.1-EGFP, psPAX2, pMD2G were purchased from Addgene (Cambridge, MA, USA). PLK2 was purchased from Sangon Biotech Co., Ltd. (Shanghai, China). PLK2 shRNA (GGTCTTCAGTTTCTTTACT) and scramble shRNA were synthesized from Sangon Biotech Co., Ltd. Oligonucleotides were annealed and digestedusing *Age* I and *Eco*R I, and constructed into pLKO.1-EGFP vector. pLV-IRES-eGFP-PLK2 was constructed using restriction enzymes *Bam*H I and *Eco*R I.

### Lentiviral preparation and infection in cell cultures

PLK2 and PLK2-shRNA were induced into mouse podocytes by using the lentiviruses. Briefly, 239 T cells were seeded in 60 mm dishes and after 24 h were transfected with 2 μg of the plasmid vector, 1 μg pLV-IRES-eGFP-PLK2/pLKO.1-EGFP-PLK2, 0.9 μg psPAX2 and 0.1 μg pMD2G using lipofectamine 2000 (Invitrogen Life Technologies). pLKO.1-EGFP-PLK2 (shPLK2) and pLV-IRES-eGFP-PLK2 (pPLK2) were collected 48 h after transfection and used to infect mouse podocytes. pLKO.1-EGFP-scramble (shNC) and black pLV-IRES-eGFP were control groups.

### Cell viability assay

Mouse podocytes infected with pLKO.1-EGFP-PLK2 (shPLK2) were plated in 96-well plates at 5 × 10^3^ cells/well, and cultured with various glucose conditions. Podocyte proliferation was measured by Cell Counting kit-8 (CCK-8, Dojindo Laboratories, Kumamoto, Japan) assay according to the manufacture’s instruction. Briefly, 10 μl CCK-8 was added at 0, 24, 48 and 72 h after HDG treatment and incubated for another hour at 33 °C with 5% CO_2_. Absorbance at 450 nm excitation was obtained using a microplate reader (Bio-Rad).

### Cell cycle analysis

Mouse podocytes were harvested and incubated with propidium iodide (PI, Sigma-Aldrich), and were analyzed by a flow cytometer (BD Biosciences, Franklin Lakes, NJ, USA). Briefly, 4 × 10^3^ of shPLK2- or pPLK2-infected mouse podocytes were collected and fixed in 70% ethanol at −20 °C overnight. The cells were re-suspended in 20 μg/ml PI and 200 μg/ml RNase A before flow cytometry analysis.

### Apoptosis analysis

After 12 h in HDG incubation, 4 × 10^3^ of shPLK2- or pPLK2-infected mouse podocytes were collected and incubated with annexin V-fluorescein isothiocyanate (FITC) and PI, prior to flow cytometry analysis.

### Reactive oxygen species and mitochondria membrane potential measurement

Mouse podocytes infected with shPLK2 or pPLK2 were incubated with HDG for 1 h and were plated in 6-well plates (1 × 10^5^ cells/well) and analyzed by a flow cytometer. For reactive oxygen species (ROS) assay, mouse podocytes were incubated with 10 μM DCFH-DA fluorescent probe (Beyotime Biotechnology, Shanghai, China) for 20 min in dark at 37 °C. For mitochondria membrane potential (MMP) assay, mouse podocytes were incubated with 0.5 ml Tetrechloro-tetraethylbenzimidazol carbocyanine iodide (JC-1, Immunochemistry Technologies, Bloomington, MN, USA) at 37 °C for 20 min.

### Enzyme linked immunosorbent assay

TNF-α, IL-1β, IL-6, COX-2 and CXCL1 in HDG-treated mouse podocytes or in peripheral blood were measured using commercially available murine-specific sandwich enzyme-linked immunosorbent assay (ELISA) kit (JRDUN Biotechnogy, Shanghai, China).

### Real-time PCR

Total RNA was extracted by Trizol (Invitrogen Life Technologies, Carlsbad, CA, USA). Briefly, 1 μg RNA was used to synthesize cDNA using a cDNA synthesis kit (Thermo Fisher Scientific, Rockford, IL, USA). *PLK2*, *SIRT5*, *Bcl-2*, *Bax*, cleaved *caspase-3* and *p53* were measured by SYBR Green (Takara Biotechnology Co., Ltd., Dalian, China), and Real-time PCR was performed using ABI-7300 (Applied Biosystems, Shanghai, China). The gene expression was calculated by the 2^−ΔΔCt^. Primers were list in Table 1. Gene expression was normalized to GAPDH.

**Table 1:** Primes sequences used in this study.

Gene	Sequences
*PLK2*-forward	5′-GCCAGAAGTCCGATACTACC-3′
*PLK2*-reverse	5′-TGATTCACAGCCGTGTCC-3′
*SIRT5*-forward	5′-CTCAAGACGCCAGAATCC-3′
*SIRT5*- reverse	5′-TCCACCTCCTCCAGAATG-3′
*Bax*-forward	5′-TTGCTACAGGGTTTCATC-3′
*Bax*-reverse	5′-ATTGCTGTCCAGTTCATC-3′
*Bcl-2*-forward	5′-TGGGCATAGATGTGTCAGG-3′
*Bcl-2*-reverse	5′-CCATATTCATCGCGTGGAG-3′
*p53*-forward	5′-CGTGCTCACCCTGGCTAAAG-3′
*p53*-reverse	5′-TGCTGGGAAGGAGGAGGATG-3′
*caspase-3*-forward	5′-CTGACTGGAAAGCCGAAAC-3′
*caspase-3*-reverse	5′-GCAAAGGGACTGGATGAAC-3′
*GAPDH*-forward	5′-ATCACTGCCACCCAGAAG-3′
*GAPDH*-reverse	5′-TCCACGACGGACACATTG-3′

### Terminal deoxynucleotidyl transferase-mediated dUTP nick-end labeling (TUNEL) assay

TUNEL staining was performed using Roche *In Situ* Cell Death Detection kit for programmed cell death (Medical & Biological Laboratories, Nagoya, Japan) according to the manufacturer’s instructions. TUNEL positive cells were counted under a microscope in five randomly chosen fields (×200) per dishusing NIH Image Software version 1.61.

### Immunohistochemistry

Sections were first treated for deparaffinization and hydration using dimethylbenzene and ethanol. Antigen retrieval was performed by incubating in 95°CEDTA (pH 8.0) buffer and followed by 3% hydrogen peroxide for 10 min. Primary antibodies PLK2, Nephrin and SIRT5 (1:1000; Abcam, Cambridge, MA, USA) were incubated for 1 h at room temperature. Sections were incubated by goat anti-mouse horseradish peroxidase-conjugated IgG (Abcam). Signals were visualized by DAB (Shanghai Long Island Biotec. Co., LTD, China) and hematoxylin staining (BASO, China). Five random fields per section were selected under the microscope to calculate percentage of positive cells.

### Protein extraction and western blot

Mouse podocytes or rat glomeruli were collected and incubated in RIPA buffer (Beyotime) containing 1 mM phenylmethylsulfonyl fluoride (PMSF) for 30 min on ice. Proteins were loaded on 12% SDS-PAGE gels and were transferred to polyvinylidene fluoride (PVDF) membranes (Sigma-Aldrich, St. Louis, MO, USA). Membranes were blocked in fat-free milk overnight at 4 °C and then incubated with primary antibodies overnight at 4 °C. Secondary antibody horseradish peroxidase-conjugated goat anti-rabbit/anti-mouse IgG (1:1,000; Beyotime Institute of Biotechnology, Haimen, China) was incubated 1 h at room temperature. The signals were visualized using enhanced chemiluminescence (EMD Millipore, Billerica, MA, USA), and band densitometry was quantified using Quantity One version 4.62 (Bio-Rad Laboratories, Inc., Hercules, CA, USA).

Primary antibodies include PLK2 (1:1000), cleaved caspase-3 (1:500) from Abcam; Bcl-2 (1:400) and Bax (1:400) from Santa Cruz Biotechnology, Inc. (Santa Cruz, CA, USA); SIRT5 (1:1000), GAPDH (1:1500), and p53 (1:1000) from Cell Signaling Technology, Inc. (Danvers, MA, USA).

### Statistical analysis

Data were presented as the mean ± SD. Statistical analysis was performed using GraphPad Prism 5 software (GraphPad Software, Inc., LaJolla, CA, USA). Statistical significance was analyzed by unpaired, two-tailed Student’s t-test. *P* < 0.05 was considered as statistical significance.

## Results

### Gene expression differed in DKD rat tissues

First, we compared the gene-expression difference between isolated control and diabetes-induced DKD glomeruli using RNA-seq analysis. We identified 340 out of 16880 transcripts exhibited distinct expression patterns between control and DKD tissues (Supplementary Table 1). Majority of the transcripts (214) were decreased in DKD glomeruli. Among these transcripts, the following genes decreased: *Scd*1 (7.99-fold), *Crygb* (5.57-fold), *Ifit1* (5.21-fold), and *Pbk* (4.72-fold). On the other hand, transcripts showed the highest increase were *Dmrtclc* (7.49-fold), *Kif5c* (6.09-fold), *RT1-Ba* (5.81-fold), and *Grem2* (4.52-fold). The SCD1 gene (*Scd1*) encodes a key enzyme regulating membrane fluidity and lipid metabolism, and had the most discriminating power between the diabetic NOD mice and control mice^24^. IFIT1 encodes the intracellular p56 protein, which inhibits protein synthesis^25^. KIF5C serve as a molecular motor to transport various cargos^26^. However, the roles of these transcripts in the DKD progression have not been reported and characterized. Our comprehensive analysis of different transcripts in DKD glomeruli provided further study of these transcript changes during DKD development.

### Multiple pathways were altered in DKD glomeruli in bioinformatic prediction

To determine which signaling pathways were altered in DKD glomeruli, we performed GSEA analysis using rat glomeruli (control and DKD)^27^. We generated a gene list with greatest changes using RNA-seq data, and the enrichment of pathway clusters was evaluated by GSEA. GSEA analysis indicated that 22 pathways were significantly altered in DKD tissues, with *P* < 0.05. Seven pathways were enriched in DKD group, including cardiac muscle contraction, dilated cardiomyopathy, tight junction and o-glycan biosynthesis. The complete list of pathways (and the corresponding genes) was shown in Table 2. Control group had 15 signaling pathways enriched, including the cell cycle, oocyte meiosis, Toll-like receptor signaling, and progesterone-mediated oocyte maturation (Table 3).

**Table 2:** Selected enriched pathways in DKD glomeruli.

Pathway	NES	*P*-value	FDR q-value	Molecules
Cardiac muscle contraction	1.70E + 00	1.95E – 03	2.50E – 01	CACNA1C, TNNT2, ACTC1, MYH6, RYR2, ATP1A3, MYH7, CACNA1D, CACNB3, TPM2, CACNA2D1, CACNA2D4, and MYL2
Dilated cardiomyopathy	1.67E + 00	2.04E – 03	1.86E – 01	CACNA1C, TNNT2, ITGA11, SGCA, ACTC1, MYH6, ADCY3, RYR2, ITGB4, MYH7, ADCY5, CACNA1D, TGFB3, DES, ADCY4, CACNB3, TPM2, CACNA2D1, and CACNA2D4
Tight junction	1.58E + 00	2.06E – 03	1.98E – 01	CLDN23, CLDN22, MYH6, MAGI2, MYH11, TJP3, MYH7, CLDN9, CLDN11, MYH3, PPP2R2C, CLDN6, MYH14, ACTN3, CLDN4, CLDN15, CLDN14, MYL2, PARD6A, MRAS, LLGL2, ACTN2, MAGI1, TJAP1, TJP2, PPP2R2B, MYH2, PRKCZ, ACTN1, SYMPK, PRKCE, AKT3, LLGL1, PRKCH, and CLDN17
Other types of o-glycan biosynthesis	1.63E + 00	7.97E – 03	1.99E – 01	CHST10, CHST10, B4GALT2, FUT4, LFNG, and MFNG
Basal cell carcinoma	1.60E + 00	1.01E – 02	2.15E – 01	WNT11, FZD9, WNT5B, APC2, BMP2, WNT3, WNT7B, WNT6, AXIN2, SHH, FZD2, WNT9B, WNT9A, FZD5, DVL2, HHIP, DVL1, AXIN1, WNT4, WNT5A, and PTCH1
Hypertrophic cardiomyopathy (HCM)	1.56E + 00	1.44E – 02	1.94E – 01	CACNA1C, TNNT2, ITGA11, SGCA, ACTC1, MYH6, RYR2, ITGB4, MYH7, PRKAB2, CACNA1D, TGFB3, DES, CACNB3, PRKAG3, TPM2, CACNA2D1, CACNA2D4, and IL6
Metabolism of xenobiotics by cytochrome p450	1.55E + 00	2.78E – 02	1.92E – 01	ADH4, GSTM5, GSTO2, ALDH3A1, CYP1A1, ADH7, ALDH1A3, ADH1, GSTM2, and MGST2

**Table 3:** Selected enriched pathways in normal control glomeruli.

Pathway	NES	*P*-value	FDR q-value	Molecules
Cell cycle	−1.98E + 00	0.00E + 00	6.80E – 03	MCM5, CDC25C, CHEK2, CDKN2C, PKMYT1, ANAPC10, ANAPC10, CDC25B, MCM4, CCNA1, MAD2L1, CDC20, DBF4, E2F1, RBL1, TGFB2, TTK, BUB1, CDC6, LK1, CDK1, PTTG1, CCNA2, CCNB2, and ESPL1
Oocyte meiosis	−1.79E + 00	0.00E + 00	4.01E – 02	FBXO5, FBXO5, CDC25C, PKMYT1, ANAPC10, CPEB1, MAD2L1, PGR, AURKA, CDC20, BUB1, IGF1, PLK1, CDK1, PTTG1, CCNB2, and ESPL1
Toll-like receptor signaling pathway	−1.66E + 00	0.00E + 00	1.36E – 01	TLR6, MAPK1, IKBKE, CASP8, IRF7, MAPK9, IL12A, CCL5, CD14, CD86, IRF5, TLR7, TLR4, MAP2K6, TLR1, TLR5, TLR2, CXCL9, PIK3R5, FOS, IL1B, and MAPK10
Progesterone-mediated oocyte maturation	−1.79E + 00	1.94E – 03	7.54E – 02	CDC25C, KRAS, PKMYT1, ANAPC10, CDC25B, CCNA1, CPEB1, MAD2L1, PGR, PIK3R5, BUB1, IGF1, PLK1, CDK1, MAPK10, CCNA2, and CCNB2
Influenza	−1.50E + 00	2.10E – 03	2.14E – 01	MAPK1, IFNGR2, IKBKE, IRF7, PYCARD, MAPK9, RNASEL, KPNA2, IL12A, CCL5, CIITA, NXT2, DDX58, NLRP3, TLR7, TLR4, RSAD2, TMPRSS4, PIK3R5, TMPRSS13, IL1B, MAPK10, and CCL12
Type I diabetes mellitus	−1.79E + 00	4.06E – 03	5.22E – 02	GZMB, CPE, GAD1, IL12A, GAD2, CD86, PTPRN2, IL1B, CD28, and PRF1
Leishmaniasis	−1.59E + 00	9.71E – 03	1.68E – 01	MAPK1, IFNGR2, ITGB2, IL10, MARCKSL1, IL12A, NCF2, CYBA, TLR4, TLR2, FOS, PTGS2, IL1B, TGFB2, and ITGAM
Malaria	−1.60E + 00	1.78E – 02	1.85E – 01	GYPC, CD40LG, ITGB2, IL10, SELP, IL12A, VCAM1, SELE, TLR4, HGF, TLR2, HBB-B1, ITGAL, IL1B, TGFB2, KLRK1, and CCL12
Homologous recombination	−1.58E + 00	1.81E – 02	1.57E – 01	RAD50, RAD51C, BRCA2, RAD54L, RAD51, XRCC2, RAD54B, and EME1
Rheumatoid arthritis	−1.50E + 00	1.98E – 02	2.21E – 01	CCL5, CTLA4, CCL20, CCL3, CD86, TLR4, TLR2, TNFSF13B, FOS, ITGAL, IL1B, TGFB2, ANGPT1, CD28, and CCL12
Tryptophan metabolism	−1.61E + 00	2.22E – 02	1.80E – 01	ACMSD, CCBL1, ALDH3A2, HADH, MAOA, IDO1, ALDH7A1, WARS2, KYNU, AOX1, DDC, OGDHL, MAOB, TPH1, INMT, and ALDH1B1
*Staphylococcus aureus* infection	−1.52E + 00	2.63E – 02	2.05E – 01	ITGB2, IL10, C1QC, SELP, PTAFR, CFI, C3AR1, C2, FCGR2B, ITGAL, ITGAM, and FGG
Systemic lupus erythematosus	−1.48E + 00	2.67E – 02	2.38E – 01	H2AFX, TROVE2, C1QB, HIST1H2AF, H3F3B, HIST3H2BA, SNRPD1, CD40LG, IL10, C1QC, HIST1H2AK, CD86, C2, HIST3H2A, FCGR2B, C8B, C6, and CD28
Steroid hormone biosynthesis	−1.55E + 00	2.71E – 02	1.92E – 01	CYP11B2, HSD17B7, CYP21 A1, CYP17A1, CYP7B1, HSD3B6, HSD17B2, and HSD17B1
Nicotinate and nicotinamide netabolism	−1.53E + 00	4.13E – 02	2.14E – 01	NMNAT2, NT5C1A, NMNAT3, NT5M, ENPP1, NUDT12, NT5E, AOX1, and CD38

### PLK2 upregulated in DKD glomeruli and regulated multiple pathways

**Figure 1:**
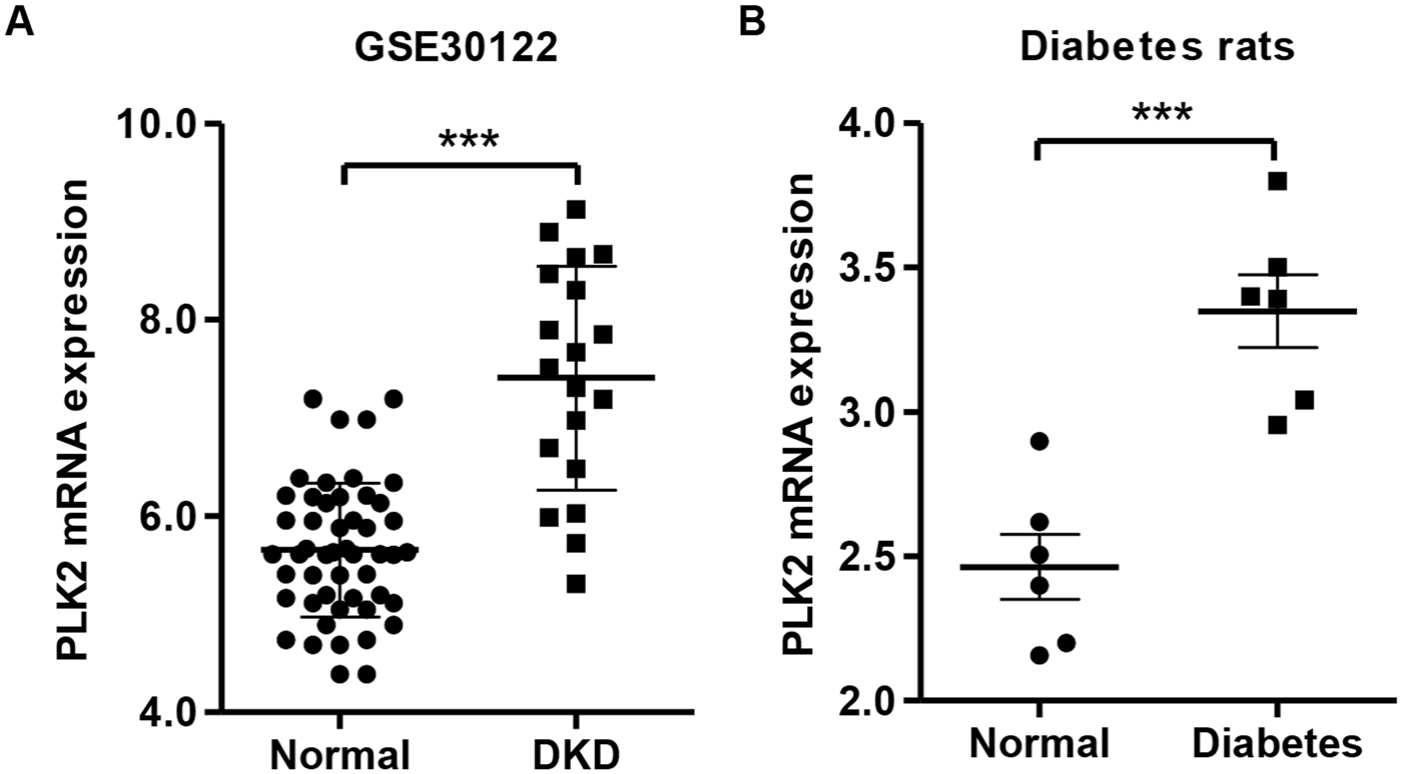
PLK2 up-regulation in DKD patients and diabetic rat models. (**A**) PLK2 expression in glomeruli and tubuli from DKD patients, gene data were from National Center for Biotechnology Information Gene Expression Omnibus (GSE30122). (**B**) Expression of PLK2 in glomeruli was significantly increased in diabetic rats. ***P < 0.001.

Changes in mitochondrial dysfunction and reactive oxidant species have been demonstrated in DKD progression in numerous studies^28,29^. We previously reported that PLK2 mediates defective mitochondrial changes and PLK2 activity is required for cell survival^20,21^. To investigate whether PLK2 is altered in DKD tissues, we performed the microarray and GSEA analysis. PLK2 increased dramatically in DKD kidneys compared to controls in GSEA30122 database (Fig. 1A). Based on PLK2 median content, kidney tissues were divided into PLK2 high and PLK2 low groups. Comparing the mRNA microarray data of PLK2 high group with PLK2 low group, we identified a list of genes that differ between PLK2 high vs. PLK2 low. The GSEA analysis indicated that 17 pathways were significantly enriched in PLK2 high groups (GSE30122), including cell cycle, p53 signaling, *Escherichia coli* infection and DNA mismatch repair. The complete list of pathways (and the corresponding molecules) was shown in Supplementary Table 2. The changes in multiple pathways due to PLK2 levels suggested that PLK2 is a key regulator in kidney. 17 pathways express abundantly in PLK2 low groups. The top pathways included the neuroactive ligand receptor, olfactory transduction, oxidative phosphorylation and maturity onset diabetes (Supplementary Table 3). RNA-seq analysis confirmed that PLK2 was up-regulated in diabetes-induced DKD rats (n = 6) compared with normal rats (n = 6) (Fig. 1B). These findings suggested that PLK2 regulates DKD procession and we next explored how PLK2 is involved in DKD pathogenesis.

### PLK2 was up-regulated in high D-glucose-induced podocytes and PLK2 knockdown increased podocyte viability

**Figure 2:**
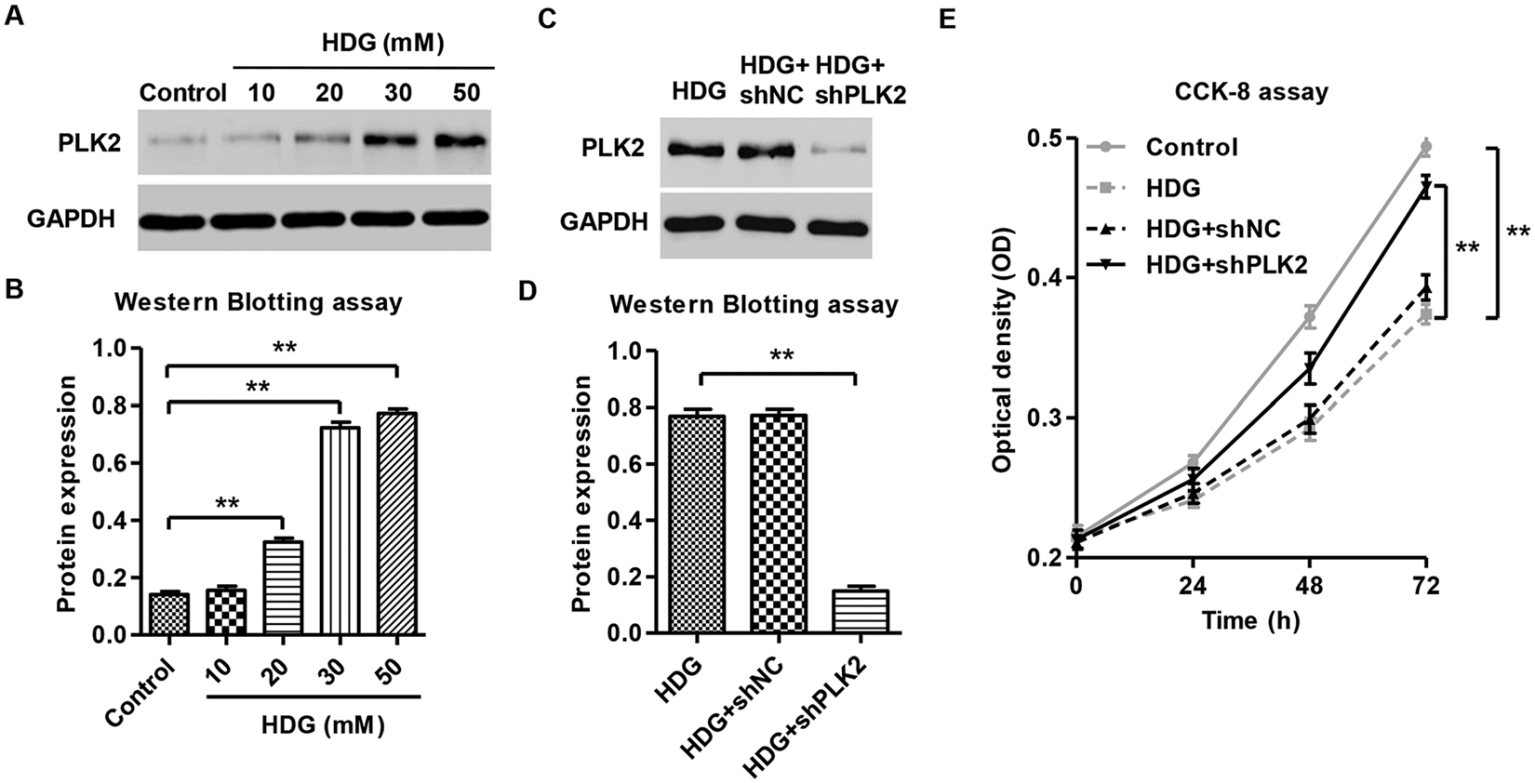
High D-glucose induced PLK2 expression. (**A,B**) Representative blots of PLK2 upregulation with different D-glucose concentrations. (**C,D**) Lentiviral encoding shRLK2 significantly reduces PLK2 expression in the presence of HDG in mouse podocytes. (**E**) Cell viability in response to HDG was significantly improved in shPLK2 group in mouse podocytes using CCK-8 assay. **P < 0.01.

Supplementary Figure 1 showed Nephrin expression in cultured mouse podocytes, suggesting that these are in fact podocytes with properties of podocytes *in vitro*. We first examined whether high D-glucose (HDG) affects PLK2 expression levels in mouse podocytes. The results showed that the expression of PLK2 was increased in the presence of HDG in a dose dependent manner (10, 20, 30 and 50 mM) (Fig. 2A and B).D-mannitol (DM) and high L-glucose (HLG) did not affect PLK2 protein expression (protein level: control 0.141 ± 0.010; DM 0.156 ± 0.013; HLG 0.149 ± 0.009, *P* > 0.05, data not shown). Since 30 mM HDG induce a dramatic PLK2 increase, we used this dose for further studies.

To elucidate endogenous PLK2 function in mouse podocytes, we suppressed PLK2 using PLK2 specific shRNAs. The results illustrated the efficiency and specificity of the PLK2 shRNAs (Fig. 2C and D**)**. shRNA control (shNC) did not alter PLK2 expression (protein level: HDG 0.769 ± 0.025; HDG + shNC 0.772 ± 0.022, *P* > 0.05). HDG exposure led to decreased podocyte viability (Fig. 2E). However, treatment with DM and HLG did not affect the viability of mouse podocytes, suggesting the toxic effects were HDG specific (data not shown). Interestingly, shPLK2 significantly increased mouse podocytes viability in the presence of HDG (Fig. 2E).

### PLK2 mediated HDG-induced cell cycle arrest and HDG-induced apoptosis in podocytes

**Figure 3:**
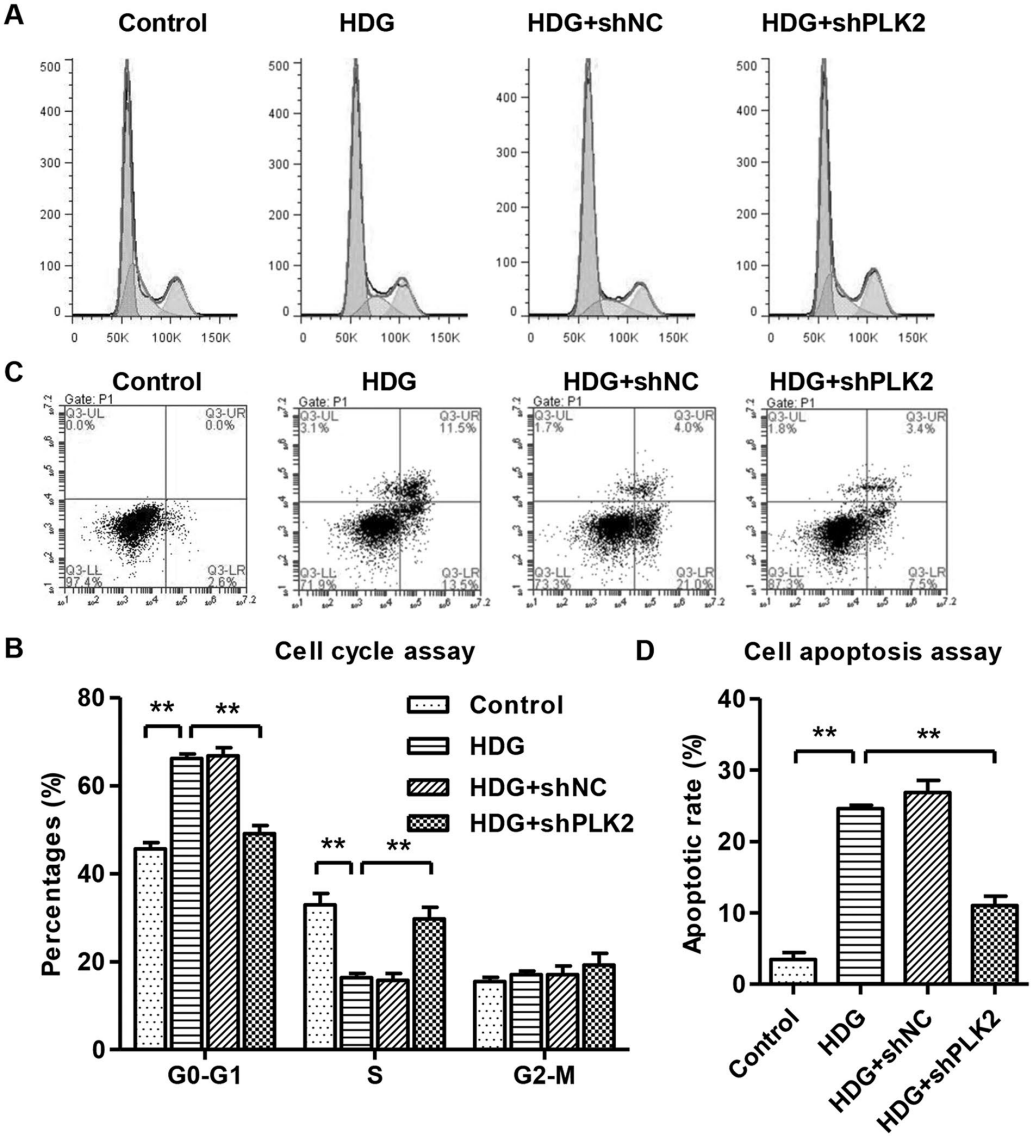
Knocking down PLK2 promoted S-phase entry and decreased apoptosis in mouse podocytes, reversing HDG effects. Podocytes were treated with 30 mM HDG for 12 h. (**A,B**) Cell cycle was measured by propidium iodide (PI) using flow cytometry analysis. (**C,D**) Cell apoptosis was measured by annexin V-fluorescein isothiocyanate (FITC) and PI, using flow cytometry. HDG induced G1 arrest and increased cell apoptosis, which were reversed by PLK2 knockdown. **P < 0.01.

Next, we explored whether PLK2 regulates cell cycle and apoptosis in response to HDG. HDG administration significantly increased G1 phase percentage and decreased S phase progression (Fig. 3A and B), indicating that HDG treatment promotes cell cycle arrest at G1 phase. These effects were dramatically attenuated when PLK2 was knockdown (Fig. 3A and B). HDG led to a significantly increase in podocyte apoptosis (Fig. 3C and D), confirming diabetic pathogenesis. Knocking down PLK2 partially rescued HDG mediated apoptosis (Fig. 3C and D). However, treatment with DM and HLG did not affect the cell cycle and apoptosis of mouse podocytes (data not shown). These results suggested that PLK2 knockdown suppresses HDG cytotoxicity, and endogenous PLK2 plays an essential role in HDG induced cellular toxicity.

### Knocking down PLK2 inhibited HDG-induced ROS production and MMP reduction

**Figure 4:**
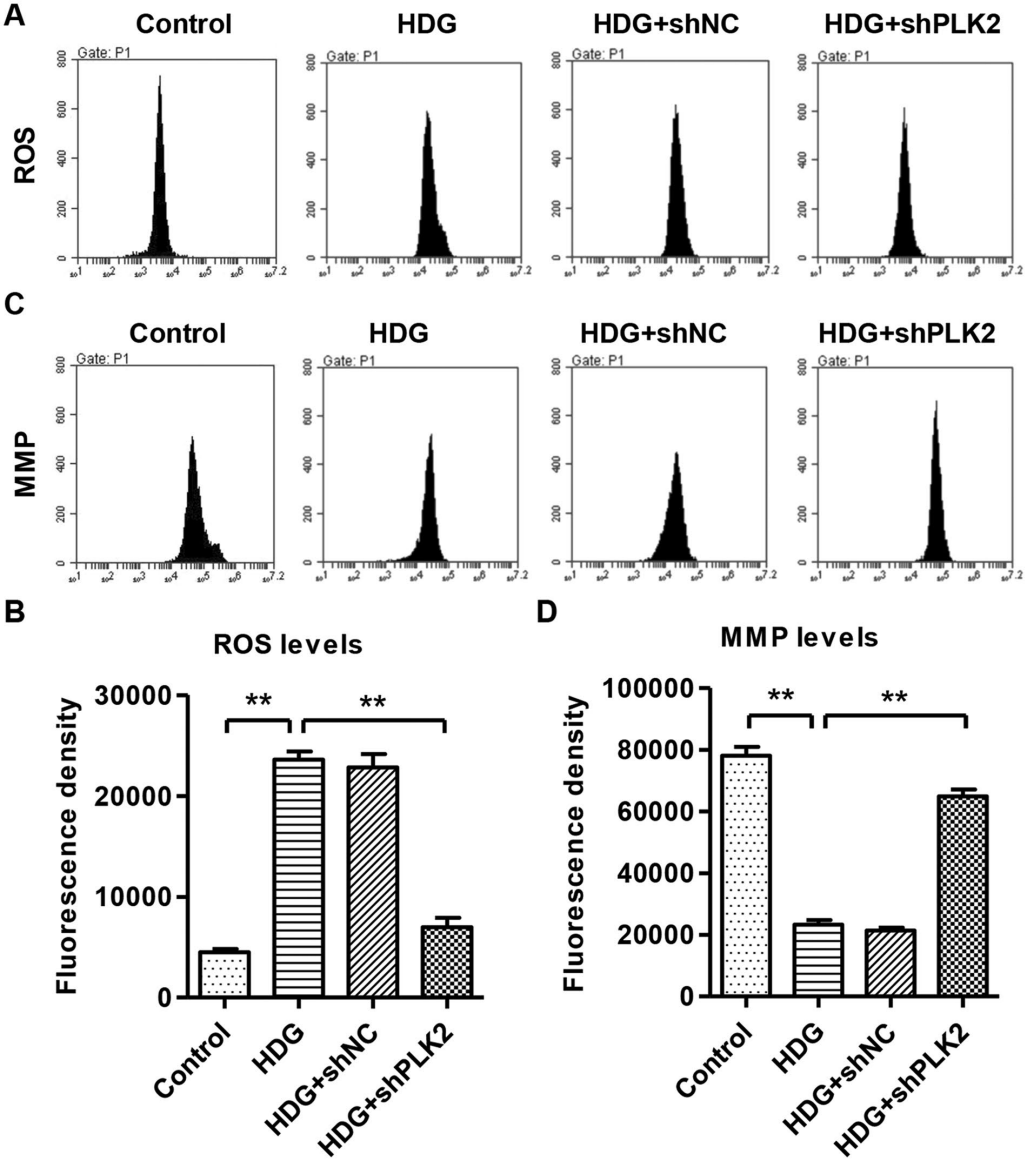
Knocking down PLK2 decreased ROS and increased MMP levels in mouse podocytes in response to high D-glucose (30 mM, 1 h). (**A,B**) ROS was measured by DCFH-DA fluorescent probe using flow cytometry. (**C,D**) MMP level was measured by JC-1 using flow cytometry. Suppressing PLK2 reversed HDG caused ROS accumulation and MMP decrease. **P < 0.01.

ROS accumulation and mitochondrial membrane potential (MMP) reduction are major biological consequences of mitochondria dependent apoptosis. To investigate whether PLK2 regulates apoptosis is associated with mitochondrial dysfunction, we utilized flow cytometry to measure ROS and MMP after HDG administration. As shown in Fig. 4A and B, HDG increased ROS production. PLK2 knockdown significantly decreased the HDG mediated ROS accumulation (Fig. 4A and B). In addition, MMP levels were significantly decreased in the presence of HDG, which was partially reversed by suppressing PLK2 (Fig. 4C and D). However, treatment with DM and HLG did not affect the ROS production and MMP level of mouse podocytes (data not shown). These results indicated that endogenous PLK2 contributes to HDG caused ROS production and MMP reduction in podocytes.

### Depletion of PLK2 eliminated HDG induced inflammatory cytokine accumulation and apoptotic markers

**Figure 5:**
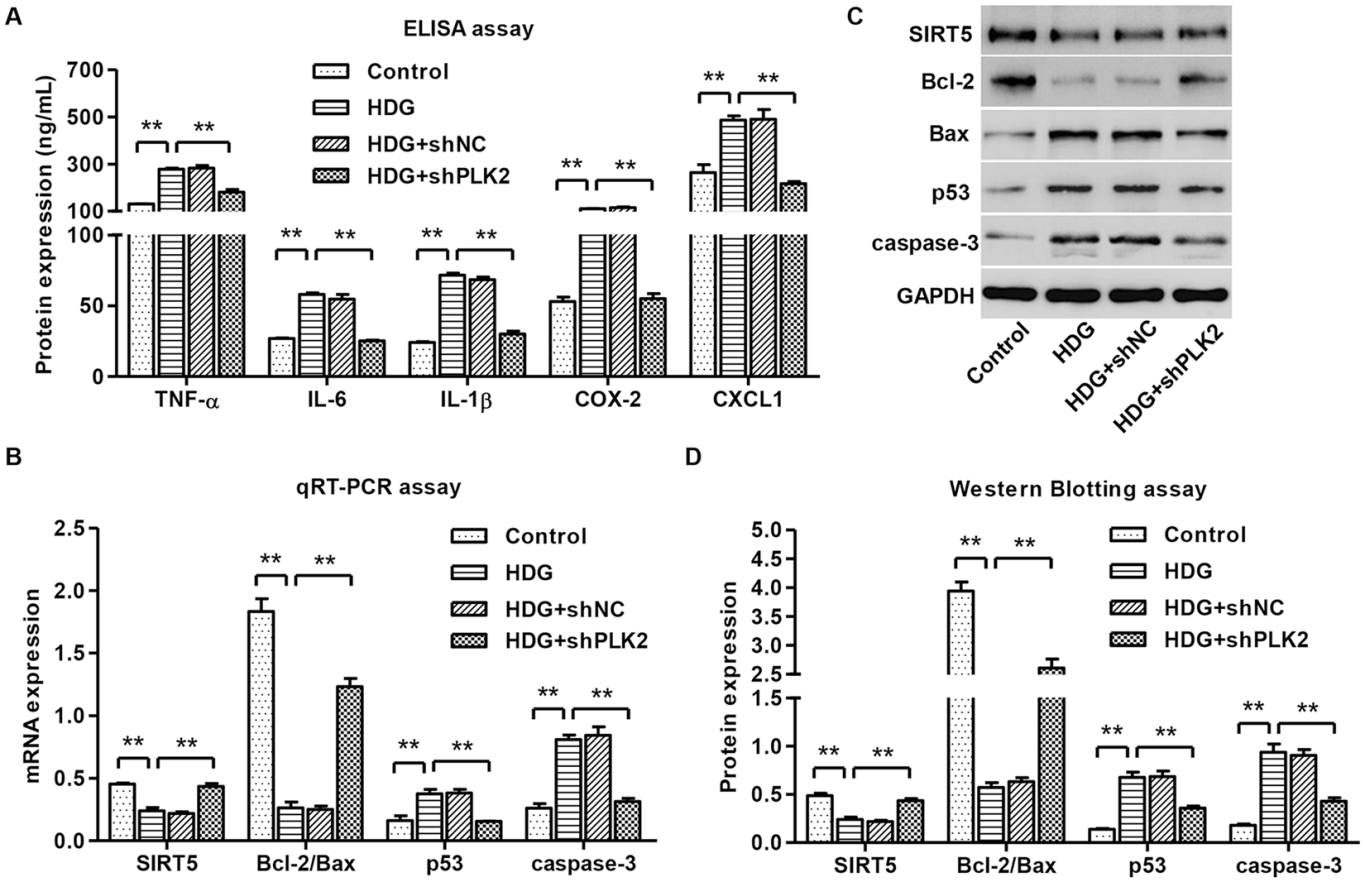
Knocking down PLK2 inhibited inflammatory responses and decreased apoptosis-associated markers. Mouse podocytes were treated with 30 mM HDG for 12 h or 24 h. (**A**) TNF-α, IL-6, IL-1β, COX-2 and CXCL1 were measured by ELISA assay. Apoptosis-associated markers SIRT5, Bcl-2/Bax, p53 and cleaved caspase-3 were measured by real-time PCR (**B**) and western blot analysis (**C,D**). HDG effects were reversed in shPLK2 group. **P < 0.01.

Proinflammatory cytokines are linked to diabetic development and regulate mitochondrial metabolism^30–32^. Therefore, we measured TNF-α, IL-6, IL-1β, COX-2 and CXCL1 secretions in response to HDG and we hypothesized that PLK2 is required for HDG caused cytokine changes. TNF-α, IL-6, IL-1β, COX-2 and CXCL1 were significantly up-regulated by HDG (Fig. 5A). Depletion of PLK2 blocked HDG-mediated TNF-α, IL-6, IL-1β, COX-2 and CXCL1 up-regulation (Fig. 5A), suggesting that HDG-mediated cytokine production is dependent on PLK2.

To explore the mechanisms of how PLK2 regulates apoptosis, we examined apoptosis-associated markers at both transcription and translation levels. As shown in Fig. 5B–D, HDG increased p53 and activated cleaved caspase-3, but decreased SIRT5 and the ratio of Bcl-2/Bax. Depleting PLK2 significantly reversed these effects (Fig. 5B–D).

### N-acetylcysteine (NAC) inhibited HDG and PLK2 overexpression-induced apoptosis, ROS production and MMP decrease of mouse podocytes

**Figure 6:**
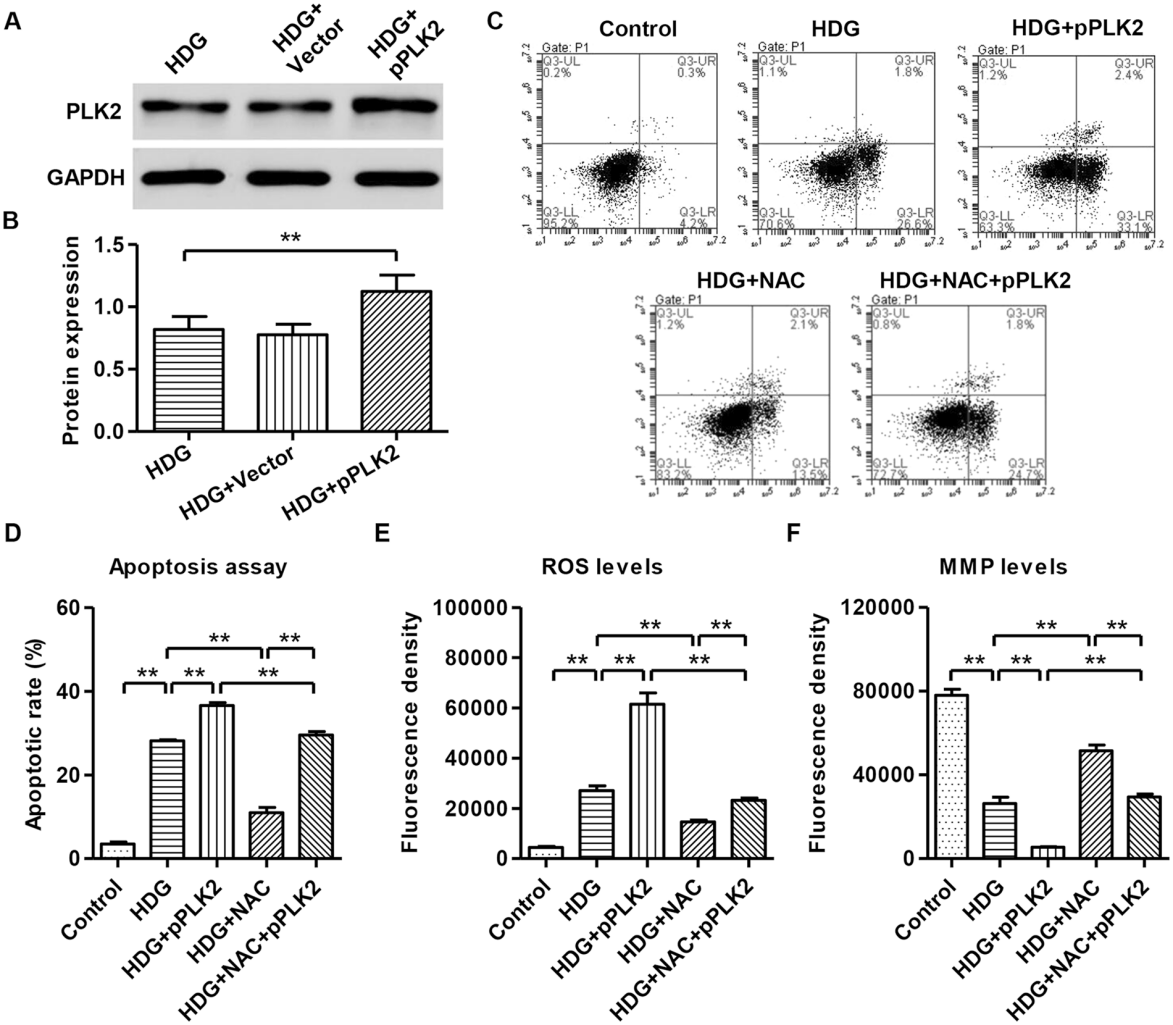
NAC blocked PLK2 overexpression effects on apoptosis, ROS generation, and MMP production. Mouse podocytes were treated with 30 mM HDG prior to 100 μM NAC treatment. (**A,B**) Overexpression of PLK2 at 24 h in the presence of HDG. (**C,D**) Cell apoptosis was measured by annexin V-fluorescein isothiocyanate (FITC) and PI, prior to analysis by a flow cytometry 12 h after HDG treatment. (**E**) ROS generation was measured by DCFH-DA fluorescent probe inflow cytometry1 h after HDG treatment. (**F**) MMP level was measured by JC-1 inflow cytometry1 h after HDG treatment. Overexpressing PLK2 exaggerated HDG effects. NAC attenuated HDG and PLK2 overexpressing effects. **P < 0.01.

We have shown that suppressing PLK2 reversed HDG actions in podocytes, next we examined PLK2 overexpression function using lentiviruses. As shown in Fig. 6A and B, our overexpression system was successful. Control plasmid pLV-IRES-eGFP did not affect PLK2 expression (protein level: control 0.819 ± 0.103; black vector 0.776 ± 0.085, *P* > 0.05). Consistent with PLK2 knockdown results, overexpressing PLK2 stimulated apoptosis and ROS production, and reduced MMP levels (Fig. 6C–F). N-acetylcysteine (NAC), an antioxidant, is able to mitigate the mitochondrial oxidative stress and apoptosis. NAC (100 μM) was added to podocytes, and we found that NAC blocked HDG and PLK2 overexpression induced apoptosis and ROS accumulation (Fig. 6C–F). In contrast, NAC increased MMP levels, reversing the effects of HDG or PLK2 overexpression. These data suggested that NAC ameliorates the mitochondrial defects caused by HDG and PLK2 overexpression.

### Effects of ROS scavenger on inflammatory factors and apoptosis-associated markers in HDG-induced mouse podocytes

**Figure 7:**
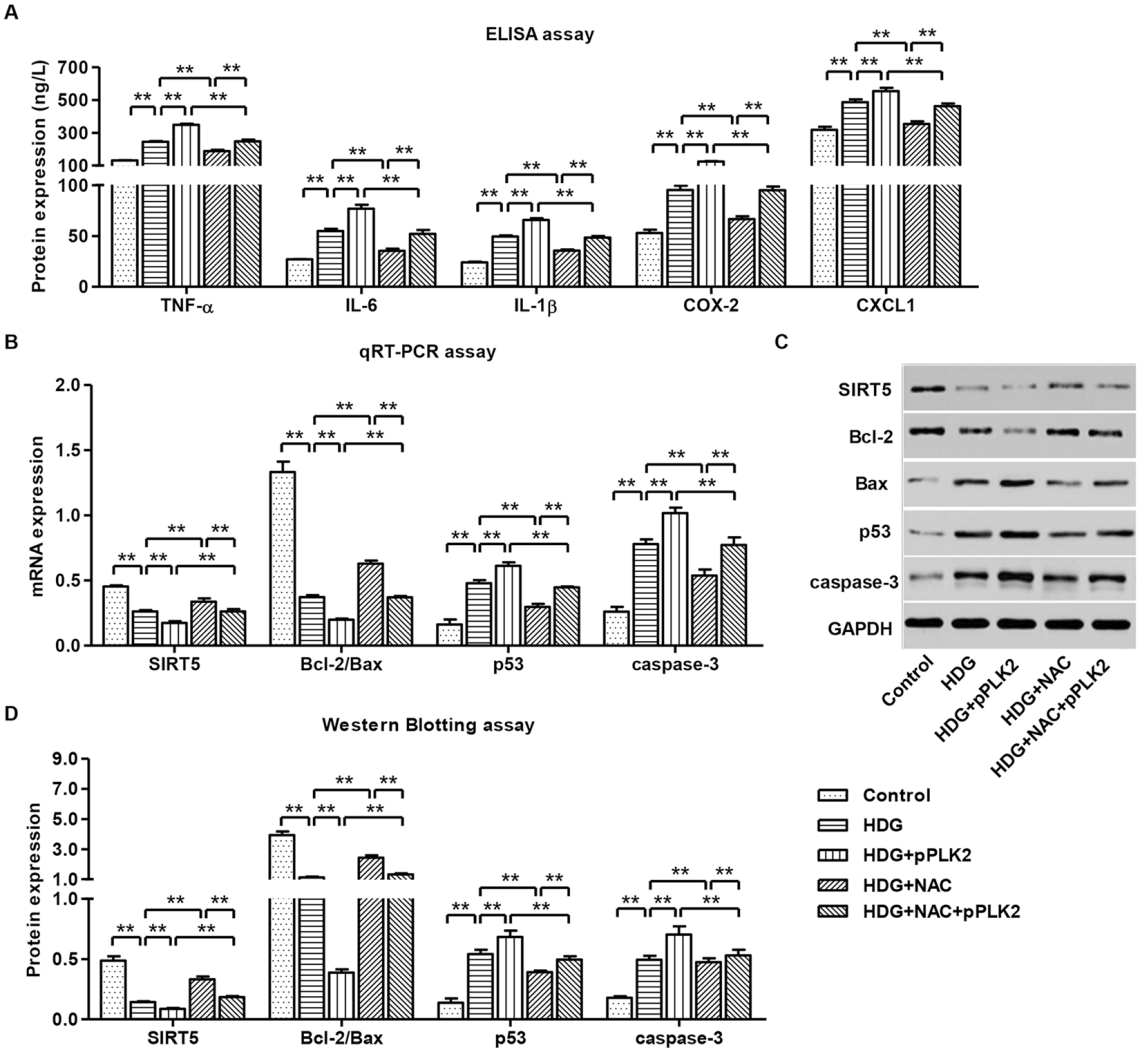
NAC blocked HDG and PLK2 overexpressing induced inflammatory responses and apoptosis. pPLK2-infected mouse podocytes were treated with 30 mM HDG prior to 100 μM NAC treatment. (**A**) Expression of inflammatory factors TNF-α, IL-6, IL-1β, COX-2 and CXCL1 12 h after HDG treatment. (**B**) mRNA levels of apoptosis markers SIRT5, Bcl-2/Bax, p53 and cleaved caspase-3 12 h after HDG treatment. Protein expression of apoptosis markers 24 h after HDG treatment (**C,D**). **P < 0.01.

To further characterize the role of PLK2/HDG on mouse podocytes, we examined whether NAC regulates inflammatory cytokine production and apoptosis marker expression. We found that overexpressing PLK2 increased of TNF-α, IL-6, IL-1β, COX-2 and CXCL1 levels, which was reversed by NAC administration (Fig. 7A). PLK2 overexpression decreased SIRT5 and the ratio of Bcl-2/Bax, and increased p53 and activated cleaved caspase-3; whereas NAC treatment rescued theses changes (Fig. 7B–D). These data suggested that PLK2 induced apoptosis and inflammation acts may partially through ROS signaling.

### PLK2 knockdown inhibited inflammatory factor releases and apoptosis in diabetes-induced DKD rats

**Figure 8:**
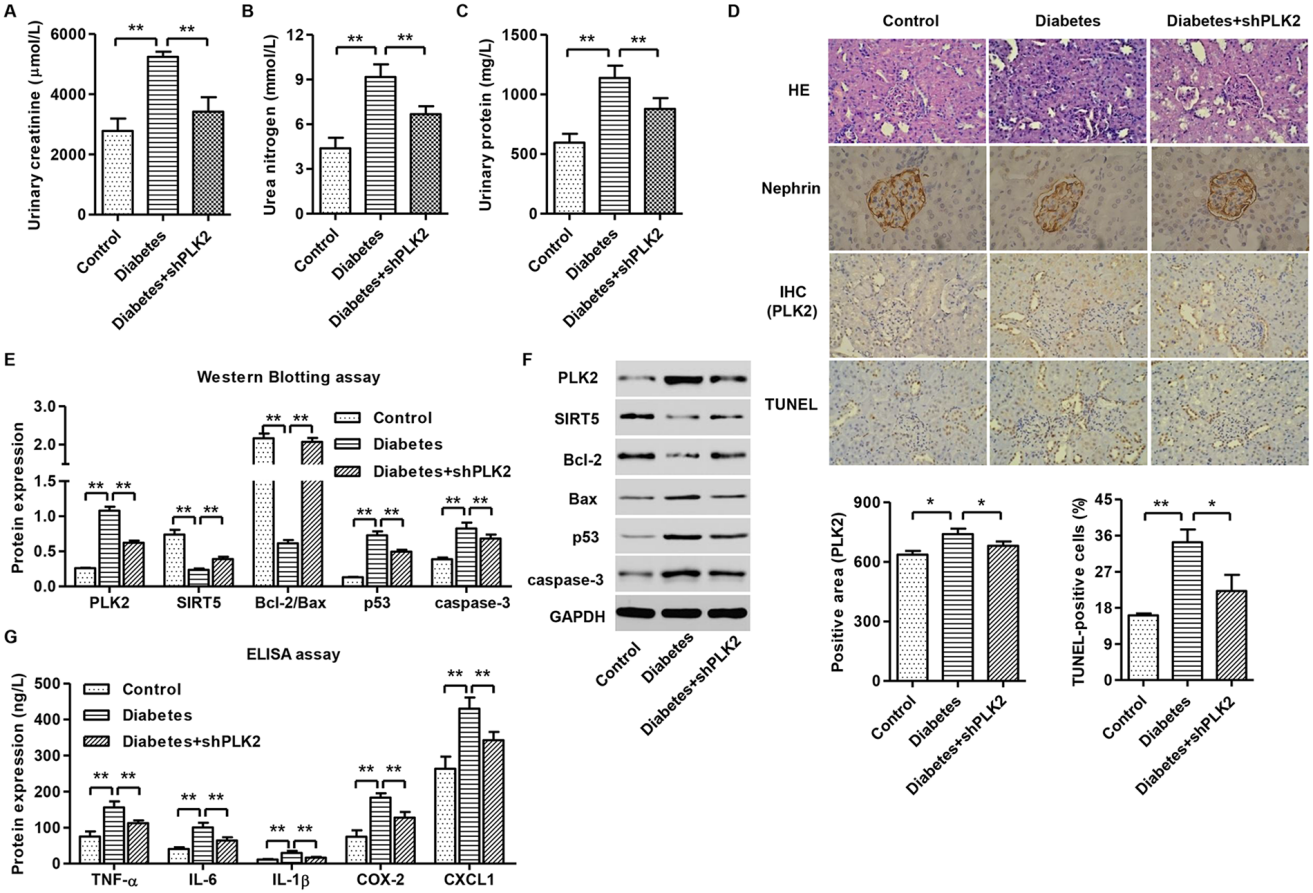
PLK2 knockdown suppressed diabetes-induced apoptosis and inflammatory responses in rats. Diabetic rats (n = 3) were analyzed 24 h after shPLK2 lentiviral injection. (**A–C**) The urinary levels of creatinine, nitrogen and protein in diabetic rats were measured by biochemical assay. (**D**) Glomeruli of diabetic rats with H&E staining, PLK2 immunohistochemistry, TUNEL assay, and Nephrin staining. (**E,F**) Quantitative analysis and representative images of western blot. PLK2 was up-regulated in diabetic rats. Expression of apoptosis-associated markers including SIRT5, Bcl-2/Bax, p53 and cleaved caspase-3 in rats was measured. (**G**) Inflammatory factors TNF-α, IL-6, IL-1β, COX-2 and CXCL1 content in rats. *P < 0.05, **P < 0.01.

We have demonstrated PLK2 is required for HDG mediated cytotoxicity and inflammatory responses *in vitro*, we next explored whether these changes occur *in vivo*. We introduced shPLK2 or shRNA control into the diabetic rats via intravenous tail injection. To investigate the renal injury induced by diabetes in rats, we first measured the urinary levels of creatinine, nitrogen and protein. The contents of urinary creatinine, nitrogen and protein were significant increase in diabetic rats, and shPLK2 inhibited these changes (Fig. 8A–C). As shown in Fig. 8D–F, diabetic rats showed increased expression of PLK2 and TUNEL-positive cells in glomeruli. Knocking down PLK2 restored the glomeruli morphology and reduced apoptosis (Fig. 8D). Interestingly, diabetic rats exhibited disorganized glomeruli (Nephrin staining), which was restored by knocking down PLK2 (Fig. 8D). shRNA control did not exhibit obvious changes compared to control rats (data not shown). Consistent with this, changes in apoptosis were prevented after suppressing PLK2 (Fig. 8E and F). Inflammatory factors in peripheral blood, including TNF-α, IL-6, IL-1β, COX-2 and CXCL1, were increased in diabetic rats. PLK2 knockdown significantly suppressed the production these inflammatory factors (Fig. 8G). These findings demonstrated that our *in vitro* data was successfully recapitulated *in vivo*, indicating that PLK2 promotes apoptosis and inflammatory responses in diabetes progression *in vivo*.

## Discussion

The mortality of nephropathy in diabetic patients increases dramatically in the recent years. DKD is the leading cause of the primary end-stage renal disease. Although attempts to understand DKD underlying mechanisms are ongoing, genes that contribute to DKD development and progression are not fully elucidated.

Microarray analysis for gene expression in DKD was performed recently^22,33^. More than 1700 genes are involved in DKD development^22^. The advance in microarray has led to a number of novel findings in kidney research, for example, Thiol genes are up-regulated by high glucose to buffer oxidative stress^34^. The other example is OSMRβ, its upregulation in renal epithelial cells is associated with myofibroblast differentiation^35^. However, microarray highly relies on existing gene patterns and it is insensitive to distinguish similar sequences in different genes^23,33^.

RNA-sequencing (RNA-seq) offers substantially enhanced sensitivity in detecting differentially expressed genes when compared with microarrays and is widely used in recently years^36–38^. Using RNA-seq, we identified 340 out of 16880 transcripts exhibited distinct expression patterns between control and DKD tissues. Majority of the transcripts (214) were decreased in DKD glomeruli, including *Scd*1, *Crygb*, *Ifit1*, and *Pbk*. On the other hand, transcripts showed the highest increase were *Dmrtclc*, *Kif5c*, *RT1-Ba*, and *Grem2*. Some genes such as *Grem2*, a BMP antagonist, have been identified previously with similar changes^19,39^. However, *Crygb* in DKD glomeruli of diabetic rats was decreased. These results are opposite to microarray data that Crygb in retinal cells in diabetic rats were upregulated^40^.

Here we also identified 22 signaling pathways in diabetic rats, some of were reported for the first time. Toll-like receptor signaling and Tight-junction signaling were consistent with previous work^22^. We discovered the significant changes in PLK2 in DKD rat models, which were confirmed by GSE30122 database. Our results demonstrated that PLK2 high expression is correlated with cell cycle progression, p53 signaling and apoptosis. Whereas PLK2 low expression is correlated with oxidative phosphorylation, mTOR signaling, and JAK/STAT signaling.

We further investigated PLK2 biological function by manipulating its expression *in vitro* and *in vivo*. We found that HDG treatment triggers podocyte apoptosis and ROS generation, which is consistent with previous work that HDG stimulated rapid ROS generation in mouse podocytes from mitochondrial sources^3,41–43^. It is known that several inflammatory cytokines contribute to DKD pathogenesis, such as TNF-α, IL-6, IL-1β, COX-2, and CXCL1, which are increased in DKD patient serum^44,45^. We found that HDG increases ROS levels and induces apoptosis in mouse podocytes. Consistent with this, NAC, an anti-oxidant reagent, efficiently inhibits HDG-induced apoptosis, increased ROS and inflammatory responses. Overexpressing PLK2 reversed NAC effects, suggesting that the crosstalk between NAC and PLK2-mediated responses^21,46^. However, treatment with DM and HLG did not affect the viability, cell cycle, apoptosis, ROS production, MMP levels and inflammatory responses of mouse podocytes.

Considering the p53 and apoptosis pathway were involved in PLK2-dependent DKD progression, the expression of SIRT5, Bcl-2, Bax, p53 and caspase-3 was also detected in HDG-induced mouse podocytes. SIRT5 is one of factors that are involved in apoptosis^47^. Mortuza *et al*. identified SIRT5 is decreased in HDG-induced cells^48^. SIRT5 expression changes in HDG-induced mouse podocytes^49,50^. Decreased ratio of Bcl-2/Bax damaged the integrity of mitochondria and led to the activation of caspase-3 in HDG-induced apoptosis in mouse podocytes. PLK2 mediates apoptosis through p53, because the antioxidant activity of PLK2 is the key factor to prevent p53-dependent cell death in neurodegenerative diseases and cancer^21^. Strikingly, we confirmed our *in vitro* results using streptozotocin (STZ)-induced diabetic rats. Decreased ratio of Bcl-2/Bax and increased cleaved caspase-3 and p53 expression were previously reported in STZ-induced diabetic rats^51,52^. Whereas others found that insistency of Bax/Bcl-2 and cleaved caspase-3 changes in *db/db* rats^53^.

We recognize several limitations to the present study which need to be considered when interpreting these results. The diabetic rat and human disease dataset collected in this study is limited with respect to sample size and clinical parameter information. Furthermore, it is noteworthy that as yet no RNA-seq dataset is available from *in vivo* model of DKD rat, notwithstanding the controversies about such models. Finally, other signaling such as JAK/STAT, Oxidative phosphorylation, and Calcium signaling pathways need further investigation.

In summary, our study provided a complete set of gene profile in diabetic rats.Our results on PLK2 shed light on future therapeutic target in DKD progression.

## Electronic supplementary material

Supplementary information
