# Author Correction: PLK2 Plays an Essential Role in High D-Glucose-Induced Apoptosis, ROS Generation and Inflammation in Podocytes

**DOI:** 10.1038/s41598-018-21278-0

**Published:** 2018-03-06

**Authors:** Hong-hong Zou, Ping-ping Yang, Tian-lun Huang, Xiao-xu Zheng, Gao-si Xu

**Affiliations:** 1 Department of Nephrology, the Second Affiliated Hospital of Nanchang University, No. 1 Minde Road, Nanchang, 330006 P.R. China; 2 Department of Medicine, the George Washington University, Washington, DC20052 USA

Correction to: *Scientific Reports* 10.1038/s41598-017-00686-8, published online 27 June 2017

In the original version of this Article, affiliation 2 was inadvertently included. The correct affiliations are listed below:

Department of Nephrology, the Second Affiliated Hospital of Nanchang University, No. 1 Minde Road, Nanchang, 330006, P.R. China.

Hong-hong Zou, Ping-ping Yang, Tian-lun Huang & Gao-si Xu

Department of Medicine, the George Washington University, Washington, DC20052, USA.

Xiao-xu Zheng

This error has now been corrected in the PDF and HTML versions of the Article.

