# Supplementary material for: PLK2 Plays an Essential Role in High D-Glucose-Induced Apoptosis, ROS Generation and Inflammation in Podocytes
Source: Sci Rep. 2017 Jun 27;7:4261. doi: 10.1038/s41598-017-00686-8 (PMC5487358; doi:10.1038/s41598-017-00686-8)
Supplement: Supplementary file 1 — Supplementary information [file 41598_2017_686_MOESM1_ESM.doc]

**PLK2 Plays an Essential Role in High D-Glucose-Induced Apoptosis, ROS Generation and Inflammation in Podocytes**

**Hong-hong Zou, Ping-ping Yang, Tian-lun Huang, Xiao-xu Zheng, and Gao-si Xu**

**Supplementary Table 1.** Selected differentially expressed genes in diabetes-induced DKD rats glomeruli

| Gene | Sample-1 | Sample-2 | Value-1 | Value-2 | log_2_ (fold change) | | *P*-value | q_value |
| --- | --- | --- | --- | --- | --- | --- | --- | --- |
| *Dmrtc1c* | Normal | DKD | 6.94E-01 | 1.25E+02 | | 7.49E+00 | 5.00E-05 | 6.70E-04 |
| *Kif5c* | Normal | DKD | 1.04E-01 | 7.11E+00 | | 6.09E+00 | 5.00E-05 | 6.70E-04 |
| *RT1-Ba* | Normal | DKD | 6.71E-01 | 3.76E+01 | | 5.81E+00 | 5.00E-05 | 6.70E-04 |
| *Grem2* | Normal | DKD | 7.73E-01 | 1.77E+01 | | 4.52E+00 | 5.00E-05 | 6.70E-04 |
| *Upk2* | Normal | DKD | 1.86E-01 | 4.06E+00 | | 4.45E+00 | 2.60E-03 | 1.81E-02 |
| *Akr1b8* | Normal | DKD | 2.89E+00 | 5.53E+01 | | 4.26E+00 | 5.00E-05 | 6.70E-04 |
| *Anxa8* | Normal | DKD | 6.73E-02 | 1.14E+00 | | 4.08E+00 | 2.60E-03 | 1.81E-02 |
| *P2rx5* | Normal | DKD | 1.37E-01 | 2.14E+00 | | 3.97E+00 | 2.50E-04 | 2.72E-03 |
| *Dpep3* | Normal | DKD | 1.65E-01 | 2.47E+00 | | 3.90E+00 | 2.70E-03 | 1.86E-02 |
| *Lypd2* | Normal | DKD | 2.28E+00 | 3.32E+01 | | 3.86E+00 | 5.00E-05 | 6.70E-04 |
| *RT1-CE10* | Normal | DKD | 1.98E+00 | 2.65E+01 | | 3.75E+00 | 5.00E-05 | 6.70E-04 |
| *Ephx2* | Normal | DKD | 7.50E+00 | 9.66E+01 | | 3.69E+00 | 5.00E-05 | 6.70E-04 |
| *Epor* | Normal | DKD | 1.77E-01 | 2.27E+00 | | 3.68E+00 | 1.80E-03 | 1.34E-02 |
| *Emilin3* | Normal | DKD | 9.36E-02 | 1.17E+00 | | 3.64E+00 | 1.30E-03 | 1.03E-02 |
| *Ceacam20* | Normal | DKD | 6.30E-01 | 7.20E+00 | | 3.51E+00 | 5.00E-05 | 6.70E-04 |
| *Mis18a* | Normal | DKD | 4.93E-01 | 5.48E+00 | | 3.47E+00 | 1.00E-04 | 1.25E-03 |
| *Robo3* | Normal | DKD | 4.20E-01 | 4.47E+00 | | 3.41E+00 | 5.00E-05 | 6.70E-04 |
| *Fndc1* | Normal | DKD | 5.87E-01 | 5.91E+00 | | 3.33E+00 | 5.00E-05 | 6.70E-04 |
| *Sprr1a* | Normal | DKD | 5.81E-01 | 5.50E+00 | | 3.24E+00 | 2.65E-03 | 1.83E-02 |
| *Msln* | Normal | DKD | 1.68E-01 | 1.42E+00 | | 3.09E+00 | 1.90E-03 | 1.40E-02 |
| *Slco4a1* | Normal | DKD | 2.03E+00 | 1.68E+01 | | 3.05E+00 | 5.00E-05 | 6.70E-04 |
| *Pcsk9* | Normal | DKD | 2.73E-01 | 1.98E+00 | | 2.86E+00 | 5.00E-05 | 6.70E-04 |
| *Dnase1* | Normal | DKD | 3.03E+02 | 2.19E+03 | | 2.85E+00 | 5.00E-05 | 6.70E-04 |
| *Gjb5* | Normal | DKD | 4.35E-01 | 3.11E+00 | | 2.84E+00 | 7.60E-03 | 4.09E-02 |
| *Kng1* | Normal | DKD | 2.29E+00 | 1.61E+01 | | 2.81E+00 | 5.00E-05 | 6.70E-04 |
| *Tnfsf15* | Normal | DKD | 5.92E-01 | 4.10E+00 | | 2.79E+00 | 2.50E-03 | 1.75E-02 |
| *Upk1b* | Normal | DKD | 2.32E-01 | 1.52E+00 | | 2.71E+00 | 1.55E-03 | 1.19E-02 |
| *Lamb3* | Normal | DKD | 5.61E-01 | 3.63E+00 | | 2.69E+00 | 5.00E-05 | 6.70E-04 |
| *RT1-A1,RT1-A3* | Normal | DKD | 9.47E+00 | 5.97E+01 | | 2.66E+00 | 3.00E-04 | 3.14E-03 |
| *Ambp* | Normal | DKD | 5.41E+00 | 3.30E+01 | | 2.61E+00 | 5.00E-05 | 6.70E-04 |
| *Bcat1* | Normal | DKD | 4.74E-01 | 2.81E+00 | | 2.57E+00 | 5.00E-05 | 6.70E-04 |
| *Adamtsl5* | Normal | DKD | 5.81E-01 | 3.39E+00 | | 2.54E+00 | 9.00E-04 | 7.68E-03 |
| *Begain* | Normal | DKD | 2.05E-01 | 1.19E+00 | | 2.53E+00 | 2.00E-04 | 2.27E-03 |
| *Zbtb16* | Normal | DKD | 3.57E-01 | 2.05E+00 | | 2.52E+00 | 5.00E-05 | 6.70E-04 |
| *Etv4* | Normal | DKD | 3.00E-01 | 1.68E+00 | | 2.49E+00 | 1.00E-04 | 1.25E-03 |
| *Map3k6* | Normal | DKD | 2.52E-01 | 1.41E+00 | | 2.49E+00 | 5.00E-05 | 6.70E-04 |
| *Sbsn* | Normal | DKD | 6.04E-01 | 3.31E+00 | | 2.46E+00 | 4.00E-04 | 4.02E-03 |
| *Areg* | Normal | DKD | 1.67E+00 | 8.98E+00 | | 2.43E+00 | 5.00E-05 | 6.70E-04 |
| *Actg2* | Normal | DKD | 7.50E-01 | 4.02E+00 | | 2.42E+00 | 1.00E-04 | 1.25E-03 |
| *Sptssb* | Normal | DKD | 3.46E-01 | 1.82E+00 | | 2.40E+00 | 1.00E-04 | 1.25E-03 |
| *Cml2* | Normal | DKD | 1.25E+00 | 6.59E+00 | | 2.39E+00 | 1.00E-03 | 8.31E-03 |
| *Psca* | Normal | DKD | 8.20E+00 | 4.31E+01 | | 2.39E+00 | 5.00E-05 | 6.70E-04 |
| *Fzd9* | Normal | DKD | 3.80E-01 | 1.99E+00 | | 2.39E+00 | 5.00E-05 | 6.70E-04 |
| *Gcom1* | Normal | DKD | 2.52E+00 | 1.32E+01 | | 2.39E+00 | 5.00E-05 | 6.70E-04 |
| *Sphkap* | Normal | DKD | 2.14E-01 | 1.12E+00 | | 2.39E+00 | 5.00E-05 | 6.70E-04 |
| *Cldn23* | Normal | DKD | 1.27E+00 | 6.56E+00 | | 2.37E+00 | 5.00E-05 | 6.70E-04 |
| *Per1* | Normal | DKD | 7.00E+00 | 3.46E+01 | | 2.31E+00 | 5.00E-05 | 6.70E-04 |
| *Vash2* | Normal | DKD | 1.21E+00 | 5.96E+00 | | 2.30E+00 | 5.00E-05 | 6.70E-04 |
| *Nupr1* | Normal | DKD | 3.42E+01 | 1.67E+02 | | 2.28E+00 | 5.00E-05 | 6.70E-04 |
| *Uchl1* | Normal | DKD | 1.29E+00 | 6.29E+00 | | 2.28E+00 | 5.00E-05 | 6.70E-04 |
| *Slc47a2* | Normal | DKD | 2.66E-01 | 1.29E+00 | | 2.28E+00 | 4.45E-03 | 2.73E-02 |
| *Nrip3* | Normal | DKD | 1.61E+00 | 7.69E+00 | | 2.25E+00 | 5.00E-05 | 6.70E-04 |
| *Cdhr1* | Normal | DKD | 2.73E-01 | 1.30E+00 | | 2.24E+00 | 5.00E-05 | 6.70E-04 |
| *Cry1* | Normal | DKD | 2.39E+00 | 1.08E+01 | | 2.18E+00 | 5.00E-05 | 6.70E-04 |
| *Camkv* | Normal | DKD | 6.42E-01 | 2.90E+00 | | 2.18E+00 | 5.00E-05 | 6.70E-04 |
| *Rasal1* | Normal | DKD | 4.66E-01 | 2.09E+00 | | 2.17E+00 | 5.00E-05 | 6.70E-04 |
| *Pappa* | Normal | DKD | 1.92E+00 | 8.58E+00 | | 2.16E+00 | 5.00E-05 | 6.70E-04 |
| *Gpat2* | Normal | DKD | 3.28E-01 | 1.46E+00 | | 2.16E+00 | 2.00E-04 | 2.27E-03 |
| *Cyp24a1* | Normal | DKD | 4.34E+01 | 1.93E+02 | | 2.16E+00 | 5.00E-05 | 6.70E-04 |
| *Neu2* | Normal | DKD | 9.07E-01 | 3.97E+00 | | 2.13E+00 | 5.00E-05 | 6.70E-04 |
| *Itk* | Normal | DKD | 5.61E-01 | 2.43E+00 | | 2.11E+00 | 5.00E-05 | 6.70E-04 |
| *Ucma* | Normal | DKD | 7.42E-01 | 3.14E+00 | | 2.08E+00 | 4.25E-03 | 2.63E-02 |
| *Mycbpap* | Normal | DKD | 3.99E+00 | 1.68E+01 | | 2.08E+00 | 5.00E-05 | 6.70E-04 |
| *RGD1311723* | Normal | DKD | 1.65E+00 | 6.84E+00 | | 2.05E+00 | 1.00E-04 | 1.25E-03 |
| *Apoc2* | Normal | DKD | 4.39E+00 | 1.81E+01 | | 2.04E+00 | 5.00E-05 | 6.70E-04 |
| *Apba2* | Normal | DKD | 3.47E-01 | 1.43E+00 | | 2.04E+00 | 3.00E-04 | 3.14E-03 |
| *Acsm5* | Normal | DKD | 3.38E+00 | 1.39E+01 | | 2.04E+00 | 5.00E-05 | 6.70E-04 |
| *Akr1b1* | Normal | DKD | 2.78E+01 | 1.12E+02 | | 2.00E+00 | 5.00E-05 | 6.70E-04 |
| *Usp2* | Normal | DKD | 6.31E+00 | 2.50E+01 | | 1.99E+00 | 5.00E-05 | 6.70E-04 |
| *Slc16a3* | Normal | DKD | 1.85E+00 | 7.30E+00 | | 1.98E+00 | 5.00E-05 | 6.70E-04 |
| *Fstl3* | Normal | DKD | 7.95E-01 | 3.07E+00 | | 1.95E+00 | 5.10E-03 | 3.02E-02 |
| *Hipk4* | Normal | DKD | 4.75E-01 | 1.84E+00 | | 1.95E+00 | 5.00E-05 | 6.70E-04 |
| *Adra1b* | Normal | DKD | 1.77E+00 | 6.83E+00 | | 1.95E+00 | 5.00E-05 | 6.70E-04 |
| *Slc14a1* | Normal | DKD | 3.24E+00 | 1.23E+01 | | 1.92E+00 | 5.00E-05 | 6.70E-04 |
| *Npy1r* | Normal | DKD | 2.95E-01 | 1.10E+00 | | 1.91E+00 | 3.40E-03 | 2.21E-02 |
| *Clcnka* | Normal | DKD | 1.69E+01 | 6.32E+01 | | 1.90E+00 | 5.00E-05 | 6.70E-04 |
| *Lpin2* | Normal | DKD | 1.24E+01 | 4.64E+01 | | 1.90E+00 | 5.00E-05 | 6.70E-04 |
| *Maff* | Normal | DKD | 3.26E+00 | 1.21E+01 | | 1.89E+00 | 5.00E-05 | 6.70E-04 |
| *Eras* | Normal | DKD | 4.91E-01 | 1.82E+00 | | 1.89E+00 | 4.80E-03 | 2.89E-02 |
| *Bdkrb2* | Normal | DKD | 7.14E-01 | 2.62E+00 | | 1.88E+00 | 5.00E-05 | 6.70E-04 |
| *Sgk1* | Normal | DKD | 7.36E+01 | 2.66E+02 | | 1.86E+00 | 5.00E-05 | 6.70E-04 |
| *Col8a1* | Normal | DKD | 4.78E-01 | 1.73E+00 | | 1.85E+00 | 1.50E-04 | 1.77E-03 |
| *Ten1* | Normal | DKD | 1.24E+01 | 4.46E+01 | | 1.85E+00 | 5.00E-05 | 6.70E-04 |
| *Mcoln3* | Normal | DKD | 2.21E+01 | 7.92E+01 | | 1.84E+00 | 5.00E-05 | 6.70E-04 |
| *Aqp2* | Normal | DKD | 2.76E+02 | 9.84E+02 | | 1.84E+00 | 5.00E-05 | 6.70E-04 |
| *Sec14l1* | Normal | DKD | 2.55E+01 | 9.09E+01 | | 1.83E+00 | 5.00E-05 | 6.70E-04 |
| *Lingo4* | Normal | DKD | 3.22E+00 | 1.13E+01 | | 1.82E+00 | 5.00E-05 | 6.70E-04 |
| *Upk3bl* | Normal | DKD | 2.19E+00 | 7.66E+00 | | 1.81E+00 | 5.00E-05 | 6.70E-04 |
| *Ggt6* | Normal | DKD | 1.11E+00 | 3.85E+00 | | 1.80E+00 | 5.00E-05 | 6.70E-04 |
| *Capg* | Normal | DKD | 6.74E+00 | 2.34E+01 | | 1.80E+00 | 5.00E-05 | 6.70E-04 |
| *Esrrb* | Normal | DKD | 5.84E+00 | 2.03E+01 | | 1.79E+00 | 5.00E-05 | 6.70E-04 |
| *Dnm1* | Normal | DKD | 5.47E+00 | 1.89E+01 | | 1.79E+00 | 5.00E-05 | 6.70E-04 |
| *Lmod1* | Normal | DKD | 5.95E-01 | 2.01E+00 | | 1.76E+00 | 5.00E-05 | 6.70E-04 |
| *Slc10a6* | Normal | DKD | 6.68E-01 | 2.25E+00 | | 1.75E+00 | 7.65E-03 | 4.12E-02 |
| *RT1-A2* | Normal | DKD | 4.74E+01 | 1.59E+02 | | 1.75E+00 | 5.00E-05 | 6.70E-04 |
| *Tsc22d3* | Normal | DKD | 3.01E+01 | 1.01E+02 | | 1.74E+00 | 5.00E-05 | 6.70E-04 |
| *Pdlim3* | Normal | DKD | 4.63E-01 | 1.55E+00 | | 1.74E+00 | 2.70E-03 | 1.86E-02 |
| *Fetub* | Normal | DKD | 7.77E+00 | 2.57E+01 | | 1.73E+00 | 5.00E-05 | 6.70E-04 |
| *LOC500300* | Normal | DKD | 8.33E+00 | 2.75E+01 | | 1.72E+00 | 5.00E-05 | 6.70E-04 |
| *Tacstd2* | Normal | DKD | 4.84E+00 | 1.58E+01 | | 1.71E+00 | 5.00E-05 | 6.70E-04 |
| *Hjurp* | Normal | DKD | 1.37E+00 | 4.46E+00 | | 1.71E+00 | 5.00E-05 | 6.70E-04 |
| *Per2* | Normal | DKD | 1.02E+01 | 3.33E+01 | | 1.70E+00 | 5.00E-05 | 6.70E-04 |
| *Crtac1* | Normal | DKD | 6.46E-01 | 2.10E+00 | | 1.70E+00 | 5.00E-05 | 6.70E-04 |
| *Ly6al* | Normal | DKD | 1.35E+01 | 4.38E+01 | | 1.70E+00 | 5.00E-05 | 6.70E-04 |
| *Sptbn2* | Normal | DKD | 7.88E+00 | 2.53E+01 | | 1.68E+00 | 5.00E-05 | 6.70E-04 |
| *Angptl4* | Normal | DKD | 1.69E+00 | 5.37E+00 | | 1.67E+00 | 5.00E-05 | 6.70E-04 |
| *Prss53* | Normal | DKD | 4.42E+00 | 1.40E+01 | | 1.67E+00 | 5.00E-05 | 6.70E-04 |
| *Crabp2* | Normal | DKD | 7.19E-01 | 2.27E+00 | | 1.66E+00 | 6.40E-03 | 3.58E-02 |
| *Ccl9* | Normal | DKD | 4.87E-01 | 1.53E+00 | | 1.65E+00 | 8.90E-03 | 4.63E-02 |
| *Slc5a10* | Normal | DKD | 7.50E+01 | 2.35E+02 | | 1.65E+00 | 5.00E-05 | 6.70E-04 |
| *Sik1* | Normal | DKD | 3.29E+00 | 1.03E+01 | | 1.64E+00 | 5.00E-05 | 6.70E-04 |
| *Col27a1* | Normal | DKD | 1.70E+01 | 5.32E+01 | | 1.64E+00 | 5.00E-05 | 6.70E-04 |
| *Slc16a5* | Normal | DKD | 7.08E+00 | 2.20E+01 | | 1.64E+00 | 5.00E-05 | 6.70E-04 |
| *Rbm20* | Normal | DKD | 1.50E+00 | 4.67E+00 | | 1.64E+00 | 5.00E-05 | 6.70E-04 |
| *Irs3* | Normal | DKD | 1.08E+00 | 3.34E+00 | | 1.63E+00 | 1.00E-04 | 1.25E-03 |
| *Cnn1* | Normal | DKD | 1.54E+00 | 4.75E+00 | | 1.62E+00 | 5.00E-05 | 6.70E-04 |
| *Trim50* | Normal | DKD | 9.22E-01 | 2.83E+00 | | 1.62E+00 | 1.15E-03 | 9.30E-03 |
| *Krt10* | Normal | DKD | 1.08E+00 | 3.27E+00 | | 1.60E+00 | 5.00E-05 | 6.70E-04 |
| *Ptgs1* | Normal | DKD | 4.77E+00 | 1.43E+01 | | 1.58E+00 | 5.00E-05 | 6.70E-04 |
| *Ptges3l1* | Normal | DKD | 5.84E-01 | 1.74E+00 | | 1.58E+00 | 4.35E-03 | 2.68E-02 |
| *Rdh7* | Normal | DKD | 7.33E-01 | 2.19E+00 | | 1.58E+00 | 4.95E-03 | 2.95E-02 |
| *Slc2a3* | Normal | DKD | 5.27E-01 | 1.56E+00 | | 1.56E+00 | 9.05E-03 | 4.69E-02 |
| *Il12rb1* | Normal | DKD | 1.01E+00 | 2.98E+00 | | 1.56E+00 | 1.50E-04 | 1.77E-03 |
| *Hspb7* | Normal | DKD | 1.38E+00 | 4.04E+00 | | 1.56E+00 | 5.00E-05 | 6.70E-04 |
| *LOC498063* | Normal | DKD | 4.85E-01 | 1.37E+00 | | 1.50E+00 | 5.50E-04 | 5.22E-03 |
| *Pxmp4* | Normal | DKD | 7.92E+01 | 2.79E+01 | | -1.50E+00 | 5.00E-05 | 6.70E-04 |
| *Slamf8* | Normal | DKD | 2.67E+00 | 9.42E-01 | | -1.50E+00 | 6.00E-04 | 5.58E-03 |
| *Gabrp* | Normal | DKD | 1.31E+01 | 4.62E+00 | | -1.50E+00 | 5.00E-05 | 6.70E-04 |
| *Lims2* | Normal | DKD | 5.27E+01 | 1.85E+01 | | -1.51E+00 | 5.00E-05 | 6.70E-04 |
| *Rbp1* | Normal | DKD | 4.63E+02 | 1.62E+02 | | -1.51E+00 | 5.00E-05 | 6.70E-04 |
| *Tmeff1* | Normal | DKD | 1.95E+00 | 6.83E-01 | | -1.51E+00 | 1.50E-04 | 1.77E-03 |
| *Proc* | Normal | DKD | 5.64E+01 | 1.97E+01 | | -1.51E+00 | 5.00E-05 | 6.70E-04 |
| *Basp1* | Normal | DKD | 7.22E+01 | 2.51E+01 | | -1.52E+00 | 5.00E-05 | 6.70E-04 |
| *Iqcg* | Normal | DKD | 4.75E+00 | 1.65E+00 | | -1.53E+00 | 5.00E-05 | 6.70E-04 |
| *Ces2g* | Normal | DKD | 7.13E+01 | 2.46E+01 | | -1.53E+00 | 5.00E-05 | 6.70E-04 |
| *Ddc* | Normal | DKD | 3.46E+01 | 1.19E+01 | | -1.54E+00 | 5.00E-05 | 6.70E-04 |
| *Tgfb2* | Normal | DKD | 4.62E+00 | 1.59E+00 | | -1.54E+00 | 5.00E-05 | 6.70E-04 |
| *Cyba* | Normal | DKD | 4.13E+02 | 1.42E+02 | | -1.54E+00 | 5.00E-05 | 6.70E-04 |
| *Parm1* | Normal | DKD | 6.06E+01 | 2.08E+01 | | -1.54E+00 | 5.00E-05 | 6.70E-04 |
| *Rxrg* | Normal | DKD | 1.35E+01 | 4.64E+00 | | -1.54E+00 | 5.00E-05 | 6.70E-04 |
| *Lgmn* | Normal | DKD | 1.10E+03 | 3.77E+02 | | -1.54E+00 | 5.00E-05 | 6.70E-04 |
| *Dpp7* | Normal | DKD | 2.00E+02 | 6.84E+01 | | -1.54E+00 | 5.00E-05 | 6.70E-04 |
| *Fgf13* | Normal | DKD | 7.51E+00 | 2.57E+00 | | -1.55E+00 | 5.00E-05 | 6.70E-04 |
| *Osr2* | Normal | DKD | 3.73E+00 | 1.28E+00 | | -1.55E+00 | 2.00E-04 | 2.27E-03 |
| *Ifi204* | Normal | DKD | 3.65E+00 | 1.24E+00 | | -1.56E+00 | 5.00E-05 | 6.70E-04 |
| *Prkar2b* | Normal | DKD | 9.07E+00 | 3.08E+00 | | -1.56E+00 | 5.00E-05 | 6.70E-04 |
| *Klrk1* | Normal | DKD | 6.11E+00 | 2.07E+00 | | -1.56E+00 | 5.00E-05 | 6.70E-04 |
| *Clec10a* | Normal | DKD | 9.57E+00 | 3.23E+00 | | -1.57E+00 | 5.00E-05 | 6.70E-04 |
| *Cxcl13* | Normal | DKD | 2.13E+01 | 7.14E+00 | | -1.58E+00 | 5.00E-05 | 6.70E-04 |
| *Tmem229a* | Normal | DKD | 2.76E+00 | 9.22E-01 | | -1.58E+00 | 5.00E-05 | 6.70E-04 |
| *Tifab* | Normal | DKD | 2.69E+00 | 8.91E-01 | | -1.59E+00 | 9.50E-04 | 8.00E-03 |
| *Cacng5* | Normal | DKD | 8.34E+01 | 2.74E+01 | | -1.61E+00 | 5.00E-05 | 6.70E-04 |
| *Kif22* | Normal | DKD | 3.74E+00 | 1.23E+00 | | -1.61E+00 | 5.00E-05 | 6.70E-04 |
| *Asb9* | Normal | DKD | 2.06E+01 | 6.73E+00 | | -1.62E+00 | 5.00E-05 | 6.70E-04 |
| *Ahcyl2* | Normal | DKD | 1.32E+02 | 4.28E+01 | | -1.62E+00 | 5.00E-05 | 6.70E-04 |
| *Cx3cr1* | Normal | DKD | 6.84E+00 | 2.22E+00 | | -1.62E+00 | 5.00E-05 | 6.70E-04 |
| *Als2cr12* | Normal | DKD | 4.52E+00 | 1.47E+00 | | -1.62E+00 | 5.00E-05 | 6.70E-04 |
| *Ace* | Normal | DKD | 1.04E+01 | 3.35E+00 | | -1.64E+00 | 5.00E-05 | 6.70E-04 |
| *Ccr5* | Normal | DKD | 5.22E+00 | 1.68E+00 | | -1.64E+00 | 5.00E-05 | 6.70E-04 |
| *Lrrn1* | Normal | DKD | 1.59E+00 | 5.10E-01 | | -1.64E+00 | 3.00E-04 | 3.14E-03 |
| *LOC689064* | Normal | DKD | 1.95E+01 | 6.21E+00 | | -1.65E+00 | 5.00E-05 | 6.70E-04 |
| *Slc15a1* | Normal | DKD | 1.52E+01 | 4.83E+00 | | -1.65E+00 | 5.00E-04 | 4.87E-03 |
| *Hormad1* | Normal | DKD | 1.54E+00 | 4.90E-01 | | -1.65E+00 | 1.05E-03 | 8.64E-03 |
| *Smlr1* | Normal | DKD | 5.91E+01 | 1.87E+01 | | -1.66E+00 | 5.00E-05 | 6.70E-04 |
| *Tnfaip8* | Normal | DKD | 2.00E+02 | 6.34E+01 | | -1.66E+00 | 5.00E-05 | 6.70E-04 |
| *H1f0* | Normal | DKD | 1.49E+02 | 4.68E+01 | | -1.68E+00 | 5.00E-05 | 6.70E-04 |
| *Rwdd2a* | Normal | DKD | 2.34E+00 | 7.32E-01 | | -1.68E+00 | 4.50E-04 | 4.45E-03 |
| *Slc30a3* | Normal | DKD | 6.13E+00 | 1.91E+00 | | -1.68E+00 | 5.00E-05 | 6.70E-04 |
| *Smok2a* | Normal | DKD | 3.25E+00 | 1.01E+00 | | -1.68E+00 | 5.00E-05 | 6.70E-04 |
| *Acsf2* | Normal | DKD | 4.80E+01 | 1.49E+01 | | -1.69E+00 | 5.00E-05 | 6.70E-04 |
| *Bard1* | Normal | DKD | 1.10E+00 | 3.40E-01 | | -1.69E+00 | 1.55E-03 | 1.19E-02 |
| *Slc6a19* | Normal | DKD | 1.00E+02 | 3.09E+01 | | -1.69E+00 | 5.00E-05 | 6.70E-04 |
| *Sytl3* | Normal | DKD | 3.59E+00 | 1.11E+00 | | -1.70E+00 | 5.00E-05 | 6.70E-04 |
| *Epsti1* | Normal | DKD | 3.67E+00 | 1.12E+00 | | -1.70E+00 | 5.00E-05 | 6.70E-04 |
| *Ptpn22* | Normal | DKD | 1.49E+00 | 4.55E-01 | | -1.71E+00 | 1.50E-04 | 1.77E-03 |
| *Bub1* | Normal | DKD | 2.00E+00 | 6.10E-01 | | -1.71E+00 | 5.00E-05 | 6.70E-04 |
| *Hist1h4b* | Normal | DKD | 6.03E+01 | 1.84E+01 | | -1.71E+00 | 5.00E-05 | 6.70E-04 |
| *Dlgap5* | Normal | DKD | 2.51E+00 | 7.64E-01 | | -1.72E+00 | 5.00E-05 | 6.70E-04 |
| *Mlph* | Normal | DKD | 8.89E+00 | 2.71E+00 | | -1.72E+00 | 5.00E-05 | 6.70E-04 |
| *Maob* | Normal | DKD | 7.44E+00 | 2.25E+00 | | -1.73E+00 | 5.00E-05 | 6.70E-04 |
| *RGD1559548* | Normal | DKD | 1.22E+00 | 3.69E-01 | | -1.73E+00 | 7.50E-04 | 6.68E-03 |
| *Tmem144* | Normal | DKD | 3.39E+01 | 1.02E+01 | | -1.73E+00 | 5.00E-05 | 6.70E-04 |
| *Aldh1b1* | Normal | DKD | 1.16E+01 | 3.47E+00 | | -1.74E+00 | 5.00E-05 | 6.70E-04 |
| *Spink1* | Normal | DKD | 2.79E+03 | 8.33E+02 | | -1.74E+00 | 5.00E-05 | 6.70E-04 |
| *Prss35* | Normal | DKD | 2.47E+00 | 7.38E-01 | | -1.74E+00 | 5.00E-05 | 6.70E-04 |
| *Ctdspl2* | Normal | DKD | 6.17E+00 | 1.82E+00 | | -1.76E+00 | 5.00E-05 | 6.70E-04 |
| *Flrt3* | Normal | DKD | 3.04E+00 | 8.94E-01 | | -1.77E+00 | 5.00E-05 | 6.70E-04 |
| *RGD1305733* | Normal | DKD | 6.69E+00 | 1.96E+00 | | -1.77E+00 | 5.00E-05 | 6.70E-04 |
| *Nox4* | Normal | DKD | 9.15E+01 | 2.67E+01 | | -1.78E+00 | 5.00E-05 | 6.70E-04 |
| *Epb41l3* | Normal | DKD | 2.93E+01 | 8.44E+00 | | -1.79E+00 | 5.00E-05 | 6.70E-04 |
| *Angpt1* | Normal | DKD | 9.45E+00 | 2.69E+00 | | -1.81E+00 | 5.00E-05 | 6.70E-04 |
| *Slc4a5* | Normal | DKD | 4.30E+00 | 1.22E+00 | | -1.82E+00 | 5.00E-05 | 6.70E-04 |
| *Car14* | Normal | DKD | 4.49E+01 | 1.27E+01 | | -1.82E+00 | 5.00E-05 | 6.70E-04 |
| *Sox11* | Normal | DKD | 1.05E+00 | 2.95E-01 | | -1.83E+00 | 5.75E-03 | 3.32E-02 |
| *Col9a1* | Normal | DKD | 3.06E+00 | 8.57E-01 | | -1.84E+00 | 5.00E-05 | 6.70E-04 |
| *RGD1562673* | Normal | DKD | 4.04E+00 | 1.13E+00 | | -1.84E+00 | 5.50E-04 | 5.22E-03 |
| *Ralyl* | Normal | DKD | 2.11E+00 | 5.87E-01 | | -1.84E+00 | 1.05E-03 | 8.64E-03 |
| *Asgr1* | Normal | DKD | 1.90E+01 | 5.26E+00 | | -1.86E+00 | 5.00E-05 | 6.70E-04 |
| *Cd28* | Normal | DKD | 3.96E+00 | 1.09E+00 | | -1.86E+00 | 5.00E-05 | 6.70E-04 |
| *Fads1* | Normal | DKD | 5.37E+01 | 1.48E+01 | | -1.86E+00 | 5.00E-05 | 6.70E-04 |
| *Ttc30a1,Ttc30b* | Normal | DKD | 1.45E+00 | 3.98E-01 | | -1.87E+00 | 5.00E-04 | 4.87E-03 |
| *Slit1* | Normal | DKD | 1.86E+00 | 5.10E-01 | | -1.87E+00 | 5.00E-05 | 6.70E-04 |
| *Phgdh* | Normal | DKD | 9.75E+01 | 2.64E+01 | | -1.89E+00 | 5.00E-05 | 6.70E-04 |
| *Kif23* | Normal | DKD | 1.85E+00 | 4.99E-01 | | -1.89E+00 | 5.00E-05 | 6.70E-04 |
| *Scd* | Normal | DKD | 1.20E+02 | 3.23E+01 | | -1.89E+00 | 5.00E-05 | 6.70E-04 |
| *Kcne1* | Normal | DKD | 1.89E+01 | 5.06E+00 | | -1.90E+00 | 5.00E-05 | 6.70E-04 |
| *Arhgap11a* | Normal | DKD | 2.52E+00 | 6.73E-01 | | -1.91E+00 | 5.00E-05 | 6.70E-04 |
| *Tusc5* | Normal | DKD | 1.62E+00 | 4.31E-01 | | -1.91E+00 | 5.00E-05 | 6.70E-04 |
| *Idh1* | Normal | DKD | 6.25E+02 | 1.64E+02 | | -1.93E+00 | 5.00E-05 | 6.70E-04 |
| *Cfd* | Normal | DKD | 1.68E+01 | 4.42E+00 | | -1.93E+00 | 5.00E-05 | 6.70E-04 |
| *Lhfpl2* | Normal | DKD | 7.66E+00 | 2.00E+00 | | -1.94E+00 | 5.00E-05 | 6.70E-04 |
| *Melk* | Normal | DKD | 1.40E+01 | 3.64E+00 | | -1.94E+00 | 5.00E-05 | 6.70E-04 |
| *Irf4* | Normal | DKD | 1.99E+00 | 5.14E-01 | | -1.95E+00 | 5.00E-05 | 6.70E-04 |
| *Plk1* | Normal | DKD | 1.62E+00 | 4.18E-01 | | -1.96E+00 | 5.50E-04 | 5.22E-03 |
| *Fam151a* | Normal | DKD | 1.33E+02 | 3.43E+01 | | -1.96E+00 | 5.00E-05 | 6.70E-04 |
| *Gpx3* | Normal | DKD | 2.12E+04 | 5.44E+03 | | -1.96E+00 | 5.00E-05 | 6.70E-04 |
| *Cd7* | Normal | DKD | 6.09E+00 | 1.56E+00 | | -1.97E+00 | 1.00E-04 | 1.25E-03 |
| *Bcl2a1* | Normal | DKD | 1.20E+01 | 3.06E+00 | | -1.97E+00 | 5.00E-05 | 6.70E-04 |
| *Dhcr24* | Normal | DKD | 8.79E+01 | 2.23E+01 | | -1.98E+00 | 5.00E-05 | 6.70E-04 |
| *C1qtnf3* | Normal | DKD | 5.29E+01 | 1.34E+01 | | -1.98E+00 | 5.00E-05 | 6.70E-04 |
| *Serpinb12* | Normal | DKD | 2.19E+00 | 5.54E-01 | | -1.98E+00 | 5.00E-05 | 6.70E-04 |
| *LOC287167* | Normal | DKD | 1.32E+01 | 3.34E+00 | | -1.99E+00 | 5.50E-04 | 5.22E-03 |
| *Fos* | Normal | DKD | 1.18E+01 | 2.99E+00 | | -1.99E+00 | 5.00E-05 | 6.70E-04 |
| *Fancd2* | Normal | DKD | 2.61E+00 | 6.57E-01 | | -1.99E+00 | 5.00E-05 | 6.70E-04 |
| *Nabp1* | Normal | DKD | 9.12E+01 | 2.28E+01 | | -2.00E+00 | 5.00E-05 | 6.70E-04 |
| *Cyp2c11* | Normal | DKD | 2.58E+01 | 6.41E+00 | | -2.01E+00 | 5.00E-05 | 6.70E-04 |
| *Slc5a12* | Normal | DKD | 1.06E+02 | 2.63E+01 | | -2.02E+00 | 5.00E-05 | 6.70E-04 |
| *Gas2* | Normal | DKD | 2.44E+01 | 6.01E+00 | | -2.02E+00 | 5.00E-05 | 6.70E-04 |
| *Coch* | Normal | DKD | 4.67E+00 | 1.15E+00 | | -2.02E+00 | 3.45E-03 | 2.24E-02 |
| *Olr34* | Normal | DKD | 5.53E+00 | 1.35E+00 | | -2.04E+00 | 1.00E-04 | 1.25E-03 |
| *Sectm1* | Normal | DKD | 6.63E+00 | 1.61E+00 | | -2.04E+00 | 5.00E-05 | 6.70E-04 |
| *Gimd1* | Normal | DKD | 2.69E+00 | 6.53E-01 | | -2.04E+00 | 5.00E-05 | 6.70E-04 |
| *Cyp2d3* | Normal | DKD | 1.30E+00 | 3.13E-01 | | -2.05E+00 | 4.30E-03 | 2.65E-02 |
| *Arg1* | Normal | DKD | 2.24E+00 | 5.36E-01 | | -2.07E+00 | 3.00E-04 | 3.14E-03 |
| *Tpx2* | Normal | DKD | 2.84E+00 | 6.74E-01 | | -2.08E+00 | 5.00E-05 | 6.70E-04 |
| *Npl* | Normal | DKD | 2.41E+01 | 5.71E+00 | | -2.08E+00 | 5.00E-05 | 6.70E-04 |
| *Igf1* | Normal | DKD | 1.25E+01 | 2.93E+00 | | -2.09E+00 | 5.00E-05 | 6.70E-04 |
| *Cenpt* | Normal | DKD | 1.17E+00 | 2.75E-01 | | -2.09E+00 | 4.65E-03 | 2.82E-02 |
| *Hs6st2* | Normal | DKD | 1.30E+00 | 3.04E-01 | | -2.09E+00 | 5.00E-04 | 4.87E-03 |
| *Nrep* | Normal | DKD | 6.35E+01 | 1.48E+01 | | -2.10E+00 | 5.00E-05 | 6.70E-04 |
| *Rtp3* | Normal | DKD | 3.91E+00 | 9.13E-01 | | -2.10E+00 | 5.00E-05 | 6.70E-04 |
| *LOC100366030* | Normal | DKD | 2.31E+01 | 5.18E+00 | | -2.15E+00 | 1.40E-03 | 1.10E-02 |
| *Hbb-b1* | Normal | DKD | 1.11E+02 | 2.48E+01 | | -2.16E+00 | 3.60E-03 | 2.32E-02 |
| *Prrt2* | Normal | DKD | 2.07E+00 | 4.60E-01 | | -2.17E+00 | 1.50E-04 | 1.77E-03 |
| *Rgn* | Normal | DKD | 7.41E+02 | 1.65E+02 | | -2.17E+00 | 5.00E-05 | 6.70E-04 |
| *Slc16a2* | Normal | DKD | 1.95E+01 | 4.31E+00 | | -2.18E+00 | 5.00E-05 | 6.70E-04 |
| *Gc* | Normal | DKD | 6.78E+01 | 1.50E+01 | | -2.18E+00 | 5.00E-05 | 6.70E-04 |
| *Lilrb3l* | Normal | DKD | 1.14E+00 | 2.47E-01 | | -2.20E+00 | 7.50E-04 | 6.68E-03 |
| *Prkg2* | Normal | DKD | 1.20E+00 | 2.58E-01 | | -2.22E+00 | 5.00E-05 | 6.70E-04 |
| *Ces1e* | Normal | DKD | 5.15E+01 | 1.09E+01 | | -2.25E+00 | 5.00E-05 | 6.70E-04 |
| *Folh1* | Normal | DKD | 4.48E+01 | 9.30E+00 | | -2.27E+00 | 5.00E-05 | 6.70E-04 |
| *Galnt3* | Normal | DKD | 2.77E+01 | 5.63E+00 | | -2.30E+00 | 5.00E-05 | 6.70E-04 |
| *Nebl* | Normal | DKD | 2.82E+00 | 5.68E-01 | | -2.31E+00 | 5.00E-05 | 6.70E-04 |
| *Spc25* | Normal | DKD | 3.46E+00 | 6.92E-01 | | -2.32E+00 | 1.00E-04 | 1.25E-03 |
| *Tnfrsf9* | Normal | DKD | 7.79E+00 | 1.55E+00 | | -2.33E+00 | 5.00E-05 | 6.70E-04 |
| *Nuf2* | Normal | DKD | 2.27E+00 | 4.46E-01 | | -2.35E+00 | 5.00E-05 | 6.70E-04 |
| *Slc4a4* | Normal | DKD | 1.18E+02 | 2.30E+01 | | -2.36E+00 | 5.00E-05 | 6.70E-04 |
| *RGD1564894* | Normal | DKD | 1.07E+01 | 2.07E+00 | | -2.37E+00 | 5.00E-05 | 6.70E-04 |
| *Nmbr* | Normal | DKD | 2.67E+00 | 5.18E-01 | | -2.37E+00 | 1.00E-03 | 8.31E-03 |
| *Mapk4* | Normal | DKD | 1.72E+01 | 3.32E+00 | | -2.37E+00 | 5.00E-05 | 6.70E-04 |
| *Fam221a* | Normal | DKD | 2.56E+00 | 4.93E-01 | | -2.37E+00 | 1.00E-04 | 1.25E-03 |
| *Car3* | Normal | DKD | 4.67E+01 | 9.00E+00 | | -2.38E+00 | 5.00E-05 | 6.70E-04 |
| *Apln* | Normal | DKD | 3.38E+00 | 6.38E-01 | | -2.41E+00 | 5.00E-05 | 6.70E-04 |
| *Crb1* | Normal | DKD | 1.37E+00 | 2.57E-01 | | -2.42E+00 | 5.00E-05 | 6.70E-04 |
| *Dgkg* | Normal | DKD | 3.13E+01 | 5.86E+00 | | -2.42E+00 | 5.00E-05 | 6.70E-04 |
| *Grifin* | Normal | DKD | 4.78E+00 | 8.84E-01 | | -2.43E+00 | 6.25E-03 | 3.52E-02 |
| *Ccna2* | Normal | DKD | 4.08E+00 | 7.35E-01 | | -2.47E+00 | 5.00E-05 | 6.70E-04 |
| *Nr1d1* | Normal | DKD | 5.93E+01 | 1.07E+01 | | -2.48E+00 | 5.00E-05 | 6.70E-04 |
| *Tmem86a* | Normal | DKD | 5.16E+01 | 9.27E+00 | | -2.48E+00 | 5.00E-05 | 6.70E-04 |
| *Pamr1* | Normal | DKD | 1.00E+01 | 1.78E+00 | | -2.49E+00 | 5.00E-05 | 6.70E-04 |
| *Plin1* | Normal | DKD | 6.32E+00 | 1.12E+00 | | -2.50E+00 | 5.00E-05 | 6.70E-04 |
| *Mapk10* | Normal | DKD | 1.95E+00 | 3.41E-01 | | -2.52E+00 | 5.00E-05 | 6.70E-04 |
| *Cilp* | Normal | DKD | 1.86E+00 | 3.20E-01 | | -2.54E+00 | 5.00E-05 | 6.70E-04 |
| *Krt19* | Normal | DKD | 4.06E+01 | 6.87E+00 | | -2.56E+00 | 5.00E-05 | 6.70E-04 |
| *Adrb3* | Normal | DKD | 1.32E+00 | 2.23E-01 | | -2.57E+00 | 2.60E-03 | 1.81E-02 |
| *Aurkb* | Normal | DKD | 2.44E+00 | 4.11E-01 | | -2.57E+00 | 5.00E-05 | 6.70E-04 |
| *Snap91* | Normal | DKD | 4.84E+00 | 8.15E-01 | | -2.57E+00 | 5.00E-05 | 6.70E-04 |
| *Prc1* | Normal | DKD | 3.67E+00 | 6.17E-01 | | -2.57E+00 | 5.00E-05 | 6.70E-04 |
| *Timm8a2* | Normal | DKD | 2.70E+00 | 4.49E-01 | | -2.59E+00 | 2.80E-03 | 1.91E-02 |
| *Hsd17b2* | Normal | DKD | 4.84E+00 | 7.98E-01 | | -2.60E+00 | 5.00E-05 | 6.70E-04 |
| *Ccr1* | Normal | DKD | 1.69E+00 | 2.78E-01 | | -2.60E+00 | 5.50E-04 | 5.22E-03 |
| *Ube2c* | Normal | DKD | 6.97E+00 | 1.14E+00 | | -2.61E+00 | 5.00E-05 | 6.70E-04 |
| *Nusap1* | Normal | DKD | 2.13E+00 | 3.49E-01 | | -2.61E+00 | 5.00E-05 | 6.70E-04 |
| *RGD1309350* | Normal | DKD | 8.51E+00 | 1.38E+00 | | -2.63E+00 | 5.00E-05 | 6.70E-04 |
| *Kifc1* | Normal | DKD | 3.22E+01 | 5.10E+00 | | -2.66E+00 | 5.00E-05 | 6.70E-04 |
| *Pdilt* | Normal | DKD | 1.58E+00 | 2.51E-01 | | -2.66E+00 | 3.00E-04 | 3.14E-03 |
| *Iqch* | Normal | DKD | 6.26E+00 | 9.90E-01 | | -2.66E+00 | 5.00E-05 | 6.70E-04 |
| *Npas2* | Normal | DKD | 5.98E+00 | 9.16E-01 | | -2.71E+00 | 5.00E-05 | 6.70E-04 |
| *Npy* | Normal | DKD | 1.41E+01 | 2.16E+00 | | -2.71E+00 | 5.00E-05 | 6.70E-04 |
| *Pttg1* | Normal | DKD | 3.90E+00 | 5.93E-01 | | -2.72E+00 | 4.50E-04 | 4.45E-03 |
| *Psph* | Normal | DKD | 1.08E+02 | 1.60E+01 | | -2.75E+00 | 5.00E-05 | 6.70E-04 |
| *Ccnb1* | Normal | DKD | 4.66E+00 | 6.87E-01 | | -2.76E+00 | 5.00E-05 | 6.70E-04 |
| *F13b* | Normal | DKD | 1.83E+01 | 2.69E+00 | | -2.77E+00 | 5.00E-05 | 6.70E-04 |
| *Slc7a7* | Normal | DKD | 1.56E+02 | 2.28E+01 | | -2.77E+00 | 5.00E-05 | 6.70E-04 |
| *Ccnb2* | Normal | DKD | 6.15E+00 | 8.90E-01 | | -2.79E+00 | 5.00E-05 | 6.70E-04 |
| *Enpp6* | Normal | DKD | 3.62E+01 | 5.18E+00 | | -2.81E+00 | 5.00E-05 | 6.70E-04 |
| *Cftr* | Normal | DKD | 3.34E+00 | 4.74E-01 | | -2.82E+00 | 5.00E-05 | 6.70E-04 |
| *Mki67* | Normal | DKD | 1.48E+00 | 2.09E-01 | | -2.82E+00 | 5.00E-05 | 6.70E-04 |
| *Hmmr* | Normal | DKD | 3.76E+00 | 5.07E-01 | | -2.89E+00 | 5.00E-05 | 6.70E-04 |
| *Troap* | Normal | DKD | 1.32E+00 | 1.77E-01 | | -2.90E+00 | 3.00E-04 | 3.14E-03 |
| *Gatm* | Normal | DKD | 2.73E+03 | 3.64E+02 | | -2.90E+00 | 5.00E-05 | 6.70E-04 |
| *Hsd17b1* | Normal | DKD | 1.63E+01 | 2.16E+00 | | -2.92E+00 | 5.00E-05 | 6.70E-04 |
| *Hao1* | Normal | DKD | 3.19E+00 | 4.06E-01 | | -2.97E+00 | 2.00E-04 | 2.27E-03 |
| *Slc16a1* | Normal | DKD | 3.66E+01 | 4.43E+00 | | -3.05E+00 | 5.00E-05 | 6.70E-04 |
| *Pnma2* | Normal | DKD | 2.44E+00 | 2.95E-01 | | -3.05E+00 | 5.00E-05 | 6.70E-04 |
| *Rpp25* | Normal | DKD | 2.10E+01 | 2.53E+00 | | -3.06E+00 | 5.00E-05 | 6.70E-04 |
| *RT1-N2* | Normal | DKD | 3.12E+01 | 3.67E+00 | | -3.09E+00 | 5.00E-05 | 6.70E-04 |
| *Fcrls* | Normal | DKD | 1.53E+00 | 1.67E-01 | | -3.20E+00 | 6.00E-04 | 5.58E-03 |
| *Inmt* | Normal | DKD | 2.56E+02 | 2.75E+01 | | -3.21E+00 | 5.00E-05 | 6.70E-04 |
| *Cdk1* | Normal | DKD | 7.85E+00 | 8.42E-01 | | -3.22E+00 | 5.00E-05 | 6.70E-04 |
| *Snca* | Normal | DKD | 1.32E+01 | 1.40E+00 | | -3.24E+00 | 5.00E-05 | 6.70E-04 |
| *Foxn4* | Normal | DKD | 1.07E+00 | 1.07E-01 | | -3.32E+00 | 2.50E-03 | 1.75E-02 |
| *Hnrnpa3* | Normal | DKD | 1.43E+02 | 1.39E+01 | | -3.36E+00 | 5.00E-05 | 6.70E-04 |
| *C6* | Normal | DKD | 3.40E+00 | 3.30E-01 | | -3.37E+00 | 5.00E-05 | 6.70E-04 |
| *Tlr12* | Normal | DKD | 2.46E+01 | 2.34E+00 | | -3.40E+00 | 5.00E-05 | 6.70E-04 |
| *Pcdh9* | Normal | DKD | 2.02E+00 | 1.90E-01 | | -3.41E+00 | 5.00E-05 | 6.70E-04 |
| *Ckap2* | Normal | DKD | 2.92E+00 | 2.68E-01 | | -3.45E+00 | 5.00E-05 | 6.70E-04 |
| *Retn* | Normal | DKD | 1.06E+01 | 9.59E-01 | | -3.46E+00 | 3.00E-04 | 3.14E-03 |
| *Tsx* | Normal | DKD | 1.66E+01 | 1.45E+00 | | -3.52E+00 | 5.00E-05 | 6.70E-04 |
| *Mmp9* | Normal | DKD | 6.82E+00 | 5.85E-01 | | -3.54E+00 | 5.00E-05 | 6.70E-04 |
| *LOC361914* | Normal | DKD | 3.46E+02 | 2.95E+01 | | -3.55E+00 | 5.00E-05 | 6.70E-04 |
| *Prima1* | Normal | DKD | 1.42E+01 | 1.15E+00 | | -3.62E+00 | 5.00E-05 | 6.70E-04 |
| *Egr1* | Normal | DKD | 5.92E+01 | 4.76E+00 | | -3.64E+00 | 5.00E-05 | 6.70E-04 |
| *Top2a* | Normal | DKD | 2.51E+00 | 1.99E-01 | | -3.66E+00 | 5.00E-05 | 6.70E-04 |
| *Slc7a12* | Normal | DKD | 2.57E+02 | 2.03E+01 | | -3.66E+00 | 5.00E-05 | 6.70E-04 |
| *Slc22a13* | Normal | DKD | 3.40E+01 | 2.67E+00 | | -3.67E+00 | 5.00E-05 | 6.70E-04 |
| *Ptgds* | Normal | DKD | 1.42E+02 | 1.10E+01 | | -3.68E+00 | 5.00E-05 | 6.70E-04 |
| *Acot5* | Normal | DKD | 3.63E+00 | 2.30E-01 | | -3.98E+00 | 1.50E-04 | 1.77E-03 |
| *Atp1a4* | Normal | DKD | 2.55E+00 | 1.42E-01 | | -4.16E+00 | 5.00E-05 | 6.70E-04 |
| *Tcerg1l* | Normal | DKD | 2.73E+00 | 1.51E-01 | | -4.17E+00 | 8.50E-04 | 7.37E-03 |
| *Cdca3* | Normal | DKD | 3.34E+00 | 1.53E-01 | | -4.45E+00 | 4.20E-03 | 2.61E-02 |
| *Apcs* | Normal | DKD | 3.52E+02 | 1.57E+01 | | -4.49E+00 | 5.00E-05 | 6.70E-04 |
| *Anxa13* | Normal | DKD | 2.38E+01 | 9.98E-01 | | -4.57E+00 | 5.00E-05 | 6.70E-04 |
| *Thrsp* | Normal | DKD | 5.75E+01 | 2.32E+00 | | -4.63E+00 | 5.00E-05 | 6.70E-04 |
| *Pbk* | Normal | DKD | 3.43E+00 | 1.30E-01 | | -4.72E+00 | 3.65E-03 | 2.33E-02 |
| *Ifit1* | Normal | DKD | 4.78E+00 | 1.30E-01 | | -5.21E+00 | 8.00E-04 | 7.01E-03 |
| *Crygb* | Normal | DKD | 1.35E+01 | 2.83E-01 | | -5.57E+00 | 2.65E-03 | 1.83E-02 |
| *Scd1* | Normal | DKD | 5.16E+01 | 2.03E-01 | | -7.99E+00 | 5.00E-05 | 6.70E-04 |

**Supplementary Table 2.** Selected enriched pathways in PLK2 high group in kidney tissues

| Pathway | NES | *P*-value | FDR q-value | Molecules |
| --- | --- | --- | --- | --- |
| Cell cycle | 2.28E+00 | 0.00E+00 | 1.14E-03 | [RB1](https://www.affymetrix.com/LinkServlet?probeset=RB1), [MAD2L1](https://www.affymetrix.com/LinkServlet?probeset=MAD2L1), [BUB3](https://www.affymetrix.com/LinkServlet?probeset=BUB3), [STAG1](https://www.affymetrix.com/LinkServlet?probeset=STAG1), [TGFB2](https://www.affymetrix.com/LinkServlet?probeset=TGFB2), [GSK3B](https://www.affymetrix.com/LinkServlet?probeset=GSK3B), [RBL2](https://www.affymetrix.com/LinkServlet?probeset=RBL2), [MYC](https://www.affymetrix.com/LinkServlet?probeset=MYC), [MCM3](https://www.affymetrix.com/LinkServlet?probeset=MCM3), [PCNA](https://www.affymetrix.com/LinkServlet?probeset=PCNA), [YWHAZ](https://www.affymetrix.com/LinkServlet?probeset=YWHAZ), [YWHAH](https://www.affymetrix.com/LinkServlet?probeset=YWHAH), [ATM](https://www.affymetrix.com/LinkServlet?probeset=ATM), [CCNB1](https://www.affymetrix.com/LinkServlet?probeset=CCNB1), [HDAC2](https://www.affymetrix.com/LinkServlet?probeset=HDAC2), [SMC3](https://www.affymetrix.com/LinkServlet?probeset=SMC3), [CCND2](https://www.affymetrix.com/LinkServlet?probeset=CCND2), [RBL1](https://www.affymetrix.com/LinkServlet?probeset=RBL1), [DBF4](https://www.affymetrix.com/LinkServlet?probeset=DBF4), [CCNA2](https://www.affymetrix.com/LinkServlet?probeset=CCNA2), [CCNE1](https://www.affymetrix.com/LinkServlet?probeset=CCNE1), [YWHAB](https://www.affymetrix.com/LinkServlet?probeset=YWHAB), [YWHAQ](https://www.affymetrix.com/LinkServlet?probeset=YWHAQ), [CCNB2](https://www.affymetrix.com/LinkServlet?probeset=CCNB2), [CDK1](https://www.affymetrix.com/LinkServlet?probeset=CDK1), [CHEK1](https://www.affymetrix.com/LinkServlet?probeset=CHEK1), [CDC25B](https://www.affymetrix.com/LinkServlet?probeset=CDC25B), [PRKDC](https://www.affymetrix.com/LinkServlet?probeset=PRKDC), [SMAD2](https://www.affymetrix.com/LinkServlet?probeset=SMAD2), [E2F5](https://www.affymetrix.com/LinkServlet?probeset=E2F5), [CCND1](https://www.affymetrix.com/LinkServlet?probeset=CCND1), [STAG2](https://www.affymetrix.com/LinkServlet?probeset=STAG2), [CDK7](https://www.affymetrix.com/LinkServlet?probeset=CDK7), [CDC16](https://www.affymetrix.com/LinkServlet?probeset=CDC16), [CHEK2](https://www.affymetrix.com/LinkServlet?probeset=CHEK2), [CDKN1A](https://www.affymetrix.com/LinkServlet?probeset=CDKN1A), [MCM7](https://www.affymetrix.com/LinkServlet?probeset=MCM7), [CDC20](https://www.affymetrix.com/LinkServlet?probeset=CDC20), [E2F3](https://www.affymetrix.com/LinkServlet?probeset=E2F3), [BUB1B](https://www.affymetrix.com/LinkServlet?probeset=BUB1B), [CUL1](https://www.affymetrix.com/LinkServlet?probeset=CUL1), [ORC5](https://www.affymetrix.com/LinkServlet?probeset=ORC5), [CDKN1B](https://www.affymetrix.com/LinkServlet?probeset=CDKN1B), [SMAD4](https://www.affymetrix.com/LinkServlet?probeset=SMAD4), [MDM2](https://www.affymetrix.com/LinkServlet?probeset=MDM2), [SMC1A](https://www.affymetrix.com/LinkServlet?probeset=SMC1A), [ATR](https://www.affymetrix.com/LinkServlet?probeset=ATR), and [CREBBP](https://www.affymetrix.com/LinkServlet?probeset=CREBBP) |
| p53 signaling pathway | 2.00E+00 | 0.00E+00 | 9.53E-03 | [CASP8](https://www.affymetrix.com/LinkServlet?probeset=CASP8), [PERP](https://www.affymetrix.com/LinkServlet?probeset=PERP), [FAS](https://www.affymetrix.com/LinkServlet?probeset=FAS), [APAF1](https://www.affymetrix.com/LinkServlet?probeset=APAF1), [RRM2](https://www.affymetrix.com/LinkServlet?probeset=RRM2), [CASP3](https://www.affymetrix.com/LinkServlet?probeset=CASP3), [THBS1](https://www.affymetrix.com/LinkServlet?probeset=THBS1), [ATM](https://www.affymetrix.com/LinkServlet?probeset=ATM), [CCNB1](https://www.affymetrix.com/LinkServlet?probeset=CCNB1), [CCND2](https://www.affymetrix.com/LinkServlet?probeset=CCND2), [PMAIP1](https://www.affymetrix.com/LinkServlet?probeset=PMAIP1), [CCNE1](https://www.affymetrix.com/LinkServlet?probeset=CCNE1), [CCNB2](https://www.affymetrix.com/LinkServlet?probeset=CCNB2), [ZMAT3](https://www.affymetrix.com/LinkServlet?probeset=ZMAT3), [CDK1](https://www.affymetrix.com/LinkServlet?probeset=CDK1), [CHEK1](https://www.affymetrix.com/LinkServlet?probeset=CHEK1), [PPM1D](https://www.affymetrix.com/LinkServlet?probeset=PPM1D), [SIAH1](https://www.affymetrix.com/LinkServlet?probeset=SIAH1), [TP53I3](https://www.affymetrix.com/LinkServlet?probeset=TP53I3), [CCND1](https://www.affymetrix.com/LinkServlet?probeset=CCND1), [CHEK2](https://www.affymetrix.com/LinkServlet?probeset=CHEK2), and [CDKN1A](https://www.affymetrix.com/LinkServlet?probeset=CDKN1A) |
| Pathogenic *Escherichia coli* infection | 1.98E+00 | 0.00E+00 | 7.45E-01 | [TUBB2B](https://www.affymetrix.com/LinkServlet?probeset=TUBB2B), [CDH1](https://www.affymetrix.com/LinkServlet?probeset=CDH1), [EZR](https://www.affymetrix.com/LinkServlet?probeset=EZR), [OCLN](https://www.affymetrix.com/LinkServlet?probeset=OCLN), [YWHAZ](https://www.affymetrix.com/LinkServlet?probeset=YWHAZ), [YWHAQ](https://www.affymetrix.com/LinkServlet?probeset=YWHAQ), [CLDN1](https://www.affymetrix.com/LinkServlet?probeset=CLDN1), [ARPC1B](https://www.affymetrix.com/LinkServlet?probeset=ARPC1B), [TUBA1A](https://www.affymetrix.com/LinkServlet?probeset=TUBA1A), [RHOA](https://www.affymetrix.com/LinkServlet?probeset=RHOA), [ARPC3](https://www.affymetrix.com/LinkServlet?probeset=ARPC3), [KRT18](https://www.affymetrix.com/LinkServlet?probeset=KRT18), [TLR5](https://www.affymetrix.com/LinkServlet?probeset=TLR5), [NCL](https://www.affymetrix.com/LinkServlet?probeset=NCL), [ARPC5](https://www.affymetrix.com/LinkServlet?probeset=ARPC5), [CTNNB1](https://www.affymetrix.com/LinkServlet?probeset=CTNNB1), [TUBB](https://www.affymetrix.com/LinkServlet?probeset=TUBB), [TUBA1B](https://www.affymetrix.com/LinkServlet?probeset=TUBA1B), [ARPC1A](https://www.affymetrix.com/LinkServlet?probeset=ARPC1A), [ITGB1](https://www.affymetrix.com/LinkServlet?probeset=ITGB1), [HCLS1](https://www.affymetrix.com/LinkServlet?probeset=HCLS1), [ARPC2](https://www.affymetrix.com/LinkServlet?probeset=ARPC2), [TUBA1C](https://www.affymetrix.com/LinkServlet?probeset=TUBA1C), and [WASL](https://www.affymetrix.com/LinkServlet?probeset=WASL) |
| Mismatch repair | 1.81E+00 | 0.00E+00 | 3.09E-02 | [PCNA](https://www.affymetrix.com/LinkServlet?probeset=PCNA), [RPA2](https://www.affymetrix.com/LinkServlet?probeset=RPA2), [MLH1](https://www.affymetrix.com/LinkServlet?probeset=MLH1), [RFC2](https://www.affymetrix.com/LinkServlet?probeset=RFC2), [MSH2](https://www.affymetrix.com/LinkServlet?probeset=MSH2), [RFC4](https://www.affymetrix.com/LinkServlet?probeset=RFC4), [RFC3](https://www.affymetrix.com/LinkServlet?probeset=RFC3), [RPA1](https://www.affymetrix.com/LinkServlet?probeset=RPA1), [MSH6](https://www.affymetrix.com/LinkServlet?probeset=MSH6), and [LIG1](https://www.affymetrix.com/LinkServlet?probeset=LIG1) |
| ECM receptor interaction | 1.66E+00 | 0.00E+00 | 5.91E-02 | [TNC](https://www.affymetrix.com/LinkServlet?probeset=TNC), [ITGA2](https://www.affymetrix.com/LinkServlet?probeset=ITGA2), [THBS1](https://www.affymetrix.com/LinkServlet?probeset=THBS1), [THBS2](https://www.affymetrix.com/LinkServlet?probeset=THBS2), [LAMC2](https://www.affymetrix.com/LinkServlet?probeset=LAMC2), [LAMA4](https://www.affymetrix.com/LinkServlet?probeset=LAMA4), [ITGA1](https://www.affymetrix.com/LinkServlet?probeset=ITGA1), [SDC3](https://www.affymetrix.com/LinkServlet?probeset=SDC3), [VWF](https://www.affymetrix.com/LinkServlet?probeset=VWF), [COL4A1](https://www.affymetrix.com/LinkServlet?probeset=COL4A1), [COL5A2](https://www.affymetrix.com/LinkServlet?probeset=COL5A2), [LAMB1](https://www.affymetrix.com/LinkServlet?probeset=LAMB1), [LAMA5](https://www.affymetrix.com/LinkServlet?probeset=LAMA5), [LAMC1](https://www.affymetrix.com/LinkServlet?probeset=LAMC1), [CD47](https://www.affymetrix.com/LinkServlet?probeset=CD47), [COL6A3](https://www.affymetrix.com/LinkServlet?probeset=COL6A3), [COL1A2](https://www.affymetrix.com/LinkServlet?probeset=COL1A2), [LAMA2](https://www.affymetrix.com/LinkServlet?probeset=LAMA2), [COL4A6](https://www.affymetrix.com/LinkServlet?probeset=COL4A6), [HMMR](https://www.affymetrix.com/LinkServlet?probeset=HMMR), [LAMA3](https://www.affymetrix.com/LinkServlet?probeset=LAMA3), [COL3A1](https://www.affymetrix.com/LinkServlet?probeset=COL3A1), [ITGB1](https://www.affymetrix.com/LinkServlet?probeset=ITGB1), [FN1](https://www.affymetrix.com/LinkServlet?probeset=FN1), [CD44](https://www.affymetrix.com/LinkServlet?probeset=CD44), [ITGB6](https://www.affymetrix.com/LinkServlet?probeset=ITGB6), and [COL5A1](https://www.affymetrix.com/LinkServlet?probeset=COL5A1) |
| Spliceosome | 1.65E+00 | 0.00E+00 | 5.68E-02 | [SRSF4](https://www.affymetrix.com/LinkServlet?probeset=SRSF4), [SNRNP40](https://www.affymetrix.com/LinkServlet?probeset=SNRNP40), [SNW1](https://www.affymetrix.com/LinkServlet?probeset=SNW1), [TRA2A](https://www.affymetrix.com/LinkServlet?probeset=TRA2A), [HNRNPA3](https://www.affymetrix.com/LinkServlet?probeset=HNRNPA3), [RBM25](https://www.affymetrix.com/LinkServlet?probeset=RBM25), [LSM5](https://www.affymetrix.com/LinkServlet?probeset=LSM5), [THOC1](https://www.affymetrix.com/LinkServlet?probeset=THOC1), [HNRNPU](https://www.affymetrix.com/LinkServlet?probeset=HNRNPU), [PRPF38B](https://www.affymetrix.com/LinkServlet?probeset=PRPF38B), [SRSF3](https://www.affymetrix.com/LinkServlet?probeset=SRSF3), [U2SURP](https://www.affymetrix.com/LinkServlet?probeset=U2SURP), [HNRNPC](https://www.affymetrix.com/LinkServlet?probeset=HNRNPC), [EIF4A3](https://www.affymetrix.com/LinkServlet?probeset=EIF4A3), [SNRNP200](https://www.affymetrix.com/LinkServlet?probeset=SNRNP200), [PPIH](https://www.affymetrix.com/LinkServlet?probeset=PPIH), [NCBP1](https://www.affymetrix.com/LinkServlet?probeset=NCBP1), [HNRNPA1](https://www.affymetrix.com/LinkServlet?probeset=HNRNPA1), [PPIE](https://www.affymetrix.com/LinkServlet?probeset=PPIE), [SRSF7](https://www.affymetrix.com/LinkServlet?probeset=SRSF7), [DDX23](https://www.affymetrix.com/LinkServlet?probeset=DDX23), [HNRNPM](https://www.affymetrix.com/LinkServlet?probeset=HNRNPM), [MAGOHB](https://www.affymetrix.com/LinkServlet?probeset=MAGOHB), [PRPF40A](https://www.affymetrix.com/LinkServlet?probeset=PRPF40A), [SNRPA1](https://www.affymetrix.com/LinkServlet?probeset=SNRPA1), [SF3A1](https://www.affymetrix.com/LinkServlet?probeset=SF3A1), [SRSF2](https://www.affymetrix.com/LinkServlet?probeset=SRSF2), [SNRPD1](https://www.affymetrix.com/LinkServlet?probeset=SNRPD1), [HSPA2](https://www.affymetrix.com/LinkServlet?probeset=HSPA2), [SNRPB2](https://www.affymetrix.com/LinkServlet?probeset=SNRPB2), [U2AF1](https://www.affymetrix.com/LinkServlet?probeset=U2AF1), [PRPF6](https://www.affymetrix.com/LinkServlet?probeset=PRPF6), [LSM2](https://www.affymetrix.com/LinkServlet?probeset=LSM2), [USP39](https://www.affymetrix.com/LinkServlet?probeset=USP39), [CDC5L](https://www.affymetrix.com/LinkServlet?probeset=CDC5L), [PRPF4](https://www.affymetrix.com/LinkServlet?probeset=PRPF4), [SRSF5](https://www.affymetrix.com/LinkServlet?probeset=SRSF5), [PRPF18](https://www.affymetrix.com/LinkServlet?probeset=PRPF18), [DHX15](https://www.affymetrix.com/LinkServlet?probeset=DHX15), [SRSF1](https://www.affymetrix.com/LinkServlet?probeset=SRSF1), and [CRNKL1](https://www.affymetrix.com/LinkServlet?probeset=CRNKL1) |
| Pathways in cancer | 1.46E+00 | 0.00E+00 | 1.46E-01 | [RB1](https://www.affymetrix.com/LinkServlet?probeset=RB1), [CASP8](https://www.affymetrix.com/LinkServlet?probeset=CASP8), [ITGA2](https://www.affymetrix.com/LinkServlet?probeset=ITGA2), [KRAS](https://www.affymetrix.com/LinkServlet?probeset=KRAS), [FAS](https://www.affymetrix.com/LinkServlet?probeset=FAS), [FZD6](https://www.affymetrix.com/LinkServlet?probeset=FZD6), [EGLN3](https://www.affymetrix.com/LinkServlet?probeset=EGLN3), [TGFB2](https://www.affymetrix.com/LinkServlet?probeset=TGFB2), [CDH1](https://www.affymetrix.com/LinkServlet?probeset=CDH1), [GSK3B](https://www.affymetrix.com/LinkServlet?probeset=GSK3B), [HGF](https://www.affymetrix.com/LinkServlet?probeset=HGF), [EGLN1](https://www.affymetrix.com/LinkServlet?probeset=EGLN1), [MYC](https://www.affymetrix.com/LinkServlet?probeset=MYC), [CASP3](https://www.affymetrix.com/LinkServlet?probeset=CASP3), [IKBKB](https://www.affymetrix.com/LinkServlet?probeset=IKBKB), [MECOM](https://www.affymetrix.com/LinkServlet?probeset=MECOM), [RALBP1](https://www.affymetrix.com/LinkServlet?probeset=RALBP1), [RALB](https://www.affymetrix.com/LinkServlet?probeset=RALB), [MMP1](https://www.affymetrix.com/LinkServlet?probeset=MMP1), [IL8](https://www.affymetrix.com/LinkServlet?probeset=IL8), [LAMC2](https://www.affymetrix.com/LinkServlet?probeset=LAMC2), [LAMA4](https://www.affymetrix.com/LinkServlet?probeset=LAMA4), [HDAC2](https://www.affymetrix.com/LinkServlet?probeset=HDAC2), [FGF9](https://www.affymetrix.com/LinkServlet?probeset=FGF9), [COL4A1](https://www.affymetrix.com/LinkServlet?probeset=COL4A1), [FZD7](https://www.affymetrix.com/LinkServlet?probeset=FZD7), [MLH1](https://www.affymetrix.com/LinkServlet?probeset=MLH1), [KIT](https://www.affymetrix.com/LinkServlet?probeset=KIT), [CTBP2](https://www.affymetrix.com/LinkServlet?probeset=CTBP2), [LAMB1](https://www.affymetrix.com/LinkServlet?probeset=LAMB1), [CCNE1](https://www.affymetrix.com/LinkServlet?probeset=CCNE1), [MET](https://www.affymetrix.com/LinkServlet?probeset=MET), [FZD1](https://www.affymetrix.com/LinkServlet?probeset=FZD1), [MITF](https://www.affymetrix.com/LinkServlet?probeset=MITF), [RARB](https://www.affymetrix.com/LinkServlet?probeset=RARB), [TRAF6](https://www.affymetrix.com/LinkServlet?probeset=TRAF6), [JAK1](https://www.affymetrix.com/LinkServlet?probeset=JAK1), [TGFA](https://www.affymetrix.com/LinkServlet?probeset=TGFA), [CKS1B](https://www.affymetrix.com/LinkServlet?probeset=CKS1B), [TPR](https://www.affymetrix.com/LinkServlet?probeset=TPR), [PDGFA](https://www.affymetrix.com/LinkServlet?probeset=PDGFA), [APC](https://www.affymetrix.com/LinkServlet?probeset=APC), [ARNT](https://www.affymetrix.com/LinkServlet?probeset=ARNT), [MSH2](https://www.affymetrix.com/LinkServlet?probeset=MSH2), [LAMA5](https://www.affymetrix.com/LinkServlet?probeset=LAMA5), [JUN](https://www.affymetrix.com/LinkServlet?probeset=JUN), [FGF22](https://www.affymetrix.com/LinkServlet?probeset=FGF22), [LAMC1](https://www.affymetrix.com/LinkServlet?probeset=LAMC1), [HSP90B1](https://www.affymetrix.com/LinkServlet?probeset=HSP90B1), [RHOA](https://www.affymetrix.com/LinkServlet?probeset=RHOA), [BIRC3](https://www.affymetrix.com/LinkServlet?probeset=BIRC3), [XIAP](https://www.affymetrix.com/LinkServlet?probeset=XIAP), [BRAF](https://www.affymetrix.com/LinkServlet?probeset=BRAF), [WNT2B](https://www.affymetrix.com/LinkServlet?probeset=WNT2B), [SMAD2](https://www.affymetrix.com/LinkServlet?probeset=SMAD2), [EGFR](https://www.affymetrix.com/LinkServlet?probeset=EGFR), [CCND1](https://www.affymetrix.com/LinkServlet?probeset=CCND1), [RALA](https://www.affymetrix.com/LinkServlet?probeset=RALA), [LAMA2](https://www.affymetrix.com/LinkServlet?probeset=LAMA2), [COL4A6](https://www.affymetrix.com/LinkServlet?probeset=COL4A6), [WNT5A](https://www.affymetrix.com/LinkServlet?probeset=WNT5A), [CTNNB1](https://www.affymetrix.com/LinkServlet?probeset=CTNNB1), [LAMA3](https://www.affymetrix.com/LinkServlet?probeset=LAMA3), [CDKN1A](https://www.affymetrix.com/LinkServlet?probeset=CDKN1A), [FZD3](https://www.affymetrix.com/LinkServlet?probeset=FZD3), [MSH6](https://www.affymetrix.com/LinkServlet?probeset=MSH6), [PPARG](https://www.affymetrix.com/LinkServlet?probeset=PPARG), [APPL1](https://www.affymetrix.com/LinkServlet?probeset=APPL1), [BCR](https://www.affymetrix.com/LinkServlet?probeset=BCR), [PDGFRA](https://www.affymetrix.com/LinkServlet?probeset=PDGFRA), [HSP90AB1](https://www.affymetrix.com/LinkServlet?probeset=HSP90AB1), [MAPK1](https://www.affymetrix.com/LinkServlet?probeset=MAPK1), [PTGS2](https://www.affymetrix.com/LinkServlet?probeset=PTGS2), [E2F3](https://www.affymetrix.com/LinkServlet?probeset=E2F3), [ITGB1](https://www.affymetrix.com/LinkServlet?probeset=ITGB1), [CTBP1](https://www.affymetrix.com/LinkServlet?probeset=CTBP1), [CDKN1B](https://www.affymetrix.com/LinkServlet?probeset=CDKN1B), [TCEB1](https://www.affymetrix.com/LinkServlet?probeset=TCEB1), [LEF1](https://www.affymetrix.com/LinkServlet?probeset=LEF1), [FN1](https://www.affymetrix.com/LinkServlet?probeset=FN1), [CUL2](https://www.affymetrix.com/LinkServlet?probeset=CUL2), [TCF7L2](https://www.affymetrix.com/LinkServlet?probeset=TCF7L2), and [WNT4](https://www.affymetrix.com/LinkServlet?probeset=WNT4) |
| Ubiquitin mediated proteolysis | 1.43E+00 | 3.20E-03 | 1.66E-01 | [MID1](https://www.affymetrix.com/LinkServlet?probeset=MID1), [FANCL](https://www.affymetrix.com/LinkServlet?probeset=FANCL), [SAE1](https://www.affymetrix.com/LinkServlet?probeset=SAE1), [UBA3](https://www.affymetrix.com/LinkServlet?probeset=UBA3), [TRIM37](https://www.affymetrix.com/LinkServlet?probeset=TRIM37), [SMURF1](https://www.affymetrix.com/LinkServlet?probeset=SMURF1), [TRAF6](https://www.affymetrix.com/LinkServlet?probeset=TRAF6), [RNF7](https://www.affymetrix.com/LinkServlet?probeset=RNF7), [UBE4B](https://www.affymetrix.com/LinkServlet?probeset=UBE4B), [UBE2W](https://www.affymetrix.com/LinkServlet?probeset=UBE2W), [SIAH1](https://www.affymetrix.com/LinkServlet?probeset=SIAH1), [CUL4B](https://www.affymetrix.com/LinkServlet?probeset=CUL4B), [UBE2J1](https://www.affymetrix.com/LinkServlet?probeset=UBE2J1), [BIRC3](https://www.affymetrix.com/LinkServlet?probeset=BIRC3), [CUL4A](https://www.affymetrix.com/LinkServlet?probeset=CUL4A), [XIAP](https://www.affymetrix.com/LinkServlet?probeset=XIAP), [ITCH](https://www.affymetrix.com/LinkServlet?probeset=ITCH), [BRCA1](https://www.affymetrix.com/LinkServlet?probeset=BRCA1), [CDC16](https://www.affymetrix.com/LinkServlet?probeset=CDC16), [UBE2E1](https://www.affymetrix.com/LinkServlet?probeset=UBE2E1), [UBE2H](https://www.affymetrix.com/LinkServlet?probeset=UBE2H), [UBR5](https://www.affymetrix.com/LinkServlet?probeset=UBR5), [UBE3C](https://www.affymetrix.com/LinkServlet?probeset=UBE3C), [UBE2L6](https://www.affymetrix.com/LinkServlet?probeset=UBE2L6), [WWP1](https://www.affymetrix.com/LinkServlet?probeset=WWP1), [UBE2D4](https://www.affymetrix.com/LinkServlet?probeset=UBE2D4), [TRIM32](https://www.affymetrix.com/LinkServlet?probeset=TRIM32), [CDC20](https://www.affymetrix.com/LinkServlet?probeset=CDC20), [CUL1](https://www.affymetrix.com/LinkServlet?probeset=CUL1), [UBA2](https://www.affymetrix.com/LinkServlet?probeset=UBA2), [TCEB1](https://www.affymetrix.com/LinkServlet?probeset=TCEB1), [CUL2](https://www.affymetrix.com/LinkServlet?probeset=CUL2), [UBE2D1](https://www.affymetrix.com/LinkServlet?probeset=UBE2D1), [SMURF2](https://www.affymetrix.com/LinkServlet?probeset=SMURF2), [SOCS1](https://www.affymetrix.com/LinkServlet?probeset=SOCS1), [MDM2](https://www.affymetrix.com/LinkServlet?probeset=MDM2), [CUL5](https://www.affymetrix.com/LinkServlet?probeset=CUL5), [SKP2](https://www.affymetrix.com/LinkServlet?probeset=SKP2), [CBLC](https://www.affymetrix.com/LinkServlet?probeset=CBLC), [TRIP12](https://www.affymetrix.com/LinkServlet?probeset=TRIP12), [CUL3](https://www.affymetrix.com/LinkServlet?probeset=CUL3), [UBE3A](https://www.affymetrix.com/LinkServlet?probeset=UBE3A), [ANAPC10](https://www.affymetrix.com/LinkServlet?probeset=ANAPC10), [UBE2B](https://www.affymetrix.com/LinkServlet?probeset=UBE2B), [PIAS1](https://www.affymetrix.com/LinkServlet?probeset=PIAS1), [NEDD4L](https://www.affymetrix.com/LinkServlet?probeset=NEDD4L), [HERC4](https://www.affymetrix.com/LinkServlet?probeset=HERC4), and [BIRC2](https://www.affymetrix.com/LinkServlet?probeset=BIRC2) |
| Glycosminoglycan biosynthesis chondroitin sulfate | 1.69E+00 | 7.60E-03 | 5.97E-02 | [CSGALNACT1](https://www.affymetrix.com/LinkServlet?probeset=CSGALNACT1), [CHST3](https://www.affymetrix.com/LinkServlet?probeset=CHST3), [CHSY1](https://www.affymetrix.com/LinkServlet?probeset=CHSY1), [CHST15](https://www.affymetrix.com/LinkServlet?probeset=CHST15), [CHST12](https://www.affymetrix.com/LinkServlet?probeset=CHST12), [UST](https://www.affymetrix.com/LinkServlet?probeset=UST), [DSE](https://www.affymetrix.com/LinkServlet?probeset=DSE), [CSGALNACT2](https://www.affymetrix.com/LinkServlet?probeset=CSGALNACT2), and [XYLT1](https://www.affymetrix.com/LinkServlet?probeset=XYLT1) |
| O-glycan biosynthesis | 1.76E+00 | 9.50E-03 | 4.28E-02 | [GALNT3](https://www.affymetrix.com/LinkServlet?probeset=GALNT3), [GALNT12](https://www.affymetrix.com/LinkServlet?probeset=GALNT12), [C1GALT1C1](https://www.affymetrix.com/LinkServlet?probeset=C1GALT1C1), [GALNT1](https://www.affymetrix.com/LinkServlet?probeset=GALNT1), [B4GALT5](https://www.affymetrix.com/LinkServlet?probeset=B4GALT5), [GCNT3](https://www.affymetrix.com/LinkServlet?probeset=GCNT3), [GALNT10](https://www.affymetrix.com/LinkServlet?probeset=GALNT10), [GALNT7](https://www.affymetrix.com/LinkServlet?probeset=GALNT7), and [GCNT1](https://www.affymetrix.com/LinkServlet?probeset=GCNT1) |
| Bladder cancer | 1.69E+00 | 1.08E-02 | 5.35E-02 | [RB1](https://www.affymetrix.com/LinkServlet?probeset=RB1), [KRAS](https://www.affymetrix.com/LinkServlet?probeset=KRAS), [CDH1](https://www.affymetrix.com/LinkServlet?probeset=CDH1), [MYC](https://www.affymetrix.com/LinkServlet?probeset=MYC), [THBS1](https://www.affymetrix.com/LinkServlet?probeset=THBS1), [MMP1](https://www.affymetrix.com/LinkServlet?probeset=MMP1), [IL8](https://www.affymetrix.com/LinkServlet?probeset=IL8), [RPS6KA5](https://www.affymetrix.com/LinkServlet?probeset=RPS6KA5), [BRAF](https://www.affymetrix.com/LinkServlet?probeset=BRAF), [EGFR](https://www.affymetrix.com/LinkServlet?probeset=EGFR), [CCND1](https://www.affymetrix.com/LinkServlet?probeset=CCND1), [TYMP](https://www.affymetrix.com/LinkServlet?probeset=TYMP), [CDKN1A](https://www.affymetrix.com/LinkServlet?probeset=CDKN1A), [MAPK1](https://www.affymetrix.com/LinkServlet?probeset=MAPK1), and [E2F3](https://www.affymetrix.com/LinkServlet?probeset=E2F3) |
| Nod-like receptor signaling pathway | 1.57E+00 | 1.16E-02 | 9.35E-02 | [CCL2](https://www.affymetrix.com/LinkServlet?probeset=CCL2), [CASP8](https://www.affymetrix.com/LinkServlet?probeset=CASP8), [IKBKB](https://www.affymetrix.com/LinkServlet?probeset=IKBKB), [CASP1](https://www.affymetrix.com/LinkServlet?probeset=CASP1), [IL8](https://www.affymetrix.com/LinkServlet?probeset=IL8), [CXCL1](https://www.affymetrix.com/LinkServlet?probeset=CXCL1), [IL18](https://www.affymetrix.com/LinkServlet?probeset=IL18), [MAPK13](https://www.affymetrix.com/LinkServlet?probeset=MAPK13), [TRAF6](https://www.affymetrix.com/LinkServlet?probeset=TRAF6), [HSP90B1](https://www.affymetrix.com/LinkServlet?probeset=HSP90B1), [BIRC3](https://www.affymetrix.com/LinkServlet?probeset=BIRC3), [XIAP](https://www.affymetrix.com/LinkServlet?probeset=XIAP), [CCL8](https://www.affymetrix.com/LinkServlet?probeset=CCL8), [NAIP](https://www.affymetrix.com/LinkServlet?probeset=NAIP), [TNFAIP3](https://www.affymetrix.com/LinkServlet?probeset=TNFAIP3), [HSP90AB1](https://www.affymetrix.com/LinkServlet?probeset=HSP90AB1), [MAPK1](https://www.affymetrix.com/LinkServlet?probeset=MAPK1), [CCL5](https://www.affymetrix.com/LinkServlet?probeset=CCL5), [RIPK2](https://www.affymetrix.com/LinkServlet?probeset=RIPK2), [NLRP3](https://www.affymetrix.com/LinkServlet?probeset=NLRP3), [HSP90AA1](https://www.affymetrix.com/LinkServlet?probeset=HSP90AA1), [IL1B](https://www.affymetrix.com/LinkServlet?probeset=IL1B), [MAP3K7](https://www.affymetrix.com/LinkServlet?probeset=MAP3K7), [CHUK](https://www.affymetrix.com/LinkServlet?probeset=CHUK), [MAPK14](https://www.affymetrix.com/LinkServlet?probeset=MAPK14), [CARD8](https://www.affymetrix.com/LinkServlet?probeset=CARD8), [BIRC2](https://www.affymetrix.com/LinkServlet?probeset=BIRC2), and [CXCL2](https://www.affymetrix.com/LinkServlet?probeset=CXCL2) |
| DNA replication | 1.65E+00 | 1.57-02 | 5.84E-02 | [MCM3](https://www.affymetrix.com/LinkServlet?probeset=MCM3), [PCNA](https://www.affymetrix.com/LinkServlet?probeset=PCNA), [RPA2](https://www.affymetrix.com/LinkServlet?probeset=RPA2), [RFC2](https://www.affymetrix.com/LinkServlet?probeset=RFC2), [POLE3](https://www.affymetrix.com/LinkServlet?probeset=POLE3), [RFC4](https://www.affymetrix.com/LinkServlet?probeset=RFC4), [RFC3](https://www.affymetrix.com/LinkServlet?probeset=RFC3), [RPA1](https://www.affymetrix.com/LinkServlet?probeset=RPA1), [POLA1](https://www.affymetrix.com/LinkServlet?probeset=POLA1), [PRIM2](https://www.affymetrix.com/LinkServlet?probeset=PRIM2), [LIG1](https://www.affymetrix.com/LinkServlet?probeset=LIG1), [RNASEH2B](https://www.affymetrix.com/LinkServlet?probeset=RNASEH2B), [RNASEH2A](https://www.affymetrix.com/LinkServlet?probeset=RNASEH2A), [POLE2](https://www.affymetrix.com/LinkServlet?probeset=POLE2), [PRIM1](https://www.affymetrix.com/LinkServlet?probeset=PRIM1), [MCM5](https://www.affymetrix.com/LinkServlet?probeset=MCM5), [RFC5](https://www.affymetrix.com/LinkServlet?probeset=RFC5), [FEN1](https://www.affymetrix.com/LinkServlet?probeset=FEN1), and [RPA4](https://www.affymetrix.com/LinkServlet?probeset=RPA4) |
| Small cell lung cancer | 1.49E+00 | 2.14E-02 | 1.46E-01 | [RB1](https://www.affymetrix.com/LinkServlet?probeset=RB1), [ITGA2](https://www.affymetrix.com/LinkServlet?probeset=ITGA2), [APAF1](https://www.affymetrix.com/LinkServlet?probeset=APAF1), [MYC](https://www.affymetrix.com/LinkServlet?probeset=MYC), [IKBKB](https://www.affymetrix.com/LinkServlet?probeset=IKBKB), [LAMC2](https://www.affymetrix.com/LinkServlet?probeset=LAMC2), [LAMA4](https://www.affymetrix.com/LinkServlet?probeset=LAMA4), [COL4A1](https://www.affymetrix.com/LinkServlet?probeset=COL4A1), [LAMB1](https://www.affymetrix.com/LinkServlet?probeset=LAMB1), [CCNE1](https://www.affymetrix.com/LinkServlet?probeset=CCNE1), [RARB](https://www.affymetrix.com/LinkServlet?probeset=RARB), [TRAF6](https://www.affymetrix.com/LinkServlet?probeset=TRAF6), [CKS1B](https://www.affymetrix.com/LinkServlet?probeset=CKS1B), [LAMA5](https://www.affymetrix.com/LinkServlet?probeset=LAMA5), [LAMC1](https://www.affymetrix.com/LinkServlet?probeset=LAMC1), [BIRC3](https://www.affymetrix.com/LinkServlet?probeset=BIRC3), [XIAP](https://www.affymetrix.com/LinkServlet?probeset=XIAP), [CCND1](https://www.affymetrix.com/LinkServlet?probeset=CCND1), [LAMA2](https://www.affymetrix.com/LinkServlet?probeset=LAMA2), [COL4A6](https://www.affymetrix.com/LinkServlet?probeset=COL4A6), [LAMA3](https://www.affymetrix.com/LinkServlet?probeset=LAMA3), [PTGS2](https://www.affymetrix.com/LinkServlet?probeset=PTGS2), [E2F3](https://www.affymetrix.com/LinkServlet?probeset=E2F3), [ITGB1](https://www.affymetrix.com/LinkServlet?probeset=ITGB1), [CDKN1B](https://www.affymetrix.com/LinkServlet?probeset=CDKN1B), and [FN1](https://www.affymetrix.com/LinkServlet?probeset=FN1) |
| Colorectal cancer | 1.47E+00 | 2.37E-02 | 1.53E-01 | [KRAS](https://www.affymetrix.com/LinkServlet?probeset=KRAS), [TGFB2](https://www.affymetrix.com/LinkServlet?probeset=TGFB2), [GSK3B](https://www.affymetrix.com/LinkServlet?probeset=GSK3B), [MYC](https://www.affymetrix.com/LinkServlet?probeset=MYC), [CASP3](https://www.affymetrix.com/LinkServlet?probeset=CASP3), [MLH1](https://www.affymetrix.com/LinkServlet?probeset=MLH1), [APC](https://www.affymetrix.com/LinkServlet?probeset=APC), [MSH2](https://www.affymetrix.com/LinkServlet?probeset=MSH2), [JUN](https://www.affymetrix.com/LinkServlet?probeset=JUN), [RHOA](https://www.affymetrix.com/LinkServlet?probeset=RHOA), [BRAF](https://www.affymetrix.com/LinkServlet?probeset=BRAF), [SMAD2](https://www.affymetrix.com/LinkServlet?probeset=SMAD2), [CCND1](https://www.affymetrix.com/LinkServlet?probeset=CCND1), [CTNNB1](https://www.affymetrix.com/LinkServlet?probeset=CTNNB1), [MSH6](https://www.affymetrix.com/LinkServlet?probeset=MSH6), [APPL1](https://www.affymetrix.com/LinkServlet?probeset=APPL1), [MAPK1](https://www.affymetrix.com/LinkServlet?probeset=MAPK1), [LEF1](https://www.affymetrix.com/LinkServlet?probeset=LEF1), [TCF7L2](https://www.affymetrix.com/LinkServlet?probeset=TCF7L2), [SMAD4](https://www.affymetrix.com/LinkServlet?probeset=SMAD4), and [PIK3R1](https://www.affymetrix.com/LinkServlet?probeset=PIK3R1) |
| Apoptosis | 1.46E+00 | 2.80E-02 | 1.56E-01 | [CASP8](https://www.affymetrix.com/LinkServlet?probeset=CASP8), [PRKACB](https://www.affymetrix.com/LinkServlet?probeset=PRKACB), [FAS](https://www.affymetrix.com/LinkServlet?probeset=FAS), [PRKX](https://www.affymetrix.com/LinkServlet?probeset=PRKX), [APAF1](https://www.affymetrix.com/LinkServlet?probeset=APAF1), [CASP6](https://www.affymetrix.com/LinkServlet?probeset=CASP6), [IL1R1](https://www.affymetrix.com/LinkServlet?probeset=IL1R1), [CASP3](https://www.affymetrix.com/LinkServlet?probeset=CASP3), [IKBKB](https://www.affymetrix.com/LinkServlet?probeset=IKBKB), [TNFSF10](https://www.affymetrix.com/LinkServlet?probeset=TNFSF10), [ATM](https://www.affymetrix.com/LinkServlet?probeset=ATM), [PPP3CC](https://www.affymetrix.com/LinkServlet?probeset=PPP3CC), and [CFLAR](https://www.affymetrix.com/LinkServlet?probeset=CFLAR) |
| Nucleotide excision repair | 1.42E+00 | 3.50E-02 | 1.68E-01 | [ERCC6](https://www.affymetrix.com/LinkServlet?probeset=ERCC6), [PCNA](https://www.affymetrix.com/LinkServlet?probeset=PCNA), [RPA2](https://www.affymetrix.com/LinkServlet?probeset=RPA2), [RFC2](https://www.affymetrix.com/LinkServlet?probeset=RFC2), [POLE3](https://www.affymetrix.com/LinkServlet?probeset=POLE3), [RFC4](https://www.affymetrix.com/LinkServlet?probeset=RFC4), [RFC3](https://www.affymetrix.com/LinkServlet?probeset=RFC3), [RPA1](https://www.affymetrix.com/LinkServlet?probeset=RPA1), [CUL4B](https://www.affymetrix.com/LinkServlet?probeset=CUL4B), [CUL4A](https://www.affymetrix.com/LinkServlet?probeset=CUL4A), [CDK7](https://www.affymetrix.com/LinkServlet?probeset=CDK7), [ERCC3](https://www.affymetrix.com/LinkServlet?probeset=ERCC3), and [CETN2](https://www.affymetrix.com/LinkServlet?probeset=CETN2) |

**Supplementary Table 3.** Selected enriched pathways in PLK2 low group in kidney tissues

| Pathway | NES | *P*-value | FDR q-value | Molecules |
| --- | --- | --- | --- | --- |
| Neuroactive ligand receptor interaction | -2.27E+00 | 0.00E+00 | 0.00E+00 | [CHRM2](https://www.affymetrix.com/LinkServlet?probeset=CHRM2), [SSTR2](https://www.affymetrix.com/LinkServlet?probeset=SSTR2), [ADRB1](https://www.affymetrix.com/LinkServlet?probeset=ADRB1), [PRSS2](https://www.affymetrix.com/LinkServlet?probeset=PRSS2), [NPFFR1](https://www.affymetrix.com/LinkServlet?probeset=NPFFR1), [TACR1](https://www.affymetrix.com/LinkServlet?probeset=TACR1), [TACR2](https://www.affymetrix.com/LinkServlet?probeset=TACR2), [GALR1](https://www.affymetrix.com/LinkServlet?probeset=GALR1), [CCKAR](https://www.affymetrix.com/LinkServlet?probeset=CCKAR), [GABBR2](https://www.affymetrix.com/LinkServlet?probeset=GABBR2), [P2RX6](https://www.affymetrix.com/LinkServlet?probeset=P2RX6), [OPRM1](https://www.affymetrix.com/LinkServlet?probeset=OPRM1), [MC1R](https://www.affymetrix.com/LinkServlet?probeset=MC1R), [OPRL1](https://www.affymetrix.com/LinkServlet?probeset=OPRL1), [MCHR1](https://www.affymetrix.com/LinkServlet?probeset=MCHR1), [NTSR2](https://www.affymetrix.com/LinkServlet?probeset=NTSR2), [GABRA4](https://www.affymetrix.com/LinkServlet?probeset=GABRA4), [HTR4](https://www.affymetrix.com/LinkServlet?probeset=HTR4), [GHR](https://www.affymetrix.com/LinkServlet?probeset=GHR), [CHRM5](https://www.affymetrix.com/LinkServlet?probeset=CHRM5), [GRM7](https://www.affymetrix.com/LinkServlet?probeset=GRM7), [GRM1](https://www.affymetrix.com/LinkServlet?probeset=GRM1), [CHRNA1](https://www.affymetrix.com/LinkServlet?probeset=CHRNA1), [GRIA1](https://www.affymetrix.com/LinkServlet?probeset=GRIA1), [SSTR3](https://www.affymetrix.com/LinkServlet?probeset=SSTR3), [HCRTR2](https://www.affymetrix.com/LinkServlet?probeset=HCRTR2), [TRHR](https://www.affymetrix.com/LinkServlet?probeset=TRHR), [TBXA2R](https://www.affymetrix.com/LinkServlet?probeset=TBXA2R), [GABRD](https://www.affymetrix.com/LinkServlet?probeset=GABRD), [GRPR](https://www.affymetrix.com/LinkServlet?probeset=GRPR), [CHRNA9](https://www.affymetrix.com/LinkServlet?probeset=CHRNA9), [GRIK3](https://www.affymetrix.com/LinkServlet?probeset=GRIK3), [TSHB](https://www.affymetrix.com/LinkServlet?probeset=TSHB), [P2RY4](https://www.affymetrix.com/LinkServlet?probeset=P2RY4), [PRSS3](https://www.affymetrix.com/LinkServlet?probeset=PRSS3), [GH2](https://www.affymetrix.com/LinkServlet?probeset=GH2), [GH1](https://www.affymetrix.com/LinkServlet?probeset=GH1), [LHB](https://www.affymetrix.com/LinkServlet?probeset=LHB), [GLRA3](https://www.affymetrix.com/LinkServlet?probeset=GLRA3), [GLRA2](https://www.affymetrix.com/LinkServlet?probeset=GLRA3), [GRIN2A](https://www.affymetrix.com/LinkServlet?probeset=GRIN2A), [GRM4](https://www.affymetrix.com/LinkServlet?probeset=GRM4), [CRHR2](https://www.affymetrix.com/LinkServlet?probeset=CRHR2), [CHRNE](https://www.affymetrix.com/LinkServlet?probeset=CHRNE), [GABRA3](https://www.affymetrix.com/LinkServlet?probeset=GABRA3), [GRM2](https://www.affymetrix.com/LinkServlet?probeset=GRM2), [GALR3](https://www.affymetrix.com/LinkServlet?probeset=GALR3), [GRM8](https://www.affymetrix.com/LinkServlet?probeset=GRM8), [PTAFR](https://www.affymetrix.com/LinkServlet?probeset=PTAFR), [PTAFR](https://www.affymetrix.com/LinkServlet?probeset=PTAFR), [GABRQ](https://www.affymetrix.com/LinkServlet?probeset=GABRQ), [GRID2](https://www.affymetrix.com/LinkServlet?probeset=GRID2), [HTR2A](https://www.affymetrix.com/LinkServlet?probeset=HTR2A), [PRL](https://www.affymetrix.com/LinkServlet?probeset=PRL), [MC2R](https://www.affymetrix.com/LinkServlet?probeset=MC2R), [GIPR](https://www.affymetrix.com/LinkServlet?probeset=GIPR), [SSTR4](https://www.affymetrix.com/LinkServlet?probeset=SSTR4), [OPRD1](https://www.affymetrix.com/LinkServlet?probeset=OPRD1), [GRIN2C](https://www.affymetrix.com/LinkServlet?probeset=GRIN2C), [CHRNA3](https://www.affymetrix.com/LinkServlet?probeset=CHRNA3), [CHRNB3](https://www.affymetrix.com/LinkServlet?probeset=CHRNB3), [GABRA6](https://www.affymetrix.com/LinkServlet?probeset=GABRA6), [HTR6](https://www.affymetrix.com/LinkServlet?probeset=HTR6), [GHRHR](https://www.affymetrix.com/LinkServlet?probeset=GHRHR), [GNRHR](https://www.affymetrix.com/LinkServlet?probeset=GNRHR), [MTNR1B](https://www.affymetrix.com/LinkServlet?probeset=MTNR1B), [DRD2](https://www.affymetrix.com/LinkServlet?probeset=DRD2), [ADORA1](https://www.affymetrix.com/LinkServlet?probeset=ADORA1), [VIPR2](https://www.affymetrix.com/LinkServlet?probeset=VIPR2), [APLNR](https://www.affymetrix.com/LinkServlet?probeset=APLNR), [LTB4R2](https://www.affymetrix.com/LinkServlet?probeset=LTB4R2), [FSHB](https://www.affymetrix.com/LinkServlet?probeset=FSHB), [GABRG2](https://www.affymetrix.com/LinkServlet?probeset=GABRG2), [GRIK2](https://www.affymetrix.com/LinkServlet?probeset=GRIK2), [P2RX2](https://www.affymetrix.com/LinkServlet?probeset=P2RX2), [GLP2R](https://www.affymetrix.com/LinkServlet?probeset=GLP2R), [ADRA2C](https://www.affymetrix.com/LinkServlet?probeset=ADRA2C), [GLP1R](https://www.affymetrix.com/LinkServlet?probeset=GLP1R), [GABRA1](https://www.affymetrix.com/LinkServlet?probeset=GABRA1), [S1PR2](https://www.affymetrix.com/LinkServlet?probeset=S1PR2), [F2](https://www.affymetrix.com/LinkServlet?probeset=F2), [TACR3](https://www.affymetrix.com/LinkServlet?probeset=TACR3), [GRIN2D](https://www.affymetrix.com/LinkServlet?probeset=GRIN2D), [ADRA1D](https://www.affymetrix.com/LinkServlet?probeset=ADRA1D), [GRIK1](https://www.affymetrix.com/LinkServlet?probeset=GRIK1), [GABRR2](https://www.affymetrix.com/LinkServlet?probeset=GABRR2), [C5AR1](https://www.affymetrix.com/LinkServlet?probeset=C5AR1), [HTR1F](https://www.affymetrix.com/LinkServlet?probeset=HTR1F), [PTH1R](https://www.affymetrix.com/LinkServlet?probeset=PTH1R), [GRIA4](https://www.affymetrix.com/LinkServlet?probeset=GRIA4), [ADCYAP1R1](https://www.affymetrix.com/LinkServlet?probeset=ADCYAP1R1), [NPY2R](https://www.affymetrix.com/LinkServlet?probeset=NPY2R), [CHRND](https://www.affymetrix.com/LinkServlet?probeset=CHRND), [CCKBR](https://www.affymetrix.com/LinkServlet?probeset=CCKBR), [DRD1](https://www.affymetrix.com/LinkServlet?probeset=DRD1), [TAAR5](https://www.affymetrix.com/LinkServlet?probeset=TAAR5), [BRS3](https://www.affymetrix.com/LinkServlet?probeset=BRS3), [GABRR1](https://www.affymetrix.com/LinkServlet?probeset=GABRR1), [MC4R](https://www.affymetrix.com/LinkServlet?probeset=MC4R), [ADRB3](https://www.affymetrix.com/LinkServlet?probeset=ADRB3), [GLRA1](https://www.affymetrix.com/LinkServlet?probeset=GLRA1), [GRM5](https://www.affymetrix.com/LinkServlet?probeset=GRM5), [HTR1B](https://www.affymetrix.com/LinkServlet?probeset=HTR1B), [PTGIR](https://www.affymetrix.com/LinkServlet?probeset=PTGIR), [GABRB2](https://www.affymetrix.com/LinkServlet?probeset=GABRB2), [HRH4](https://www.affymetrix.com/LinkServlet?probeset=HRH4), [CGA](https://www.affymetrix.com/LinkServlet?probeset=CGA), [LEP](https://www.affymetrix.com/LinkServlet?probeset=LEP), [CHRNA10](https://www.affymetrix.com/LinkServlet?probeset=CHRNA10), [AVPR1B](https://www.affymetrix.com/LinkServlet?probeset=AVPR1B), [GABRA5](https://www.affymetrix.com/LinkServlet?probeset=GABRA5), [GRM6](https://www.affymetrix.com/LinkServlet?probeset=GRM6), [MC5R](https://www.affymetrix.com/LinkServlet?probeset=MC5R), [ADORA2B](https://www.affymetrix.com/LinkServlet?probeset=ADORA2B), [P2RY2](https://www.affymetrix.com/LinkServlet?probeset=P2RY2), [NTSR1](https://www.affymetrix.com/LinkServlet?probeset=NTSR1), [S1PR4](https://www.affymetrix.com/LinkServlet?probeset=S1PR4), [AGTR2](https://www.affymetrix.com/LinkServlet?probeset=AGTR2), [HTR5A](https://www.affymetrix.com/LinkServlet?probeset=HTR5A), [DRD5](https://www.affymetrix.com/LinkServlet?probeset=DRD5), [CHRNA4](https://www.affymetrix.com/LinkServlet?probeset=CHRNA4), [CHRNB4](https://www.affymetrix.com/LinkServlet?probeset=CHRNB4), [TAAR2](https://www.affymetrix.com/LinkServlet?probeset=TAAR2), [CHRNA6](https://www.affymetrix.com/LinkServlet?probeset=CHRNA6), [FPR3](https://www.affymetrix.com/LinkServlet?probeset=FPR3), and [GRIA2](https://www.affymetrix.com/LinkServlet?probeset=GRIA2) |
| Olfactory transduction | -2.24E+00 | 0.00E+00 | 0.00E+00 | [OR2J3](https://www.affymetrix.com/LinkServlet?probeset=OR2J3), [GNAL](https://www.affymetrix.com/LinkServlet?probeset=GNAL), [GNAL](https://www.affymetrix.com/LinkServlet?probeset=GNAL), [OR7A10](https://www.affymetrix.com/LinkServlet?probeset=OR7A10), [GUCY2D](https://www.affymetrix.com/LinkServlet?probeset=GUCY2D), [PDE1C](https://www.affymetrix.com/LinkServlet?probeset=PDE1C), [OR3A2](https://www.affymetrix.com/LinkServlet?probeset=OR3A2), [CALML5](https://www.affymetrix.com/LinkServlet?probeset=CALML5), [OR7A5](https://www.affymetrix.com/LinkServlet?probeset=OR7A5), [OR10C1](https://www.affymetrix.com/LinkServlet?probeset=OR10C1), [PDC](https://www.affymetrix.com/LinkServlet?probeset=PDC), [CNGB1](https://www.affymetrix.com/LinkServlet?probeset=CNGB1), [ADCY3](https://www.affymetrix.com/LinkServlet?probeset=ADCY3), [OR10H2](https://www.affymetrix.com/LinkServlet?probeset=OR10H2), [CAMK2B](https://www.affymetrix.com/LinkServlet?probeset=CAMK2B), [OR2B6](https://www.affymetrix.com/LinkServlet?probeset=OR2B6), [OR12D2](https://www.affymetrix.com/LinkServlet?probeset=OR12D2), [OR12D3](https://www.affymetrix.com/LinkServlet?probeset=OR12D3), [CLCA4](https://www.affymetrix.com/LinkServlet?probeset=CLCA4), [OR7C2](https://www.affymetrix.com/LinkServlet?probeset=OR7C2), [OR10J1](https://www.affymetrix.com/LinkServlet?probeset=OR10J1), [OR1G1](https://www.affymetrix.com/LinkServlet?probeset=OR1G1), [OR7C1](https://www.affymetrix.com/LinkServlet?probeset=OR7C1), [OR1F1](https://www.affymetrix.com/LinkServlet?probeset=OR1F1), [OR51E2](https://www.affymetrix.com/LinkServlet?probeset=OR51E2), [OR2J2](https://www.affymetrix.com/LinkServlet?probeset=OR2J2), [CAMK2A](https://www.affymetrix.com/LinkServlet?probeset=CAMK2A), [OR2H2](https://www.affymetrix.com/LinkServlet?probeset=OR2H2), [OR1D2](https://www.affymetrix.com/LinkServlet?probeset=OR1D2), [OR1E1](https://www.affymetrix.com/LinkServlet?probeset=OR1E1), [OR10H3](https://www.affymetrix.com/LinkServlet?probeset=OR10H3), [CLCA1](https://www.affymetrix.com/LinkServlet?probeset=CLCA1), [OR3A3](https://www.affymetrix.com/LinkServlet?probeset=OR3A3), [OR2W1](https://www.affymetrix.com/LinkServlet?probeset=OR2W1), [PRKACG](https://www.affymetrix.com/LinkServlet?probeset=PRKACG), [CALML3](https://www.affymetrix.com/LinkServlet?probeset=CALML3), [OR11A1](https://www.affymetrix.com/LinkServlet?probeset=OR11A1), [OR2S2](https://www.affymetrix.com/LinkServlet?probeset=OR2S2), [OR10H1](https://www.affymetrix.com/LinkServlet?probeset=OR10H1), [PRKACA](https://www.affymetrix.com/LinkServlet?probeset=PRKACA), [OR1A1](https://www.affymetrix.com/LinkServlet?probeset=OR1A1), [OR1A2](https://www.affymetrix.com/LinkServlet?probeset=OR1A2), [OR52A1](https://www.affymetrix.com/LinkServlet?probeset=OR52A1), and [OR7A17](https://www.affymetrix.com/LinkServlet?probeset=OR7A17) |
| Oxidative phosphorylation | -2.05E+00 | 0.00E+00 | 2.60E-03 | [ATP6V1E1](https://www.affymetrix.com/LinkServlet?probeset=ATP6V1E1), [NDUFB3](https://www.affymetrix.com/LinkServlet?probeset=NDUFB3), [ATP6V1F](https://www.affymetrix.com/LinkServlet?probeset=ATP6V1F), [NDUFA9](https://www.affymetrix.com/LinkServlet?probeset=NDUFA9), [NDUFB2](https://www.affymetrix.com/LinkServlet?probeset=NDUFB2), [ATP6V0E2](https://www.affymetrix.com/LinkServlet?probeset=ATP6V0E2), [UQCRC1](https://www.affymetrix.com/LinkServlet?probeset=UQCRC1), [ATP6V0C](https://www.affymetrix.com/LinkServlet?probeset=ATP6V0C), [COX5B](https://www.affymetrix.com/LinkServlet?probeset=COX5B), [NDUFA4L2](https://www.affymetrix.com/LinkServlet?probeset=NDUFA4L2), [COX7A2L](https://www.affymetrix.com/LinkServlet?probeset=COX7A2L), [COX17](https://www.affymetrix.com/LinkServlet?probeset=COX17), [NDUFAB1](https://www.affymetrix.com/LinkServlet?probeset=NDUFAB1), [NDUFA8](https://www.affymetrix.com/LinkServlet?probeset=NDUFA8), [COX7A1](https://www.affymetrix.com/LinkServlet?probeset=COX7A1), [ATP5E](https://www.affymetrix.com/LinkServlet?probeset=ATP5E), [NDUFA1](https://www.affymetrix.com/LinkServlet?probeset=NDUFA1), [ATP5H](https://www.affymetrix.com/LinkServlet?probeset=ATP5H), [NDUFA2](https://www.affymetrix.com/LinkServlet?probeset=NDUFA2), [ATP5J](https://www.affymetrix.com/LinkServlet?probeset=ATP5J), [CYC1](https://www.affymetrix.com/LinkServlet?probeset=CYC1), [ATP5L](https://www.affymetrix.com/LinkServlet?probeset=ATP5L), [COX7B](https://www.affymetrix.com/LinkServlet?probeset=COX7B), [ATP5D](https://www.affymetrix.com/LinkServlet?probeset=ATP5D), [NDUFB4](https://www.affymetrix.com/LinkServlet?probeset=NDUFB4), [COX8A](https://www.affymetrix.com/LinkServlet?probeset=COX8A), [NDUFA7](https://www.affymetrix.com/LinkServlet?probeset=NDUFA7), [ATP6V1G2](https://www.affymetrix.com/LinkServlet?probeset=ATP6V1G2), [COX4I1](https://www.affymetrix.com/LinkServlet?probeset=COX4I1), [NDUFA3](https://www.affymetrix.com/LinkServlet?probeset=NDUFA3), [NDUFS6](https://www.affymetrix.com/LinkServlet?probeset=NDUFS6), [NDUFC1](https://www.affymetrix.com/LinkServlet?probeset=NDUFC1), [ATP5J2](https://www.affymetrix.com/LinkServlet?probeset=ATP5J2), [COX5A](https://www.affymetrix.com/LinkServlet?probeset=COX5A), [ATP5I](https://www.affymetrix.com/LinkServlet?probeset=ATP5I), [COX6A2](https://www.affymetrix.com/LinkServlet?probeset=COX6A2), [UQCR11](https://www.affymetrix.com/LinkServlet?probeset=UQCR11), [COX6A1](https://www.affymetrix.com/LinkServlet?probeset=COX6A1), [ATP5G1](https://www.affymetrix.com/LinkServlet?probeset=ATP5G1), [COX7C](https://www.affymetrix.com/LinkServlet?probeset=COX7C), [UQCRQ](https://www.affymetrix.com/LinkServlet?probeset=UQCRQ), [NDUFS4](https://www.affymetrix.com/LinkServlet?probeset=NDUFS4), and [LHPP](https://www.affymetrix.com/LinkServlet?probeset=LHPP) |
| Maturity onset diabetes of the young | -2.02E+00 | 0.00E+00 | 2.89E-03 | [NEUROD1](https://www.affymetrix.com/LinkServlet?probeset=NEUROD1), [IAPP](https://www.affymetrix.com/LinkServlet?probeset=IAPP), [PAX4](https://www.affymetrix.com/LinkServlet?probeset=PAX4), [PDX1](https://www.affymetrix.com/LinkServlet?probeset=PDX1), [FOXA2](https://www.affymetrix.com/LinkServlet?probeset=FOXA2), [PAX6](https://www.affymetrix.com/LinkServlet?probeset=PAX6), [INS](https://www.affymetrix.com/LinkServlet?probeset=INS), [HNF1A](https://www.affymetrix.com/LinkServlet?probeset=HNF1A), [NKX6-1](https://www.affymetrix.com/LinkServlet?probeset=NKX6-1), [HNF4A](https://www.affymetrix.com/LinkServlet?probeset=HNF4A), and [HNF4G](https://www.affymetrix.com/LinkServlet?probeset=HNF4G) |
| Autoimmune thyroid disease | -1.72E+00 | 0.00E+00 | 7.25E-02 | [FASLG](https://www.affymetrix.com/LinkServlet?probeset=FASLG), [IFNA17](https://www.affymetrix.com/LinkServlet?probeset=IFNA17), [IL4](https://www.affymetrix.com/LinkServlet?probeset=IL4), [HLA-DOA](https://www.affymetrix.com/LinkServlet?probeset=HLA-DOA), [CGA](https://www.affymetrix.com/LinkServlet?probeset=CGA), [IFNA8](https://www.affymetrix.com/LinkServlet?probeset=IFNA8), [IL10](https://www.affymetrix.com/LinkServlet?probeset=IL10), [IFNA1](https://www.affymetrix.com/LinkServlet?probeset=IFNA1), [IFNA14](https://www.affymetrix.com/LinkServlet?probeset=IFNA14), [IFNA7](https://www.affymetrix.com/LinkServlet?probeset=IFNA7), [CD40LG](https://www.affymetrix.com/LinkServlet?probeset=CD40LG), [TPO](https://www.affymetrix.com/LinkServlet?probeset=TPO), [IL5](https://www.affymetrix.com/LinkServlet?probeset=IL5), [IL2](https://www.affymetrix.com/LinkServlet?probeset=IL2), [IFNA4](https://www.affymetrix.com/LinkServlet?probeset=IFNA4), [TSHB](https://www.affymetrix.com/LinkServlet?probeset=TSHB), [IFNA21](https://www.affymetrix.com/LinkServlet?probeset=IFNA21), [IFNA2](https://www.affymetrix.com/LinkServlet?probeset=IFNA2), [TG](https://www.affymetrix.com/LinkServlet?probeset=TG), [IFNA5](https://www.affymetrix.com/LinkServlet?probeset=IFNA5), and [IFNA16](https://www.affymetrix.com/LinkServlet?probeset=IFNA16) |
| Calcium signaling pathway | -1.57E+00 | 2.79E-03 | 1.29E-01 | [GNAL](https://www.affymetrix.com/LinkServlet?probeset=GNAL), [CACNA1C](https://www.affymetrix.com/LinkServlet?probeset=CACNA1C), [P2RX3](https://www.affymetrix.com/LinkServlet?probeset=P2RX3), [GRIN1](https://www.affymetrix.com/LinkServlet?probeset=GRIN1), [PLCE1](https://www.affymetrix.com/LinkServlet?probeset=PLCE1), [ATP2A3](https://www.affymetrix.com/LinkServlet?probeset=ATP2A3), [CACNA1B](https://www.affymetrix.com/LinkServlet?probeset=CACNA1B), [PDE1C](https://www.affymetrix.com/LinkServlet?probeset=PDE1C), [RYR3](https://www.affymetrix.com/LinkServlet?probeset=RYR3), [DRD5](https://www.affymetrix.com/LinkServlet?probeset=DRD5), [MYLK3](https://www.affymetrix.com/LinkServlet?probeset=MYLK3), [HTR5A](https://www.affymetrix.com/LinkServlet?probeset=HTR5A), [PHKA1](https://www.affymetrix.com/LinkServlet?probeset=PHKA1), [NTSR1](https://www.affymetrix.com/LinkServlet?probeset=NTSR1), [NOS2](https://www.affymetrix.com/LinkServlet?probeset=NOS2), [ADORA2B](https://www.affymetrix.com/LinkServlet?probeset=ADORA2B), [RYR2](https://www.affymetrix.com/LinkServlet?probeset=RYR2), [CALML5](https://www.affymetrix.com/LinkServlet?probeset=CALML5), [ATP2B2](https://www.affymetrix.com/LinkServlet?probeset=ATP2B2), [PDE1B](https://www.affymetrix.com/LinkServlet?probeset=PDE1B), [AVPR1B](https://www.affymetrix.com/LinkServlet?probeset=AVPR1B), [CACNA1H](https://www.affymetrix.com/LinkServlet?probeset=CACNA1H), [ADCY3](https://www.affymetrix.com/LinkServlet?probeset=ADCY3), [CAMK2B](https://www.affymetrix.com/LinkServlet?probeset=CAMK2B), [GRM5](https://www.affymetrix.com/LinkServlet?probeset=GRM5), [ADRB3](https://www.affymetrix.com/LinkServlet?probeset=ADRB3), [PLCD1](https://www.affymetrix.com/LinkServlet?probeset=PLCD1), [PLCG1](https://www.affymetrix.com/LinkServlet?probeset=PLCG1), [DRD1](https://www.affymetrix.com/LinkServlet?probeset=DRD1), [CCKBR](https://www.affymetrix.com/LinkServlet?probeset=CCKBR), [TNNC2](https://www.affymetrix.com/LinkServlet?probeset=TNNC2), [PHKG1](https://www.affymetrix.com/LinkServlet?probeset=PHKG1), [ADRA1D](https://www.affymetrix.com/LinkServlet?probeset=ADRA1D), [GRIN2D](https://www.affymetrix.com/LinkServlet?probeset=GRIN2D), [TACR3](https://www.affymetrix.com/LinkServlet?probeset=TACR3), [CACNA1A](https://www.affymetrix.com/LinkServlet?probeset=CACNA1A), [PDGFRB](https://www.affymetrix.com/LinkServlet?probeset=PDGFRB), [CACNA1G](https://www.affymetrix.com/LinkServlet?probeset=CACNA1G), [P2RX2](https://www.affymetrix.com/LinkServlet?probeset=P2RX2), [CHP2](https://www.affymetrix.com/LinkServlet?probeset=CHP2), [RYR1](https://www.affymetrix.com/LinkServlet?probeset=RYR1), [LTB4R2](https://www.affymetrix.com/LinkServlet?probeset=LTB4R2), [HTR6](https://www.affymetrix.com/LinkServlet?probeset=HTR6), [GRIN2C](https://www.affymetrix.com/LinkServlet?probeset=GRIN2C), [PHKG2](https://www.affymetrix.com/LinkServlet?probeset=PHKG2), [TNNC1](https://www.affymetrix.com/LinkServlet?probeset=TNNC1), [HTR2A](https://www.affymetrix.com/LinkServlet?probeset=HTR2A), [SLC8A2](https://www.affymetrix.com/LinkServlet?probeset=SLC8A2), [CAMK2A](https://www.affymetrix.com/LinkServlet?probeset=CAMK2A), [PTAFR](https://www.affymetrix.com/LinkServlet?probeset=PTAFR), [ATP2B3](https://www.affymetrix.com/LinkServlet?probeset=ATP2B3), [NOS1](https://www.affymetrix.com/LinkServlet?probeset=NOS1), [GRIN2A](https://www.affymetrix.com/LinkServlet?probeset=GRIN2A), [ADCY1](https://www.affymetrix.com/LinkServlet?probeset=ADCY1), [CACNA1I](https://www.affymetrix.com/LinkServlet?probeset=CACNA1I), [PRKACG](https://www.affymetrix.com/LinkServlet?probeset=PRKACG), [GRPR](https://www.affymetrix.com/LinkServlet?probeset=GRPR), [CALML3](https://www.affymetrix.com/LinkServlet?probeset=CALML3), [TBXA2R](https://www.affymetrix.com/LinkServlet?probeset=TBXA2R), [TRHR](https://www.affymetrix.com/LinkServlet?probeset=TRHR), [GRM1](https://www.affymetrix.com/LinkServlet?probeset=GRM1), [CHRM5](https://www.affymetrix.com/LinkServlet?probeset=CHRM5), [HTR4](https://www.affymetrix.com/LinkServlet?probeset=HTR4), [PRKACA](https://www.affymetrix.com/LinkServlet?probeset=PRKACA), [CAMK4](https://www.affymetrix.com/LinkServlet?probeset=CAMK4), [PTK2B](https://www.affymetrix.com/LinkServlet?probeset=PTK2B), [P2RX6](https://www.affymetrix.com/LinkServlet?probeset=P2RX6), [CCKAR](https://www.affymetrix.com/LinkServlet?probeset=CCKAR), [ADCY8](https://www.affymetrix.com/LinkServlet?probeset=ADCY8), [TACR2](https://www.affymetrix.com/LinkServlet?probeset=TACR2), [TACR1](https://www.affymetrix.com/LinkServlet?probeset=TACR1), [ADRB1](https://www.affymetrix.com/LinkServlet?probeset=ADRB1), [SPHK2](https://www.affymetrix.com/LinkServlet?probeset=SPHK2), and [CHRM2](https://www.affymetrix.com/LinkServlet?probeset=CHRM2) |
| Alzheimer’s disease | -1.59E+00 | 2.85E-03 | 1.22E-01 | [CALML5](https://www.affymetrix.com/LinkServlet?probeset=CALML5), [CAPN2](https://www.affymetrix.com/LinkServlet?probeset=CAPN2), [NDUFB3](https://www.affymetrix.com/LinkServlet?probeset=NDUFB3), [NDUFA9](https://www.affymetrix.com/LinkServlet?probeset=NDUFA9), [NDUFB2](https://www.affymetrix.com/LinkServlet?probeset=NDUFB2), [UQCRC1](https://www.affymetrix.com/LinkServlet?probeset=UQCRC1), [CDK5R1](https://www.affymetrix.com/LinkServlet?probeset=CDK5R1), [COX5B](https://www.affymetrix.com/LinkServlet?probeset=COX5B), [CDK5](https://www.affymetrix.com/LinkServlet?probeset=CDK5), [NDUFA4L2](https://www.affymetrix.com/LinkServlet?probeset=NDUFA4L2), [COX7A2L](https://www.affymetrix.com/LinkServlet?probeset=COX7A2L), [NDUFAB1](https://www.affymetrix.com/LinkServlet?probeset=NDUFAB1), [NDUFA8](https://www.affymetrix.com/LinkServlet?probeset=NDUFA8), [APOE](https://www.affymetrix.com/LinkServlet?probeset=APOE), [GRIN2D](https://www.affymetrix.com/LinkServlet?probeset=GRIN2D), [FADD](https://www.affymetrix.com/LinkServlet?probeset=FADD), [COX7A1](https://www.affymetrix.com/LinkServlet?probeset=COX7A1), [ATP5E](https://www.affymetrix.com/LinkServlet?probeset=ATP5E), [NDUFA1](https://www.affymetrix.com/LinkServlet?probeset=NDUFA1), [CHP2](https://www.affymetrix.com/LinkServlet?probeset=CHP2), [MAPT](https://www.affymetrix.com/LinkServlet?probeset=MAPT), [ATP5H](https://www.affymetrix.com/LinkServlet?probeset=ATP5H), [PSEN2](https://www.affymetrix.com/LinkServlet?probeset=PSEN2), [NDUFA2](https://www.affymetrix.com/LinkServlet?probeset=NDUFA2), [MME](https://www.affymetrix.com/LinkServlet?probeset=MME), [NDUFB8](https://www.affymetrix.com/LinkServlet?probeset=NDUFB8), [ATP5J](https://www.affymetrix.com/LinkServlet?probeset=ATP5J), [CYC1](https://www.affymetrix.com/LinkServlet?probeset=CYC1), [HSD17B10](https://www.affymetrix.com/LinkServlet?probeset=HSD17B10), [COX7B](https://www.affymetrix.com/LinkServlet?probeset=COX7B), [GRIN2C](https://www.affymetrix.com/LinkServlet?probeset=GRIN2C), [ATP5D](https://www.affymetrix.com/LinkServlet?probeset=ATP5D), [ERN1](https://www.affymetrix.com/LinkServlet?probeset=ERN1), [NDUFB4](https://www.affymetrix.com/LinkServlet?probeset=NDUFB4), [COX8A](https://www.affymetrix.com/LinkServlet?probeset=COX8A), [NDUFA7](https://www.affymetrix.com/LinkServlet?probeset=NDUFA7), [NOS1](https://www.affymetrix.com/LinkServlet?probeset=NOS1), [LPL](https://www.affymetrix.com/LinkServlet?probeset=LPL), [COX4I1](https://www.affymetrix.com/LinkServlet?probeset=COX4I1), [APBB1](https://www.affymetrix.com/LinkServlet?probeset=APBB1), [NDUFA3](https://www.affymetrix.com/LinkServlet?probeset=NDUFA3), [GRIN2A](https://www.affymetrix.com/LinkServlet?probeset=GRIN2A), [NDUFS6](https://www.affymetrix.com/LinkServlet?probeset=NDUFS6), [CALML3](https://www.affymetrix.com/LinkServlet?probeset=CALML3), [NDUFC1](https://www.affymetrix.com/LinkServlet?probeset=NDUFC1), [COX5A](https://www.affymetrix.com/LinkServlet?probeset=COX5A), [COX6A2](https://www.affymetrix.com/LinkServlet?probeset=COX6A2), [MAPK3](https://www.affymetrix.com/LinkServlet?probeset=MAPK3), [UQCR11](https://www.affymetrix.com/LinkServlet?probeset=UQCR11), [COX6A1](https://www.affymetrix.com/LinkServlet?probeset=COX6A1), [ATP5G1](https://www.affymetrix.com/LinkServlet?probeset=ATP5G1), [COX7C](https://www.affymetrix.com/LinkServlet?probeset=COX7C), [UQCRQ](https://www.affymetrix.com/LinkServlet?probeset=UQCRQ), and [NDUFS4](https://www.affymetrix.com/LinkServlet?probeset=NDUFS4) |
| Cardiac muscle contraction | -1.67E+00 | 4.53E-03 | 9.89E-02 | [FXYD2](https://www.affymetrix.com/LinkServlet?probeset=FXYD2), [COX6C](https://www.affymetrix.com/LinkServlet?probeset=COX6C), [CACNB4](https://www.affymetrix.com/LinkServlet?probeset=CACNB4), [COX6B1](https://www.affymetrix.com/LinkServlet?probeset=COX6B1), [SLC9A1](https://www.affymetrix.com/LinkServlet?probeset=SLC9A1), [CACNA1C](https://www.affymetrix.com/LinkServlet?probeset=CACNA1C), [MYH7](https://www.affymetrix.com/LinkServlet?probeset=MYH7), [CACNB2](https://www.affymetrix.com/LinkServlet?probeset=CACNB2), [RYR2](https://www.affymetrix.com/LinkServlet?probeset=RYR2), [CACNG4](https://www.affymetrix.com/LinkServlet?probeset=CACNG4), [UQCRC1](https://www.affymetrix.com/LinkServlet?probeset=UQCRC1), [COX5B](https://www.affymetrix.com/LinkServlet?probeset=COX5B), [COX7A2L](https://www.affymetrix.com/LinkServlet?probeset=COX7A2L), [ATP1A2](https://www.affymetrix.com/LinkServlet?probeset=ATP1A2), [COX7A1](https://www.affymetrix.com/LinkServlet?probeset=COX7A1), [TNNT2](https://www.affymetrix.com/LinkServlet?probeset=TNNT2), [MYL3](https://www.affymetrix.com/LinkServlet?probeset=MYL3), [CYC1](https://www.affymetrix.com/LinkServlet?probeset=CYC1), [COX7B](https://www.affymetrix.com/LinkServlet?probeset=COX7B), [COX8A](https://www.affymetrix.com/LinkServlet?probeset=COX8A), [TNNC1](https://www.affymetrix.com/LinkServlet?probeset=TNNC1), [COX4I1](https://www.affymetrix.com/LinkServlet?probeset=COX4I1), [ATP1A3](https://www.affymetrix.com/LinkServlet?probeset=ATP1A3), [CACNB1](https://www.affymetrix.com/LinkServlet?probeset=CACNB1), [COX5A](https://www.affymetrix.com/LinkServlet?probeset=COX5A), [COX6A2](https://www.affymetrix.com/LinkServlet?probeset=COX6A2), [CACNG5](https://www.affymetrix.com/LinkServlet?probeset=CACNG5), [UQCR11](https://www.affymetrix.com/LinkServlet?probeset=UQCR11), [ATP1B2](https://www.affymetrix.com/LinkServlet?probeset=ATP1B2), [COX6A1](https://www.affymetrix.com/LinkServlet?probeset=COX6A1), [COX7C](https://www.affymetrix.com/LinkServlet?probeset=COX7C), and [UQCRQ](https://www.affymetrix.com/LinkServlet?probeset=UQCRQ) |
| Arachidonic acid metabolism | -1.62E+00 | 4.89E-03 | 1.18E-01 | [PLA2G12A](https://www.affymetrix.com/LinkServlet?probeset=PLA2G12A), [GPX3](https://www.affymetrix.com/LinkServlet?probeset=GPX3), [CYP4F3](https://www.affymetrix.com/LinkServlet?probeset=CYP4F3), [PLA2G2A](https://www.affymetrix.com/LinkServlet?probeset=PLA2G2A), [PLA2G2F](https://www.affymetrix.com/LinkServlet?probeset=PLA2G2F), [GPX4](https://www.affymetrix.com/LinkServlet?probeset=GPX4), [LTC4S](https://www.affymetrix.com/LinkServlet?probeset=LTC4S), [PLA2G1B](https://www.affymetrix.com/LinkServlet?probeset=PLA2G1B), [PTGES2](https://www.affymetrix.com/LinkServlet?probeset=PTGES2), [PLA2G6](https://www.affymetrix.com/LinkServlet?probeset=PLA2G6), [PLA2G3](https://www.affymetrix.com/LinkServlet?probeset=PLA2G3), [ALOX12](https://www.affymetrix.com/LinkServlet?probeset=ALOX12), [CYP4A11](https://www.affymetrix.com/LinkServlet?probeset=CYP4A11), [ALOX15](https://www.affymetrix.com/LinkServlet?probeset=ALOX15), [CYP4A22](https://www.affymetrix.com/LinkServlet?probeset=CYP4A22), [CYP2C8](https://www.affymetrix.com/LinkServlet?probeset=CYP2C8), [ALOX12B](https://www.affymetrix.com/LinkServlet?probeset=ALOX12B), [CYP2J2](https://www.affymetrix.com/LinkServlet?probeset=CYP2J2), [CYP2B6](https://www.affymetrix.com/LinkServlet?probeset=CYP2B6), [PLA2G5](https://www.affymetrix.com/LinkServlet?probeset=PLA2G5), [ALOX15B](https://www.affymetrix.com/LinkServlet?probeset=ALOX15B), [PLA2G2D](https://www.affymetrix.com/LinkServlet?probeset=PLA2G2D), [CYP2C9](https://www.affymetrix.com/LinkServlet?probeset=CYP2C9), [CYP2C19](https://www.affymetrix.com/LinkServlet?probeset=CYP2C19), [PTGDS](https://www.affymetrix.com/LinkServlet?probeset=PTGDS), [GPX2](https://www.affymetrix.com/LinkServlet?probeset=GPX2), and [GPX5](https://www.affymetrix.com/LinkServlet?probeset=GPX5) |
| Ribosome | -1.51E+00 | 1.41E-02 | 1.69E-01 | [RPL10](https://www.affymetrix.com/LinkServlet?probeset=RPL10), [RPS19](https://www.affymetrix.com/LinkServlet?probeset=RPS19), [RPS24](https://www.affymetrix.com/LinkServlet?probeset=RPS24), [RPL27](https://www.affymetrix.com/LinkServlet?probeset=RPL27), [RPL14](https://www.affymetrix.com/LinkServlet?probeset=RPL14), [RPL15](https://www.affymetrix.com/LinkServlet?probeset=RPL15), [RPL35](https://www.affymetrix.com/LinkServlet?probeset=RPL35), [RPL35A](https://www.affymetrix.com/LinkServlet?probeset=RPL35A), [RPLP1](https://www.affymetrix.com/LinkServlet?probeset=RPLP1), [RPL38](https://www.affymetrix.com/LinkServlet?probeset=RPL38), [RPL31](https://www.affymetrix.com/LinkServlet?probeset=RPL31), [RPS29](https://www.affymetrix.com/LinkServlet?probeset=RPS29), [FAU](https://www.affymetrix.com/LinkServlet?probeset=FAU), [RPS4Y1](https://www.affymetrix.com/LinkServlet?probeset=RPS4Y1), [RPL3](https://www.affymetrix.com/LinkServlet?probeset=RPL3), [RPS21](https://www.affymetrix.com/LinkServlet?probeset=RPS21), [RPL27A](https://www.affymetrix.com/LinkServlet?probeset=RPL27A), [RPS26](https://www.affymetrix.com/LinkServlet?probeset=RPS26), [RPL41](https://www.affymetrix.com/LinkServlet?probeset=RPL41), [RPL29](https://www.affymetrix.com/LinkServlet?probeset=RPL29), [RPS12](https://www.affymetrix.com/LinkServlet?probeset=RPS12), [RPL36](https://www.affymetrix.com/LinkServlet?probeset=RPL36), [RPL19](https://www.affymetrix.com/LinkServlet?probeset=RPL19), [UBA52](https://www.affymetrix.com/LinkServlet?probeset=UBA52), [RPS11](https://www.affymetrix.com/LinkServlet?probeset=RPS11), [RPS10](https://www.affymetrix.com/LinkServlet?probeset=RPS10), [RPS27](https://www.affymetrix.com/LinkServlet?probeset=RPS27), [RPL30](https://www.affymetrix.com/LinkServlet?probeset=RPL30), [RPL9](https://www.affymetrix.com/LinkServlet?probeset=RPL9), [RPS2](https://www.affymetrix.com/LinkServlet?probeset=RPS2), [RPLP2](https://www.affymetrix.com/LinkServlet?probeset=RPLP2), [RPS28](https://www.affymetrix.com/LinkServlet?probeset=RPS28), [RPS27A](https://www.affymetrix.com/LinkServlet?probeset=RPS27A), [RPL32](https://www.affymetrix.com/LinkServlet?probeset=RPL32), [RPS15](https://www.affymetrix.com/LinkServlet?probeset=RPS15), [RPL10A](https://www.affymetrix.com/LinkServlet?probeset=RPL10A), [RPS23](https://www.affymetrix.com/LinkServlet?probeset=RPS23), [RPL26L1](https://www.affymetrix.com/LinkServlet?probeset=RPL26L1), [RPS18](https://www.affymetrix.com/LinkServlet?probeset=RPS18), [RPS18](https://www.affymetrix.com/LinkServlet?probeset=RPS18), [RPL10L](https://www.affymetrix.com/LinkServlet?probeset=RPL10L), [RPL23A](https://www.affymetrix.com/LinkServlet?probeset=RPL23A), and [RPS27L](https://www.affymetrix.com/LinkServlet?probeset=RPS27L) |
| Parkinson’s disease | -1.51E+00 | 1.51E-02 | 1.64E-01 | [PINK1](https://www.affymetrix.com/LinkServlet?probeset=PINK1), [NDUFB7](https://www.affymetrix.com/LinkServlet?probeset=NDUFB7), [COX6C](https://www.affymetrix.com/LinkServlet?probeset=COX6C), [NDUFV2](https://www.affymetrix.com/LinkServlet?probeset=NDUFV2), [NDUFS5](https://www.affymetrix.com/LinkServlet?probeset=NDUFS5), [COX6B1](https://www.affymetrix.com/LinkServlet?probeset=COX6B1), [NDUFS2](https://www.affymetrix.com/LinkServlet?probeset=NDUFS2), [NDUFS1](https://www.affymetrix.com/LinkServlet?probeset=NDUFS1), [SLC18A1](https://www.affymetrix.com/LinkServlet?probeset=SLC18A1), [TH](https://www.affymetrix.com/LinkServlet?probeset=TH), [NDUFB3](https://www.affymetrix.com/LinkServlet?probeset=NDUFB3), [PARK2](https://www.affymetrix.com/LinkServlet?probeset=PARK2), [NDUFA9](https://www.affymetrix.com/LinkServlet?probeset=NDUFA9), [NDUFB2](https://www.affymetrix.com/LinkServlet?probeset=NDUFB2), [UQCRC1](https://www.affymetrix.com/LinkServlet?probeset=UQCRC1), [COX5B](https://www.affymetrix.com/LinkServlet?probeset=COX5B), [NDUFA4L2](https://www.affymetrix.com/LinkServlet?probeset=NDUFA4L2), [COX7A2L](https://www.affymetrix.com/LinkServlet?probeset=COX7A2L), [NDUFAB1](https://www.affymetrix.com/LinkServlet?probeset=NDUFAB1), [NDUFA8](https://www.affymetrix.com/LinkServlet?probeset=NDUFA8), [COX7A1](https://www.affymetrix.com/LinkServlet?probeset=COX7A1), [ATP5E](https://www.affymetrix.com/LinkServlet?probeset=ATP5E), [NDUFA1](https://www.affymetrix.com/LinkServlet?probeset=NDUFA1), [ATP5H](https://www.affymetrix.com/LinkServlet?probeset=ATP5H), [NDUFA2](https://www.affymetrix.com/LinkServlet?probeset=NDUFA2), [NDUFB8](https://www.affymetrix.com/LinkServlet?probeset=NDUFB8), [ATP5J](https://www.affymetrix.com/LinkServlet?probeset=ATP5J), [CYC1](https://www.affymetrix.com/LinkServlet?probeset=CYC1), [COX7B](https://www.affymetrix.com/LinkServlet?probeset=COX7B), [ATP5D](https://www.affymetrix.com/LinkServlet?probeset=ATP5D), [NDUFB4](https://www.affymetrix.com/LinkServlet?probeset=NDUFB4), [COX8A](https://www.affymetrix.com/LinkServlet?probeset=COX8A), [NDUFA7](https://www.affymetrix.com/LinkServlet?probeset=NDUFA7), [COX4I1](https://www.affymetrix.com/LinkServlet?probeset=COX4I1), [NDUFA3](https://www.affymetrix.com/LinkServlet?probeset=NDUFA3), [NDUFS6](https://www.affymetrix.com/LinkServlet?probeset=NDUFS6), [UBB](https://www.affymetrix.com/LinkServlet?probeset=UBB), [NDUFC1](https://www.affymetrix.com/LinkServlet?probeset=NDUFC1), [COX5A](https://www.affymetrix.com/LinkServlet?probeset=COX5A), [COX6A2](https://www.affymetrix.com/LinkServlet?probeset=COX6A2), [UQCR11](https://www.affymetrix.com/LinkServlet?probeset=UQCR11), [COX6A1](https://www.affymetrix.com/LinkServlet?probeset=COX6A1), [ATP5G1](https://www.affymetrix.com/LinkServlet?probeset=ATP5G1), [COX7C](https://www.affymetrix.com/LinkServlet?probeset=COX7C), [UQCRQ](https://www.affymetrix.com/LinkServlet?probeset=UQCRQ), and [NDUFS4](https://www.affymetrix.com/LinkServlet?probeset=NDUFS4) |
| Linoleic acid metabolism | -1.60E+00 | 1.17E-02 | 1.29E-01 | [CYP1A2](https://www.affymetrix.com/LinkServlet?probeset=CYP1A2), [PLA2G12A](https://www.affymetrix.com/LinkServlet?probeset=PLA2G12A), [PLA2G2A](https://www.affymetrix.com/LinkServlet?probeset=PLA2G2A), [PLA2G2F](https://www.affymetrix.com/LinkServlet?probeset=PLA2G2F), [PLA2G1B](https://www.affymetrix.com/LinkServlet?probeset=PLA2G1B), [PLA2G6](https://www.affymetrix.com/LinkServlet?probeset=PLA2G6), [PLA2G3](https://www.affymetrix.com/LinkServlet?probeset=PLA2G3), [ALOX15](https://www.affymetrix.com/LinkServlet?probeset=ALOX15), [CYP2C8](https://www.affymetrix.com/LinkServlet?probeset=CYP2C8), [CYP2J2](https://www.affymetrix.com/LinkServlet?probeset=CYP2J2), [PLA2G5](https://www.affymetrix.com/LinkServlet?probeset=PLA2G5), [PLA2G2D](https://www.affymetrix.com/LinkServlet?probeset=PLA2G2D), [CYP2C9](https://www.affymetrix.com/LinkServlet?probeset=CYP2C9), [CYP2C19](https://www.affymetrix.com/LinkServlet?probeset=CYP2C19), and [CYP3A43](https://www.affymetrix.com/LinkServlet?probeset=CYP3A43) |
| Peroxisome | -1.55E+00 | 1.83E-02 | 1.45E-01 | [DAO](https://www.affymetrix.com/LinkServlet?probeset=DAO), [PEX19](https://www.affymetrix.com/LinkServlet?probeset=PEX19), [IDH1](https://www.affymetrix.com/LinkServlet?probeset=IDH1), [SOD1](https://www.affymetrix.com/LinkServlet?probeset=SOD1), [NOS2](https://www.affymetrix.com/LinkServlet?probeset=NOS2), [HAO2](https://www.affymetrix.com/LinkServlet?probeset=HAO2), [XDH](https://www.affymetrix.com/LinkServlet?probeset=XDH), [PEX14](https://www.affymetrix.com/LinkServlet?probeset=PEX14), [ACOX2](https://www.affymetrix.com/LinkServlet?probeset=ACOX2), [PIPOX](https://www.affymetrix.com/LinkServlet?probeset=PIPOX), [MPV17](https://www.affymetrix.com/LinkServlet?probeset=MPV17), [PEX26](https://www.affymetrix.com/LinkServlet?probeset=PEX26), [RNASEH2A](https://www.affymetrix.com/LinkServlet?probeset=RNASEH2A), [ACAA1](https://www.affymetrix.com/LinkServlet?probeset=ACAA1), [PXMP4](https://www.affymetrix.com/LinkServlet?probeset=PXMP4), [AGXT](https://www.affymetrix.com/LinkServlet?probeset=AGXT), [MVK](https://www.affymetrix.com/LinkServlet?probeset=MVK), [BAAT](https://www.affymetrix.com/LinkServlet?probeset=BAAT), [AMACR](https://www.affymetrix.com/LinkServlet?probeset=AMACR), [PAOX](https://www.affymetrix.com/LinkServlet?probeset=PAOX), [HMGCL](https://www.affymetrix.com/LinkServlet?probeset=HMGCL), [CRAT](https://www.affymetrix.com/LinkServlet?probeset=CRAT), [PEX10](https://www.affymetrix.com/LinkServlet?probeset=PEX10), [MLYCD](https://www.affymetrix.com/LinkServlet?probeset=MLYCD), [PXMP2](https://www.affymetrix.com/LinkServlet?probeset=PXMP2), [DECR2](https://www.affymetrix.com/LinkServlet?probeset=DECR2), [ACOT8](https://www.affymetrix.com/LinkServlet?probeset=ACOT8), and [ABCD1](https://www.affymetrix.com/LinkServlet?probeset=ABCD1) |
| MTOR signaling pathway | -1.54E+00 | 2.05E-02 | 1.48E-01 | [DDIT4](https://www.affymetrix.com/LinkServlet?probeset=DDIT4), [PIK3R2](https://www.affymetrix.com/LinkServlet?probeset=PIK3R2), [AKT2](https://www.affymetrix.com/LinkServlet?probeset=AKT2), [MLST8](https://www.affymetrix.com/LinkServlet?probeset=MLST8), [EIF4EBP1](https://www.affymetrix.com/LinkServlet?probeset=EIF4EBP1), [PIK3R5](https://www.affymetrix.com/LinkServlet?probeset=PIK3R5), [RPS6KB2](https://www.affymetrix.com/LinkServlet?probeset=RPS6KB2), [STK11](https://www.affymetrix.com/LinkServlet?probeset=STK11), [RPS6KA6](https://www.affymetrix.com/LinkServlet?probeset=RPS6KA6), [VEGFA](https://www.affymetrix.com/LinkServlet?probeset=VEGFA), [STRADA](https://www.affymetrix.com/LinkServlet?probeset=STRADA), [INS](https://www.affymetrix.com/LinkServlet?probeset=INS), [MAPK3](https://www.affymetrix.com/LinkServlet?probeset=MAPK3), [MTOR](https://www.affymetrix.com/LinkServlet?probeset=MTOR), and [EIF4B](https://www.affymetrix.com/LinkServlet?probeset=EIF4B) |
| Glycosphingolipid biosynthesis lacto and neolacto series | -1.63E+00 | 2.16E-02 | 1.31E-01 | [FUT1](https://www.affymetrix.com/LinkServlet?probeset=FUT1), [B3GNT4](https://www.affymetrix.com/LinkServlet?probeset=B3GNT4), [B3GALT5](https://www.affymetrix.com/LinkServlet?probeset=B3GALT5), [GCNT2](https://www.affymetrix.com/LinkServlet?probeset=GCNT2), [B3GALT1](https://www.affymetrix.com/LinkServlet?probeset=B3GALT1), [ST3GAL6](https://www.affymetrix.com/LinkServlet?probeset=ST3GAL6), [B4GALT3](https://www.affymetrix.com/LinkServlet?probeset=B4GALT3), [ABO](https://www.affymetrix.com/LinkServlet?probeset=ABO), [FUT6](https://www.affymetrix.com/LinkServlet?probeset=FUT6), [FUT5](https://www.affymetrix.com/LinkServlet?probeset=FUT5), [FUT7](https://www.affymetrix.com/LinkServlet?probeset=FUT7), [B3GALT2](https://www.affymetrix.com/LinkServlet?probeset=B3GALT2), [B4GALT2](https://www.affymetrix.com/LinkServlet?probeset=B4GALT2), [ST3GAL4](https://www.affymetrix.com/LinkServlet?probeset=ST3GAL4), [B3GNT3](https://www.affymetrix.com/LinkServlet?probeset=B3GNT3), [FUT3](https://www.affymetrix.com/LinkServlet?probeset=FUT3), and [B3GNT1](https://www.affymetrix.com/LinkServlet?probeset=B3GNT1) |
| JAK/STAT signaling pathway | -1.45E+00 | 2.45E-02 | 2.15E-01 | [IL12B](https://www.affymetrix.com/LinkServlet?probeset=IL12B), [STAT2](https://www.affymetrix.com/LinkServlet?probeset=STAT2), IL3RA, [IFNA17](https://www.affymetrix.com/LinkServlet?probeset=IFNA17), [CNTF](https://www.affymetrix.com/LinkServlet?probeset=CNTF), [PIK3CB](https://www.affymetrix.com/LinkServlet?probeset=PIK3CB), [LEP](https://www.affymetrix.com/LinkServlet?probeset=LEP), [IL4](https://www.affymetrix.com/LinkServlet?probeset=IL4), [SOCS7](https://www.affymetrix.com/LinkServlet?probeset=SOCS7), [IFNA8](https://www.affymetrix.com/LinkServlet?probeset=IFNA8), [CNTFR](https://www.affymetrix.com/LinkServlet?probeset=CNTFR), [PIAS3](https://www.affymetrix.com/LinkServlet?probeset=PIAS3), [TYK2](https://www.affymetrix.com/LinkServlet?probeset=TYK2), [MPL](https://www.affymetrix.com/LinkServlet?probeset=MPL), [IL12RB1](https://www.affymetrix.com/LinkServlet?probeset=IL12RB1), [IL10](https://www.affymetrix.com/LinkServlet?probeset=IL10), [IL11RA](https://www.affymetrix.com/LinkServlet?probeset=IL11RA), [CISH](https://www.affymetrix.com/LinkServlet?probeset=CISH), [IFNA1](https://www.affymetrix.com/LinkServlet?probeset=IFNA1), [IFNA14](https://www.affymetrix.com/LinkServlet?probeset=IFNA14), [IL23A](https://www.affymetrix.com/LinkServlet?probeset=IL23A), [EPOR](https://www.affymetrix.com/LinkServlet?probeset=EPOR), [IFNA7](https://www.affymetrix.com/LinkServlet?probeset=IFNA7), [IL2RG](https://www.affymetrix.com/LinkServlet?probeset=IL2RG), [IL9](https://www.affymetrix.com/LinkServlet?probeset=IL9), [CBL](https://www.affymetrix.com/LinkServlet?probeset=CBL), [IFNB1](https://www.affymetrix.com/LinkServlet?probeset=IFNB1), [SOS1](https://www.affymetrix.com/LinkServlet?probeset=SOS1), [CRLF2](https://www.affymetrix.com/LinkServlet?probeset=CRLF2), [IL24](https://www.affymetrix.com/LinkServlet?probeset=IL24), [JAK3](https://www.affymetrix.com/LinkServlet?probeset=JAK3), [PIK3R2](https://www.affymetrix.com/LinkServlet?probeset=PIK3R2), [AKT2](https://www.affymetrix.com/LinkServlet?probeset=AKT2), [IL3](https://www.affymetrix.com/LinkServlet?probeset=IL3), [TPO](https://www.affymetrix.com/LinkServlet?probeset=TPO), [PRL](https://www.affymetrix.com/LinkServlet?probeset=PRL), [CSF2](https://www.affymetrix.com/LinkServlet?probeset=CSF2), [IL13RA2](https://www.affymetrix.com/LinkServlet?probeset=IL13RA2), [IL5](https://www.affymetrix.com/LinkServlet?probeset=IL5), [PIK3R5](https://www.affymetrix.com/LinkServlet?probeset=PIK3R5), [IL2](https://www.affymetrix.com/LinkServlet?probeset=IL2), [IL11](https://www.affymetrix.com/LinkServlet?probeset=IL11), [IFNW1](https://www.affymetrix.com/LinkServlet?probeset=IFNW1), [IFNA4](https://www.affymetrix.com/LinkServlet?probeset=IFNA4), [GH1](https://www.affymetrix.com/LinkServlet?probeset=GH1), [GH2](https://www.affymetrix.com/LinkServlet?probeset=GH2), [IL13](https://www.affymetrix.com/LinkServlet?probeset=IL13), [IFNA21](https://www.affymetrix.com/LinkServlet?probeset=IFNA21), [SOCS3](https://www.affymetrix.com/LinkServlet?probeset=SOCS3), [PIAS2](https://www.affymetrix.com/LinkServlet?probeset=PIAS2), [GHR](https://www.affymetrix.com/LinkServlet?probeset=GHR), [IFNA2](https://www.affymetrix.com/LinkServlet?probeset=IFNA2), [IFNA5](https://www.affymetrix.com/LinkServlet?probeset=IFNA5), [CTF1](https://www.affymetrix.com/LinkServlet?probeset=CTF1), [EPO](https://www.affymetrix.com/LinkServlet?probeset=EPO), and [IFNA16](https://www.affymetrix.com/LinkServlet?probeset=IFNA16) |
| Type II diabetes mellitus | -1.50E+00 | 3.59E-02 | 1.62E-01 | [MAPK10](https://www.affymetrix.com/LinkServlet?probeset=MAPK10), [PKLR](https://www.affymetrix.com/LinkServlet?probeset=PKLR), [IRS2](https://www.affymetrix.com/LinkServlet?probeset=IRS2), [INSR](https://www.affymetrix.com/LinkServlet?probeset=INSR), [PRKCE](https://www.affymetrix.com/LinkServlet?probeset=PRKCE), [CACNA1C](https://www.affymetrix.com/LinkServlet?probeset=CACNA1C), [CACNA1B](https://www.affymetrix.com/LinkServlet?probeset=CACNA1B), [HK2](https://www.affymetrix.com/LinkServlet?probeset=HK2), [TNF](https://www.affymetrix.com/LinkServlet?probeset=TNF), [GCK](https://www.affymetrix.com/LinkServlet?probeset=GCK), [PIK3CB](https://www.affymetrix.com/LinkServlet?probeset=PIK3CB), [PRKCZ](https://www.affymetrix.com/LinkServlet?probeset=PRKCZ), [CACNA1A](https://www.affymetrix.com/LinkServlet?probeset=CACNA1A), [IRS4](https://www.affymetrix.com/LinkServlet?probeset=IRS4), [PDX1](https://www.affymetrix.com/LinkServlet?probeset=PDX1), [CACNA1G](https://www.affymetrix.com/LinkServlet?probeset=CACNA1G), [PIK3R2](https://www.affymetrix.com/LinkServlet?probeset=PIK3R2), [HK3](https://www.affymetrix.com/LinkServlet?probeset=HK3), [PIK3R5](https://www.affymetrix.com/LinkServlet?probeset=PIK3R5), [ADIPOQ](https://www.affymetrix.com/LinkServlet?probeset=ADIPOQ), [SOCS3](https://www.affymetrix.com/LinkServlet?probeset=SOCS3), [INS](https://www.affymetrix.com/LinkServlet?probeset=INS), [MAPK3](https://www.affymetrix.com/LinkServlet?probeset=MAPK3), [MTOR](https://www.affymetrix.com/LinkServlet?probeset=MTOR), and [MAPK8](https://www.affymetrix.com/LinkServlet?probeset=MAPK8) |

**
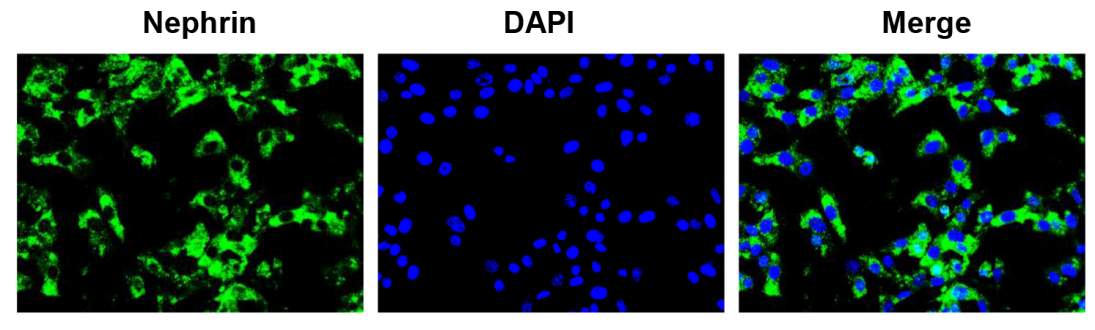
**

**Supplementary Figure 1.** **Immunofluorescent staining for Nephrin protein in cultured podocytes (×200).**
